# *In vivo* demonstration of enhanced mRNA delivery by cyclic disulfide-containing lipid nanoparticles for facilitating endosomal escape†

**DOI:** 10.1039/d5md00084j

**Published:** 2025-06-27

**Authors:** Seigo Kimura, Kana Okada, Noriaki Matsubara, Fangjie Lyu, Susumu Tsutsumi, Yasuaki Kimura, Fumitaka Hashiya, Masahito Inagaki, Naoko Abe, Hiroshi Abe

**Affiliations:** a Integrated Research Consortium on Chemical Sciences (IRCCS), Nagoya University Furo-cho, Chikusa-ku Nagoya Aichi 464-8602 Japan kimura.seigo.v9@f.mail.nagoya-u.ac.jp +81 052 789 2947 +81 052 789 2490; b Department of Chemistry, Graduate School of Science, Nagoya University Furo-cho, Chikusa-ku Nagoya Aichi 464-8602 Japan abe.hiroshi.p4@f.mail.nagoya-u.ac.jp +81 052 789 2947 +81 052 789 2490; c Institute for Glyco-core Research (iGCORE), Nagoya University Nagoya Aichi 464-8601 Japan

## Abstract

Current LNP technology faces challenges that must be addressed to enhance the functionality of mRNA therapeutics. Recent studies show disulfide-conjugated molecules improve cell membrane permeability. Here, we investigated incorporating cyclic disulfide (CDL) units into lipid components of LNPs to enhance LNP-mRNA performance. A lipid library with branched and unbranched alkyl chains (C16–C20) and tertiary amine groups modified with CDLs was designed. While cellular uptake was unchanged, some mRNA-loaded LNPs with CDLs achieved more than 2-fold higher transfection efficiency than LNPs with MC3 or SM102 alone. Intracellular analysis revealed that the addition of CDL lipids significantly promoted endosomal escape. The CDL-incorporated LNPs administered subcutaneously in mice showed significantly higher luciferase gene expression compared to LNPs without CDL. Additionally, LNPs encapsulating OVA antigen-encoding mRNA induced a potent antitumor response against the EG7-OVA lymphoma model. These results suggest CDL modifications enhance LNP-based mRNA delivery, offering potential for broader therapeutic applications and improved clinical outcomes.

## Abstract 2

The incorporation of cyclic-disulfide containing lipid (CDL) into the LNP composition enhances the functional delivery of mRNA by promoting endosomal escape.

## Graphical abstract

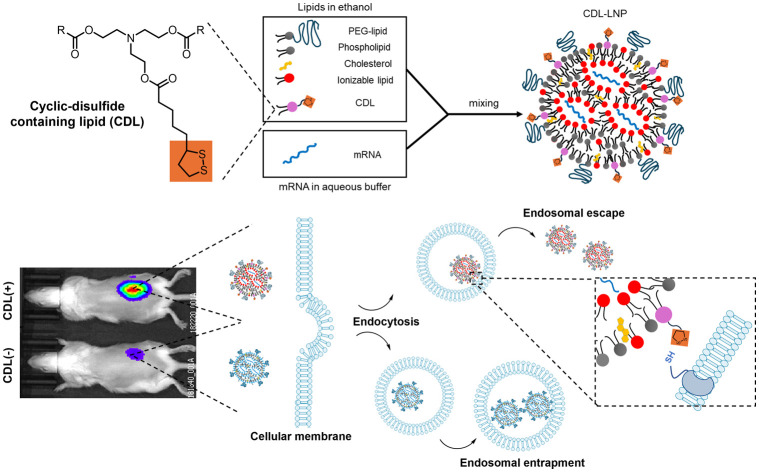

## Introduction

mRNA therapeutics have attracted considerable attention in vaccine development against infectious diseases, and this interest has intensified following the successful deployment of COVID-19 vaccines.^1,2^ Moreover, mRNA has broad applications beyond vaccines, including cancer immunotherapy,^3–5^ protein replacement therapy,^6,7^*in vivo* production of antibody therapeutics,^8–10^ and genome editing.^11^ However, nucleic acid molecules, including mRNA, inherently face challenges due to their negative charge, which limits membrane permeability, as well as their susceptibility to enzymatic degradation. Consequently, effective delivery systems are essential to ensure efficient uptake into target cells. To address these issues, extensive efforts have focused on developing modified nucleic acids and advanced delivery carriers. In particular, lipid nanoparticles LNPs and polymer nanoparticles are widely utilized as delivery vehicles for mRNA therapeutics.^12,13^ Among these, lipid-based carriers—especially LNPs—are recognized as one of the most advanced systems for delivering nucleic acid-based therapeutics.^14,15^

A typical LNP is composed of ionizable lipids, phospholipids, cholesterol (Chol), and polyethylene glycol (PEG)-lipids. The ionizable lipids, which feature structures such as tertiary amines, become positively charged under acidic conditions. Within the acidic intracellular environments of endosomes and lysosomes, these ionizable lipids electrostatically interact with the negatively charged endosomal membranes, thereby facilitating the release of nucleic acids into the cytoplasm. Because the combination and ratio of each lipid component in an LNP determine its structural and biological properties, extensive screening of lipid structures and compositions has been conducted to optimize LNP performance.^16–25^ Nonetheless, the escape efficiency from endosomes and lysosomes remains low, with only a few percent of internalized nucleic acids reaching the cytoplasm.^26^ Furthermore, excessive inflammatory responses attributed to LNPs have been suggested as a factor underlying adverse reactions to mRNA vaccines,^27^ highlighting the urgent need to improve both the delivery efficiency and safety of LNPs.

On the other hand, it has been reported that the introduction of disulfide bonds into molecules enhances their cell membrane permeability. Previous studies have demonstrated that disulfide modification of various substrates—including small molecular fluorescent probes, peptides, proteins, and nucleic acids—can promote membrane penetration.^28–30^ Moreover, cases have been documented in which disulfide-modified nucleic acids enter the cytoplasm directly, bypassing endocytosis.^31^ Although the exact mechanism remains unclear, it is hypothesized that disulfide exchange reactions between the disulfide moieties on oligonucleotides and proteins on the cell membrane facilitate internalization.^32^ Furthermore, some nanoparticle delivery systems have shown that disulfide modifications on the nanoparticle surface improve intracellular uptake^33,34^ and cytoplasmic localization.^35,36^

In the field of LNP-mediated nucleic acid delivery, strategies incorporating disulfide motifs into the lipid structure have already been reported.^37–41^ These approaches typically introduce a disulfide bond into the alkyl chain of the lipid molecule, with the primary aim of targeting the reductive intracellular environment, where disulfide bonds are cleaved. This process leads to lipid degradation, collapse of the LNP structure, and subsequent release of the payload. However, this action differs from the mechanism by which interactions between thiols and disulfide bonds on the nanoparticle surface promote endosomal escape. Interestingly, cyclic disulfides have been shown to facilitate the intracellular uptake of large substrates such as liposomes and polymersomes with diameters up to 400 nm.^33,34^ Although the thiol-mediated uptake mechanism is not fully understood, it is believed that interactions between surface disulfides on nanoparticles and cell surface thiols enhance cellular internalization.^32^ Furthermore, confocal microscopy analyses suggest that disulfide bonds promote the endosomal escape of nanogels,^35^ and studies examining interactions with erythrocytes have revealed that thiolated lipid-based nanoparticles can enhance endosomal escape.^36^ However, to the best of our knowledge, there are no reported examples of achieving functional *in vivo* mRNA delivery using this approach.

In this study, we aimed to improve mRNA delivery efficiency and protein expression by incorporating newly designed disulfide-containing lipids (CDLs) into LNPs. We have previously demonstrated that the use of disulfide bonds, such as in membrane permeable oligonucleotide (MPON), can facilitate intracellular translocation and promote endosomal escape.^31,42^ Here, we synthesized a series of cyclic disulfide-containing lipids (CDLs) with varying alkyl chain lengths and evaluated their ability to deliver mRNA intracellularly when formulated into LNPs. This design, in which a cyclic disulfide unit is attached to the tertiary amines in the headgroup of the ionizable lipid and incorporated into LNPs, is distinct from the conventional approach of introducing disulfide bonds into the fatty acid chains of lipid molecules. *In vitro* transfection assays showed that certain CDLs enhanced transfection efficiency, depending on the type of ionizable lipid used in the LNP. To elucidate the mechanisms underlying these improvements, we compared the intracellular trafficking of various LNP formulations. Finally, we applied this system in a cancer vaccine model and demonstrated functional mRNA delivery in mice.

## Results

### Design and synthesis of cyclic disulfide-containing lipids

**Fig. 1:**
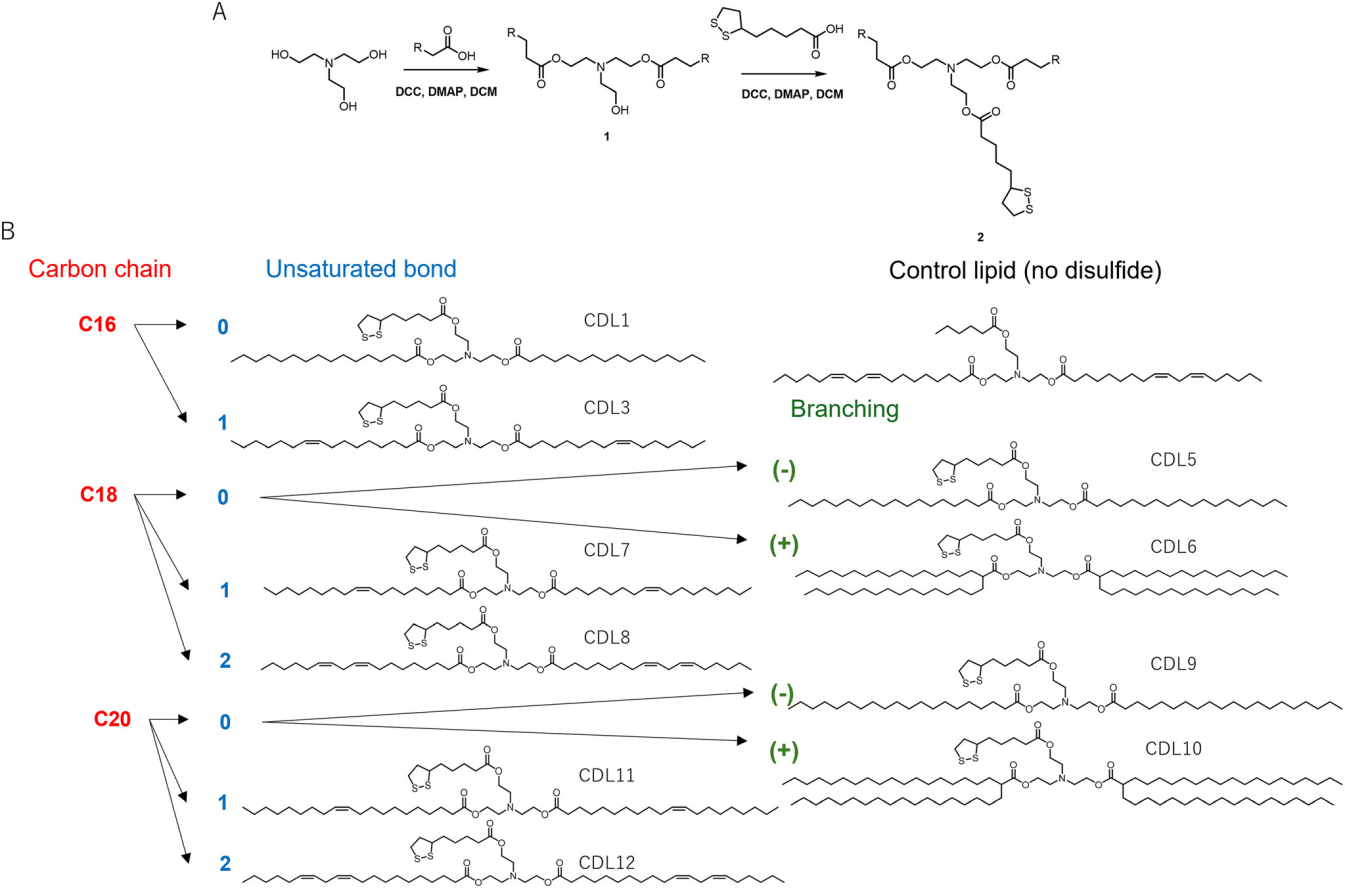
Design and synthesis of CDLs. (A) Synthesis scheme, DCC; *N*,*N*′-dicyclohexylcarbodiimide; DCM, dichloromethane DMAP; 4-dimethylaminopyridine, R; alkyl residue; (B) synthesized CDLs and control lipid without cyclic disulfide structure.

To introduce a disulfide moiety into the LNPs, disulfide-containing lipids derived from α-lipoic acid were designed and synthesized. α-Lipoic acid was chosen for several reasons: cyclic disulfides with high ring tension, as found in α-lipoic acid, exhibit enhanced thiol-mediated membrane permeability compared to linear disulfides.^43^ Additionally, α-lipoic acid is a naturally occurring compound synthesized by plants and animals, including humans,^44,45^ and is considered to have low cytotoxicity. In a previous publication, Qualls *et al.* applied lipids containing a cyclic disulfide moiety derived from α-lipoic acid for the intracellular delivery of liposomes.^34^ They incorporated the disulfide unit into the amine group of the hydrophilic head of DOPE. In contrast, based on the finding that combining different types of ionizable lipids can enhance mRNA delivery efficiency,^23,46^ we designed lipids containing a tertiary amine structure (Fig. 1). The synthesis of cyclic disulfide-containing lipids (CDLs) was carried out using a two-step reaction (Fig. 1A). Detailed methods for the synthesis of each CDL are provided in the ESI† Methods. Triethanolamine was used as the starting material, and 11 fatty acids were employed, varying in carbon chain length (C16, C18, or C20), number of unsaturated bonds (0, 1, or 2), and the presence or absence of branching. Compound 1 was synthesized by condensing each fatty acid with *N*,*N*-dicyclohexylcarbodiimide (DCC) and 4-dimethylaminopyridine (DMAP). Subsequently, α-lipoic acid and compound 1 were condensed using DCC and DMAP to yield the target compound 2. Hexanoic acid was used as a control, replacing α-lipoic acid to synthesize a compound where the disulfide moiety was substituted with a carbon chain. The CDL library consisted of 10 CDLs and one control lipid (Ctrl) (Fig. 1B, Table S1†).

### Effect of different CDLs on the mRNA-LNP formulation

**Fig. 2:**
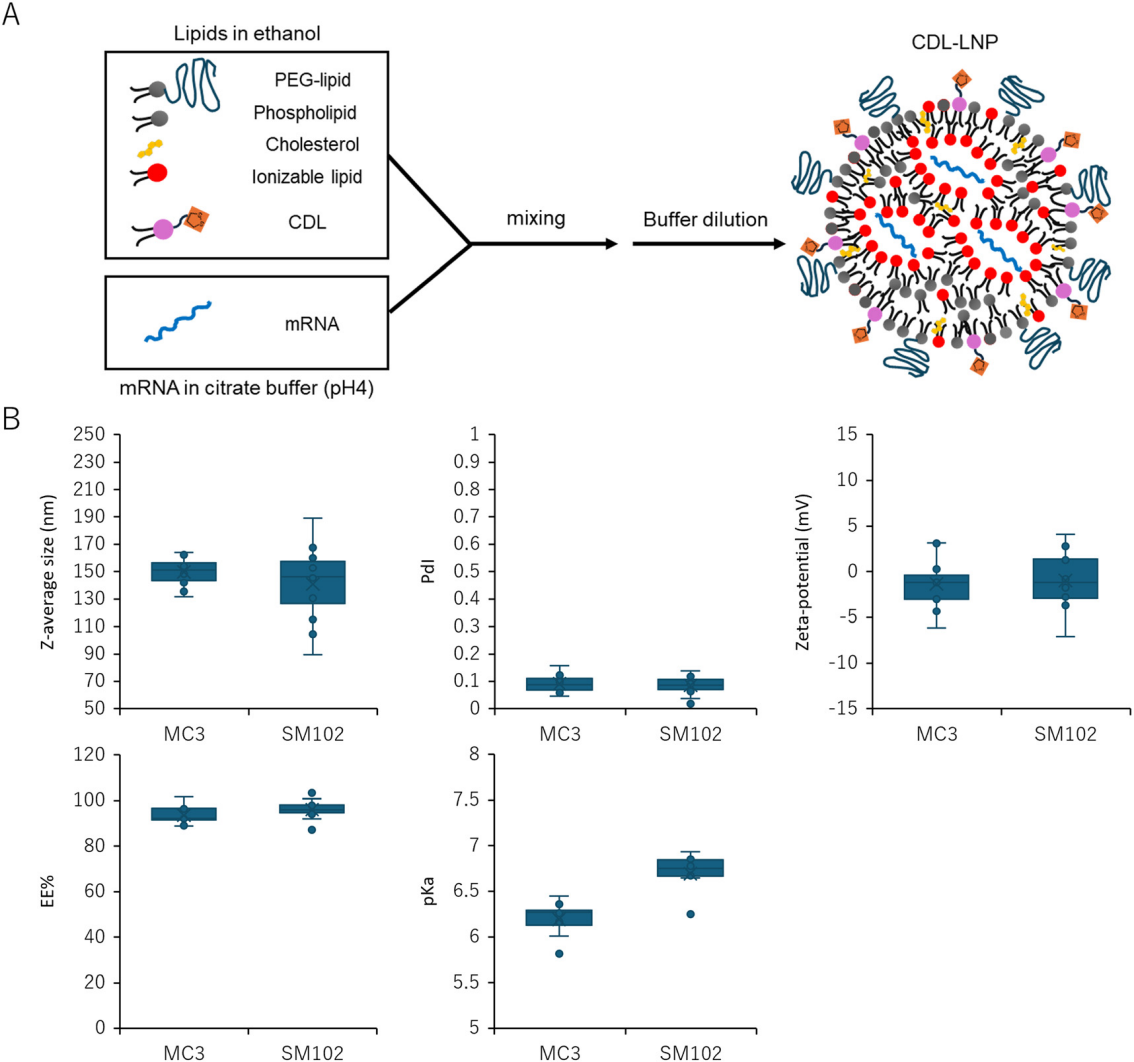
Characterization of mRNA-loaded LNPs containing different CDLs. (A) Schematic depiction of the LNP preparation. (B) Properties of various LNPs categorized by the ionizable lipids to be used (*Z*-average size, polydispersity index (PDI), zeta potential, mRNA encapsulation efficiency (EE), and apparent p*K*_a_ value) are shown in box plots. Each dot represents an LNP (mean, *N* = 2–3).

mRNA-loaded LNPs were formulated using an ethanol dilution method. LNPs are typically composed of four components: ionizable cationic lipid, phospholipid, cholesterol, and PEG-lipid. However, in this study, following approaches outlined in previous publications, cyclic disulfide-containing lipids (CDLs) were incorporated as a fifth component to prepare CDL-LNPs (Fig. 2A). The lipid molar ratios followed the general composition used in clinical applications such as Onpattro and Spikevax, with CDL (or Ctrl) constituting 20 mol% of the total lipid content (Table 1). MC3 or SM102 served as the ionizable cationic lipid in this study.

**Table 1:** Molar ratios for mRNA-LNP formulations with or without CDLs

Lipid	With CDL	Without CDL
MC3 or SM102	30	50
CDL or Ctrl	20	0
DOPE	10	10
Chol	38.5	38.5
DMG-PEG2k	1.5	1.5

Fig. 2B summarizes the characterization of 24 different LNPs, with detailed parameters of each LNP provided in Table S2.† The diameters ranged from 100 to 190 nm, with an average of approximately 150 nm, and a polydispersity index (PdI) of less than 0.2, indicating a narrow particle size distribution. All LNPs exhibited a neutral surface charge (−5 to 5 mV) at physiological pH 7.4, and mRNA encapsulation efficiencies (EE) were consistently above 85%. The p*K*_a_ values were primarily influenced by the ionizable lipid in the LNP formulations, although changes in the alkyl tail structure of CDLs (MC3: 6.0–6.5, SM102: 6.5–7) shifted these values. This suggests that both the head group and the alkyl residue structure contribute to the p*K*_a_.

### Assessment of LNP functions for mRNA delivery in *in vitro* study

**Fig. 3:**
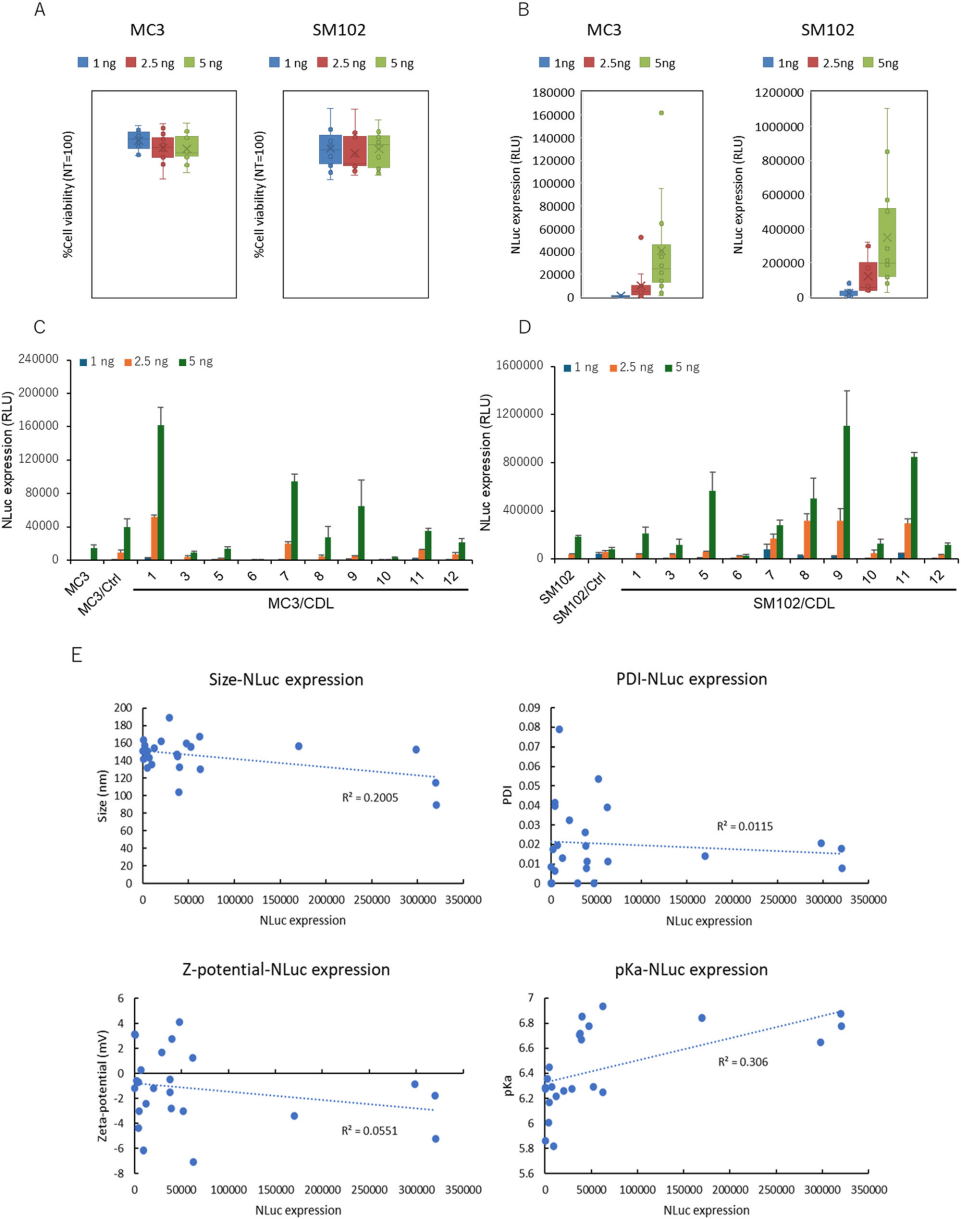
Effect of CDLs on *in vitro* transfection efficiency of mRNA-LNP formulations. Box plots for cell viability (A) and NLuc expression (B). Each plot represents the mean value of an individual LNP. (C and D) Bar graphs for NLuc expression of both types of LNP formulations, MC3 and SM102-LNPs, with different CDLs. Each bar shows the mean value of 3 different experiments. (E) Correlations between physicochemical characterizations of LNPs and mRNA transfection activity. Dot plots comparing LNP size, PDI, zeta potential, and p*K*_a_*vs.* NLuc expression. Each plot represents the mean value of 3 different experiments.

*In vitro* transfection assays were conducted to evaluate whether differences in CDLs affected the efficiency of mRNA transfection. Cell viability and nanoluciferase (NLuc) activity of each LNP formulation were assessed using WST-8 and NLuc assays, respectively. Across all formulations, the cell viability remained above 80%, indicating no significant cytotoxicity within the tested dose range (Fig. 3A). Conversely, NLuc activity varied with lipid composition, specifically with the presence, absence, and structure of the CDLs (Fig. 3B–D and S1†). For MC3 formulations (MC3-LNP), translation activity increased by more than 1.9-fold compared to that of MC3 alone when loaded with CDL1, CDL7, CDL8, CDL9, or CDL11. CDL1 exhibited up to 10-fold higher activity than MC3 alone (Fig. 3C). In the SM102 formulations (SM102-LNP), CDL5, CDL7, CDL8, CDL9, and CDL11 showed more than a 1.5-fold increase in expression compared to SM102 alone, with CDL9 demonstrating the highest enhancement (∼6-fold) (Fig. 3D). The optimal CDL type varied depending on the ionizable lipids (MC3 and SM102) in the LNP formulation (Fig. S1†). The higher activity observed with CDL-containing LNPs compared to control lipids lacking cyclic disulfides suggests that the introduction of cyclic disulfide structures contributes to increased translational activity. The type of phospholipid used affects the efficiency of LNP delivery.^47,48^ To investigate how the type of phospholipid influences the enhanced activity observed with CDL, we measured transfection activity using DSPC, a phospholipid commonly used alongside DOPE. The results showed that the addition of CDL enhanced transfection activity regardless of the phospholipid used, and NLuc activity was higher in DOPE-based formulations compared to DSPC-based formulations. This difference may be attributed to the higher membrane fusogenicity of DOPE compared to DSPC (Fig. S2†). To explore the relationship between LNP characterization and transfection activity, correlations between the physicochemical properties of LNPs (particle size, PDI, zeta potential, and p*K*_a_) and translational activity were evaluated (Fig. 3E). Notably, no significant correlation between LNP characterization and NLuc expression activity was observed, supporting the notion that cyclic disulfide structures enhance activity independently of other LNP characteristics.

### Cellular uptake and lysosomal localization of CDL-containing LNPs

**Fig. 4:**
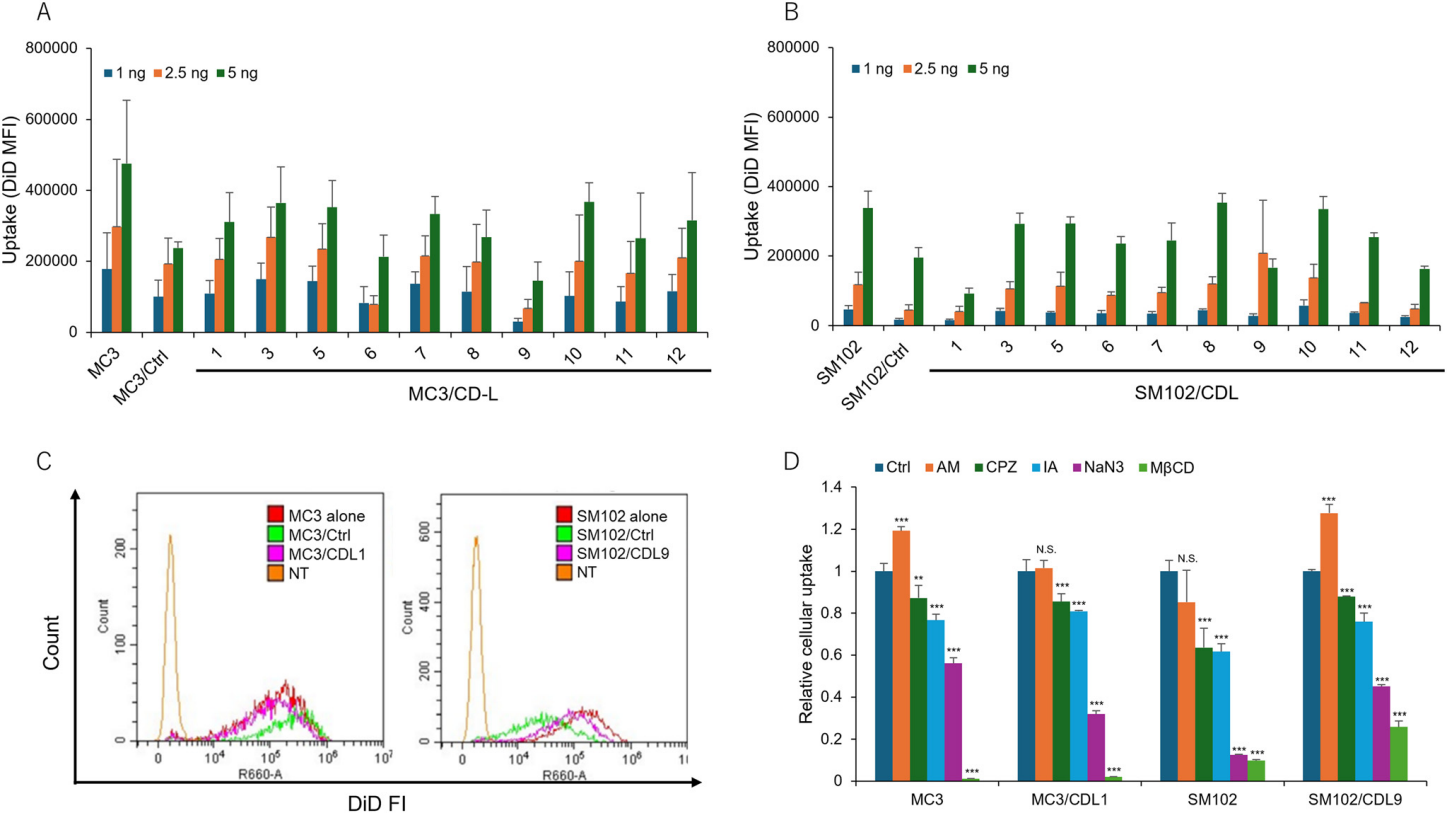
Comparison of cellular uptake of different LNPs. (A and B) DiD mean fluorescence intensity (MFI) of cells after treatment with different CDL-containing LNPs. Each bar represents the mean + SD of 3 different experiments. (C) Histograms of fluorescence intensities for comparing MC3 alone *vs.* MC3/Ctrl *vs.* MC3/CDL1, or SM102 alone *vs.* SM102/Ctrl *vs.* SM102/CDL9. (D) Investigation of the internalization pathways of different LNPs using different inhibitors. Relative cellular uptake was calculated as the ratio *vs.* Ctrl. The following inhibitors were used at their respective concentrations. AM: 20 μM amiloride, pinocytosis inhibitor, CPZ: 10 μM chlorpromazine, clathrin-mediated endocytosis inhibitor, IA: 1.2 μM iodoacetate, NaN_3_: 50 mM sodium azide, energy-dependent pathway inhibitor, MβCD; 2.5 mM methyl-β-cyclodextrin, deplete cholesterol. Each bar represents the mean + SD of 3 different experiments. **p < 0.01, ***p < 0.001 *vs.* Ctrl.

To investigate the mechanism underlying the enhanced transfection activity achieved by incorporating CDLs into LNP formulations, we assessed the cellular uptake of DiD-labeled LNPs using flow cytometry. Despite observing increased protein production, the uptake of LNPs did not show significant variation based on the structure of CDLs or the type of ionizable lipid used (Fig. 4A and B). Representative histograms comparing MC3 or SM102 alone with MC3/Ctrl or SM102/Ctrl, and MC3/CDL1 or SM102/CDL9 are shown in Fig. 4C. These histograms illustrate minimal differences in cellular uptake, despite the observed increase in translation activity when using CDL1 for MC3-LNP and CDL9 for SM102-LNP. Moreover, Fig. S3† demonstrates the absence of a correlation between cellular uptake and NLuc expression, suggesting that factors beyond uptake, such as endosomal escape mechanisms,^49^ play a pivotal role in enhancing translation activity. To investigate the role of specific endocytosis pathways, cells were treated with different inhibitors before transfection to evaluate their effects on the cellular uptake of MC3-LNP, MC3/CDL1-LNP, SM102-LNP, and SM102/CDL9-LNP (Fig. 4D). The following inhibitors were used: amiloride (AM), chlorpromazine (CPZ), iodoacetate (IA), sodium azide (NaN_3_), and methyl-β-cyclodextrin (MβCD), which inhibit pinocytosis,^50,51^ clathrin-mediated endocytosis,^52^ thiol-mediated pathway,^31^ energy-dependent endocytosis,^53,54^ and cholesterol-dependent lipid rafting,^55^ respectively. Most of these inhibitors affected cellular uptake, with NaN_3_ and MβCD treatments showing higher inhibition than other treatments in all LNP formulations. This indicates that multiple internalization pathways contribute to LNP uptake, with energy- and cholesterol-dependent lipid rafting playing a prominent role. In MC3-LNP and SM102/CDL9-LNP, AM treatment led to higher uptake compared to the control (Ctrl), possibly due to increased uptake through non-pinocytic pathways when treated with AM. However, there was no significant differences in uptake pathways between the presence and absence of CDLs, except for some variations in the inhibitory effects.

**Fig. 5:**
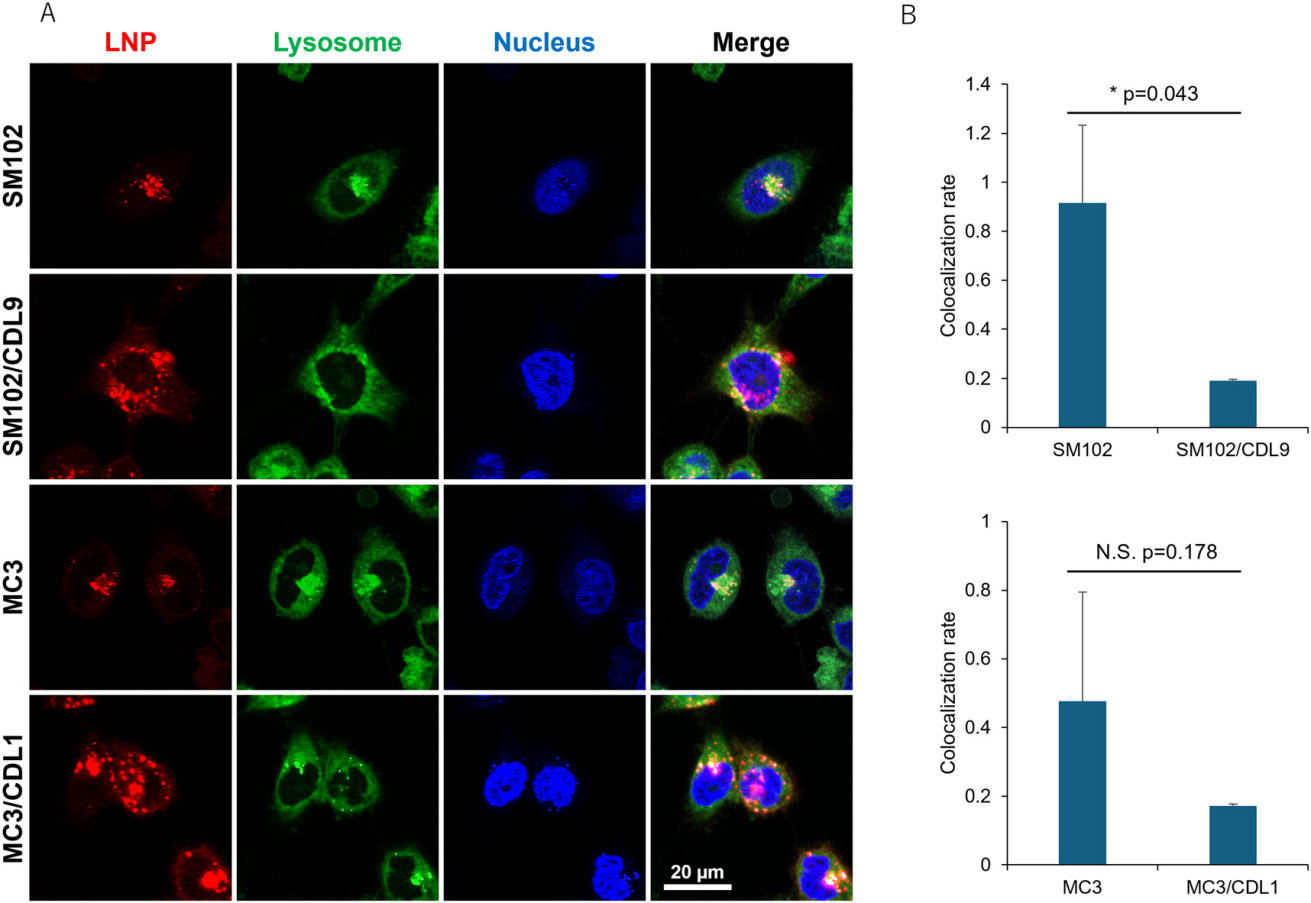
Lysosomal localization of different LNPs. (A) Representative images of cells treated with different DiD-labelled LNPs. Red: LNPs (DiD), green: lysosomes (Lysosensor Green), blue: nucleus (Hoechst 33342), yellow: co-localization of LNP and lysosomes. (B) Lysosomal colocalization rates of LNPs were calculated by dividing the area of the yellow dot by the area of the red dot. Each bar represents the mean ± SD of 3 different experiments. *p < 0.05.

Since the difference in cellular uptake cannot explain the increase in translation activity when CDLs are added, lysosomal localization of LNPs was examined by fluorescence imaging using confocal microscopy. Representative images of observed cells are shown in Fig. 5A. The pattern of colocalization between LNPs and lysosomes differed between the non-CDL and CDL-containing formulations. Lower colocalization with lysosomal compartments was observed in MC3 and SM102 formulations with CDL1 and CDL9, respectively. In contrast, MC3 or SM102 alone showed a higher number of LNPs localized to lysosomes (yellow dots), indicating significant entrapment of LNPs within the lysosomal compartment (Fig. 5A, S4A and B†). These results demonstrate that CDLs can enhance the endosomal escape of LNPs. The lysosomal colocalization rate was calculated by dividing the area of the yellow dot (representing the colocalization of LNPs and lysosomes) by the area of the red dot (representing LNP localization outside the lysosomal compartments) (Fig. 5B). The addition of CDLs decreased the lysosomal colocalization rate, suggesting increased endosomal escape. Line profiles evaluating the overlap between LNPs and lysosomes also indicated lower colocalization for CDL-containing LNP formulations compared to non-CDL formulations (Fig. S4C and D†). We next investigated whether the incorporation of CDL into LNPs enhances membrane fusion. Hemolysis assays were performed to directly compare LNPs with and without CDL (Fig. S5†). We evaluated SM102 alone (SM102), a control lipid lacking a cyclic disulfide (SM102/Ctrl), and CDL-containing LNPs (SM102/CDL) under both 0% and 10% serum conditions. In the absence of serum, SM102 exhibited greater hemolytic activity (∼68%) than both SM102/Ctrl and SM102/CDL at pH 6.5–4.5, whereas at pH 7.5, SM102/CDL showed higher activity than the other formulations. Under 10% serum conditions, which better approximate the transfection environment, hemolytic activities of all samples decreased sharply. Nevertheless, SM102/CDL exhibited higher hemolytic activity than the controls at both pH 7.5 and 6.5. These findings suggest that even in the presence of serum, CDL-containing LNPs may facilitate closer interactions with membranes, potentially enhancing endosomal escape.

### *In vivo* delivery of mRNA by CDL-containing LNPs and its application for cancer vaccine

**Fig. 6:**
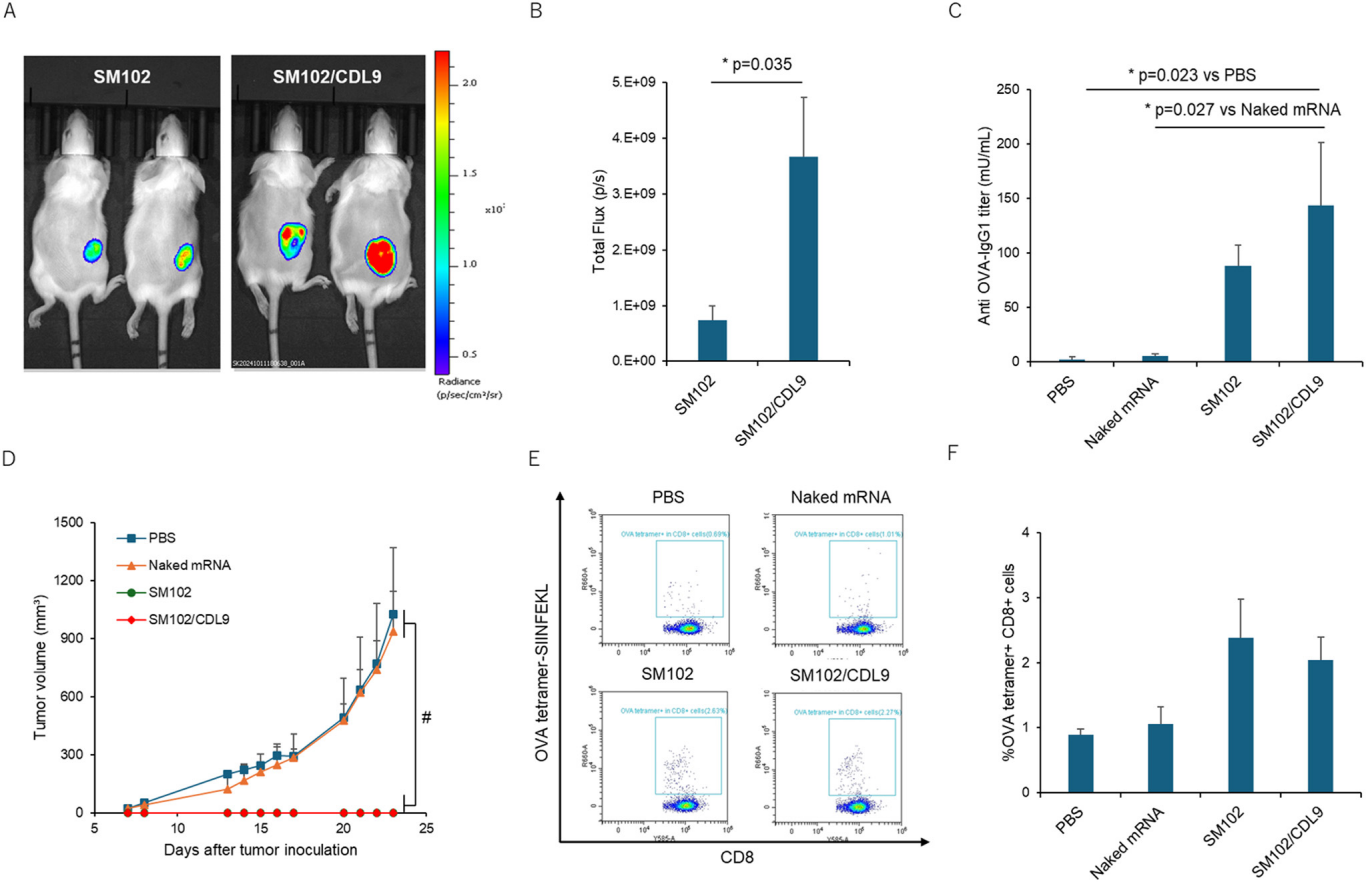
*In vivo* comparison of SM102-LNP and SM102–CDL9-LNP. (A) Representative images of mice injected with 4 μg of Nluc mRNA formulated with LNPs *via* subcutaneous route of administration. (B) Total flux (p s^−1^) of mice injected with 4 μg of mRNA encoding Nluc formulated with LNPs for subcutaneous route (mean + SE of *N* = 3–4). (C) Antigen-specific antibody levels in the blood. Anti-OVA IgG1 titers were measured by ELISA (mean + SE of *N* = 5, *p < 0.05). (D) Prophylactic anti-tumor effect: C57BL6N mice were treated with PBS, OVA-encoding naked mRNA, OVA-encoded mRNA formulated with SM102-LNP or SM102/CDL9-LNP at 7 and 14 days before tumor inoculation. The mice were inoculated with E.G7-OVA cells and tumor volume was monitored. The plots represent the mean + SE (total of 5 mice/group) (#: *p < 0.05 day 8, 15–23, SM102 or SM102/CDL9 *vs.* PBS or naked mRNA, **p < 0.01 day 13, SM102 or SM102/CDL9 *vs.* PBS or naked mRNA). (E) Representative dot plots for detecting splenic antigen-specific cytotoxic CD8+ T cells. The gating shown in light blue in the dot plot indicates OVA tetramer-positive cells within the CD8-positive population. (F) The percentage of splenic antigen-specific CD8+ T cells (mean + SE of *N* = 5).

Finally, we evaluated *in vivo* functional mRNA delivery using CDL-containing LNPs. LNPs were administered subcutaneously to the dorsal surface of mice, and *in vivo* imaging was conducted 6 hours post-administration to measure NLuc expression. As in the *in vitro* study, CDL9-containig SM102-LNP formulation showed a higher functional mRNA delivery than the SM102-LNP formulation without CDL9 (Fig. 6A and B), suggesting that the disulfide strategy is effective *in vivo*. Next, we examined the efficacy of this LNP formulation as an mRNA vaccine. Using ovalbumin (OVA) as a model antigen, mice were immunized twice with LNPs containing OVA-encoding mRNA (OVA-mRNA) *via* subcutaneous administration, and the level of OVA-specific IgG1 in the serum was measured. The SM102/CDL9-treated samples showed a higher OVA-specific IgG1 level compared to the PBS- and naked mRNA-treated samples (Fig. 6C). Although not statistically significant, SM102/CDL9 produced higher antibody levels than SM102 alone (Fig. 6C), which may be attributed to the increased functional mRNA delivery observed in Fig. 6B. The potential of the LNP formulation for cancer vaccination was assessed in a mouse model using OVA-expressing cancer cells, E.G7-OVA. Mice were immunized with two subcutaneous injections of LNPs encapsulating OVA-mRNA at 1-week intervals. 8 days after the second injection, E.G7-OVA cells were inoculated, and tumor volumes were monitored for 23 days. Tumor volumes in mice treated with naked OVA-mRNA were comparable to those of PBS-treated mice while inhibition of tumor growth were observed in some mice (Fig. 6D and S4†). In contrast, both the SM102 and SM102/CDL9 groups completely inhibited tumor growth (Fig. 6D and S6†), suggesting that LNP-mRNA induces an efficient antigen-specific immune response against the tumor. The antigen-specific CD8 positive cells in the spleen were measured 24 days after tumor inoculation. The mean percentage of splenic OVA-specific CD8 positive cells were higher in the LNP-treated groups compared to the PBS- and naked mRNA-treated groups, although the difference was not statistically significant (Fig. 6E and F). Finally, a single-dose toxicity assessment was conducted by measuring changes in body weight and serum cytokine levels (IL-6, TNFα, and IFNβ) following subcutaneous administration of LNP-mRNA in mice (Fig. S8A†). No significant weight loss was observed from the time of administration up to two days post-injection (Fig. S8B†). IL-6 levels were comparable to or lower than those in the PBS group, regardless of the presence of CDL in the LNPs (Fig. S8C†), and TNFα and IFNβ were below the detection limits in both groups. These findings suggest that the incorporation of CDL does not negatively impact *in vivo* tolerability. These findings suggest that SM102/CDL9-encapsualted antigen mRNA induced potent humoral and cellular immune responses against the tumor, which are comparable to or slightly higher than those induced by SM102 without CDL9.

## Discussion

Further advancements in delivery systems are crucial for broadening the future applications of mRNA therapeutics. Although LNPs represent the current gold-standard carrier for non-viral nucleic acid delivery, they have notable limitations, particularly regarding safety concerns such as inflammation^27^ and cytokine storms.^56^ Therefore, optimizing LNP-based delivery remains essential to achieving dose reduction and enhancing clinical relevance.

Previous studies have demonstrated that disulfide-based strategies can enhance the cellular delivery of a wide range of molecules, from small molecules to macromolecules, including peptides, proteins, nucleic acids, and nanoparticles.^28–31,33–35^ However, most of these studies have been conducted *in vitro*, and there are no reported cases of *in vivo* functional mRNA delivery using this approach. This raises a critical question: Can the disulfide strategy improve LNP-mediated mRNA delivery both *in vitro* and *in vivo*? We present evidence that cyclic disulfide modifications can enhance functional mRNA delivery by incorporating cyclic disulfide-containing lipids (CDLs) into LNP formulations. CDLs were synthesized from triethanolamine *via* two main steps involving DCC/DMAP-mediated condensation reactions with various fatty acids and α-lipoic acid—significantly fewer steps than those reported for other clinically relevant ionizable lipids such as MC3, ALC-0315, and SM-102.^57–59^ The overall yield of CDL synthesis depends on the structure of the hydrophobic (fatty acid) chains and ranges from 19% to 86% (Table S1†), which is comparable to the reported yields for ALC-0315 and SM-102. The synthetic route utilizes standard organic reactions commonly employed in medicinal chemistry, and all reagents are commercially available. Consequently, CDL synthesis does not require specialized reagents or equipment, suggesting favorable scalability and manufacturing compatibility. By testing a library of CDLs, our initial *in vitro* findings suggest that introducing cyclic disulfides improves the efficiency of functional mRNA delivery (Fig. 3). Key physicochemical parameters of LNPs, such as particle size, surface charge, and apparent p*K*_a_, significantly influence their biological behavior and delivery efficiency. While some variations were observed depending on the CDL structure, no substantial differences in the physical properties of LNPs were detected (Fig. 2 and Table S2†). Nonetheless, LNPs containing cyclic disulfides exhibited higher activity than those with control lipids lacking such modifications (Fig. 3C and D), suggesting that the cyclic disulfide structure itself, rather than the macroscopic physical properties of the LNPs, contributes to the enhanced activity. Our data (Fig. 3C, D and S1†) indicate that the optimal CDL structure depends on the ionizable lipids used in the LNP formulation, with CDL1 and CDL9 being optimal for MC3 and SM102, respectively. This variability may result from differences in the interactions between CDLs and specific ionizable lipids, which likely affect the presentation of cyclic disulfides on the LNP surface. Indeed, transfection efficiency is influenced by the combination of ionizable and helper lipids (*e.g.*, phospholipids),^16,47,48,60^ and molecular simulation analyses suggest that lipid–lipid interactions modulate ionizable lipid diffusion, thereby altering transfection efficiency.^61^

An additional unanswered question we hope to address is the mechanism by which the incorporation of CDLs into LNP compositions enhances functional delivery. To explore this, we conducted flow cytometry analyses comparing the cellular uptake of LNPs with and without CDLs. Interestingly, unlike prior reports on disulfide-based cellular delivery, CDLs did not enhance LNP uptake (Fig. 4A and B). Despite a 6- to 10-fold difference in transfection activity (Fig. 3C and D), the uptake levels were comparable (Fig. 4C). Previous studies on LNP-mediated nucleic acid delivery suggest the involvement of specific uptake pathways that facilitate effective functional delivery of mRNA and plasmid DNA (pDNA).^62–64^ For example, macropinocytosis has been implicated in efficient mRNA-LNP delivery.^62,63^ While the exact mechanisms remain under investigation, several hypotheses have been proposed: macropinocytosis may reduce LNP clearance *via* recycling endosomes;^65^ macropinosomes may acidify at a more favorable rate than early endosomes, allowing fusion with lysosomes and facilitating endosomal escape before degradation;^66^ or macropinocytosis might activate the mTOR pathway, enhancing translational efficiency.^67^ Additionally, pathways mediated by apolipoprotein E (ApoE) and CD21/CD35 have been identified as productive routes for delivering pDNA to the liver and spleen, respectively. These pathways likely elicit distinct cellular responses based on the uptake mechanism, thereby influencing the intracellular dynamics of pDNA-loaded LNPs.^64^ Based on these observations and our data, we hypothesized that cellular uptake pathways play a critical role in functional mRNA delivery. To test this hypothesis, we performed inhibition experiments targeting various endocytic pathways (Fig. 4D). Unlike prior studies on disulfide-based strategies,^31,68^ where thiol masking inhibited uptake, CDL-containing LNPs did not exhibit greater inhibition compared to LNPs without CDLs. A previous study on cyclic disulfide-functionalized liposomes reported increased uptake when the CDL content exceeded 60%.^34^ In the present study, the CDL content was relatively low (20%), which may explain the minimal impact on uptake. Another possible explanation is that these cellular uptake experiments were conducted in the presence of serum, unlike earlier studies on disulfide-modified nanoparticles.^33–35^ Serum proteins, such as apolipoproteins,^69,70^ may adsorb to the surface of LNPs, predominating over CDL modifications in determining cellular uptake dynamics.

The data thus far indicate that cellular uptake alone cannot account for the increased functional mRNA delivery observed with the incorporation of CDLs. To further explore the mechanism, we evaluated the intracellular localization of LNPs, as endosomal/lysosomal escape is a critical barrier to efficient nucleic acid delivery. Lysosomal colocalization studies indicate that CDLs facilitate endosomal/lysosomal escape (Fig. 5 and S4†). We also evaluated membrane fusion activity using a hemolysis assay (Fig. S5†). Under serum-free conditions, LNPs lacking CDL (SM102) exhibited significantly higher membrane fusion activity than CDL-containing LNPs (SM102/CDL), particularly at acidic pH (6.5–4.5). These results suggest that, in the absence of serum, membrane fusion is primarily driven by electrostatic interactions between ionizable lipids and target membranes. In contrast, under 10% serum conditions, the overall hemolytic activity of all LNPs was markedly reduced. Nevertheless, SM102/CDL demonstrated higher fusion activity than the control LNPs at pH 7.5 and 6.5. These findings indicate that in the presence of serum, electrostatic interactions are likely attenuated—probably due to the formation of a protein corona—and that thiol-disulfide exchange between the cyclic disulfide moieties of CDL and membrane proteins may play a key role in promoting membrane fusion. Given that membrane-to-membrane proximity represents a critical rate-limiting step in the fusion process, CDL-mediated membrane association may substantially enhance endosomal escape. This finding aligns with a previous study reporting that disulfide bonds enhance the endosomal escape of polymeric nanoparticles *via* interactions with cellular exofacial thiols.^35^ Another factor contributing to the increased transfection activity associated with disulfide structures is the enhanced release of payloads into the cytoplasm.^18,38,71^ Once in the cytoplasm, mRNA must be released from the delivery carrier to enable translation. Disulfide bonds, cleaved in response to the reductive cytoplasmic environment, may promote the spontaneous release of payloads from LNPs. While this study did not directly examine cytoplasmic release, the more diffuse fluorescence of DiD in CDL-containing formulations (Fig. 5 and S4†) suggests that LNP disruption and subsequent nucleic acid release may also contribute to enhanced delivery.

We next evaluated whether CDLs could enhance functional mRNA delivery *in vivo*. CDL-incorporated LNPs demonstrated approximately 5-fold higher functional mRNA delivery compared to LNPs without CDLs in a mouse model (Fig. 6A and B). This improvement was accompanied by robust antigen-specific immune responses against tumors through the delivery of antigen-encoded mRNA (Fig. 6C–F and S6†). In addition, a single-dose toxicity study revealed no significant body weight loss or cytokine induction (IL-6, TNFα, IFNβ) following subcutaneous administration of LNP-mRNA in mice, regardless of CDL incorporation, indicating that the CDL strategy does not compromise *in vivo* tolerability (Fig. S8†). However, real-world data on mRNA vaccines have demonstrated that LNP formulations can exhibit certain reactogenicity profiles, including adverse effects.^72^ LNPs used in mRNA vaccines are known to induce inflammatory responses such as injection site inflammation, hepatic accumulation, and biodistribution to multiple tissues, including the brain, in both humans and animal models.^73^ Ionizable lipids, in particular, are recognized for their immunostimulatory potential. Studies have shown that empty LNPs containing ionizable cationic lipids elicit higher levels of pro-inflammatory cytokines, including GM-CSF, IL-5, and IL-6, compared to LNPs lacking such lipids.^74^ Moreover, the innate and adaptive immune responses triggered by LNPs have been linked to the chemical structure of ionizable lipids. Specifically, amine headgroups have been shown to promote immunogenicity through interactions with toll-like receptor 4 (TLR4) and CD1d, as well as through the induction of lipid raft formation.^75^ Although the onset of toxicity depends on the dose, the structural modification introduced by CDL may alter immune interactions due to changes in ionizable lipid architecture. Furthermore, disulfide-based systems present potential toxicity concerns, including disruption of cellular redox homeostasis and protein S-alkylation. Disulfide cleavage in the reductive intracellular environment produces thiol groups that can deplete intracellular glutathione (GSH), potentially inducing oxidative stress and triggering apoptosis or necrosis at higher concentrations. Additionally, these thiols may react with cysteine residues on proteins, potentially interfering with enzymatic activity or cellular signaling pathways. Nonetheless, prior studies have reported that disulfide-containing lipid molecules exhibit low cytotoxicity in both *in vitro* and *in vivo* settings,^37,71,76,77^ supporting the notion that disulfide incorporation is biocompatible and safe at clinically relevant doses. While further investigation into immunogenicity and inflammatory responses at higher dosing levels is warranted, the overall toxicity of CDL-containing formulations does not appear to exceed that of existing lipid-based systems. While further optimization is needed to enhance activity for clinical applications, we propose that the disulfide-based strategy holds significant promise for improving LNP-mediated mRNA delivery and advancing next-generation mRNA delivery systems.

It is important to acknowledge the limitations of this study. First, we tested only one cyclic disulfide structure derived from α-lipoic acid. Future studies should explore additional chemical structures to enhance activity and clarify structure–activity relationships, such as variations in the ring size, carbon chain length from the tertiary amine, linker type, and the introduction of branched or multivalent structures. Second, our study focused on two ionizable lipids (MC3 and SM102), one sterol lipid (Chol), one phospholipid (DOPE), and one PEG-lipid (DMG-PEG). The LNP field has seen the development of diverse phospholipids,^16,47^ sterol lipids,^22,78,79^ PEG lipids,^80^ and PEG alternatives,^81^ as well as ionizable lipids. Future work should include a broader range of helper lipids and PEG/PEG alternatives, as well as experiments with varying molar ratios. Third, our *in vivo* experiments were limited to subcutaneous (SC) administration in mice. Most cell types express surface thiols that are involved in cell signaling and can facilitate the uptake of exogenous materials.^82–85^ Disulfide groups, including those in CDL, are reactive with thiols on serum proteins and cell surfaces; therefore, the efficiency of delivery may vary depending on the route of administration, which influences the frequency, localization, and extent of these thiol-disulfide exchange reactions. SC administration forms a local depot at the injection site, allowing gradual release into the systemic circulation. This route provides several advantages for the CDL strategy. First, the depot formation limits immediate exposure to the systemic reductive environment, thereby helping preserve the cyclic disulfide structure prior to cellular uptake. Second, the relatively slow absorption through lymphatic and vascular systems enables prolonged interaction with local immune and stromal cells, potentially enhancing therapeutic efficacy. In contrast, intravenous or intramuscular routes—which were not evaluated in this study—may expose CDL-containing LNPs to a more immediate and widespread reductive environment, increasing the likelihood of premature disulfide cleavage. Although SC administration was chosen to minimize early degradation and optimize delivery efficiency under these considerations, further investigation is warranted to evaluate the biodistribution, pharmacokinetics, and functional mRNA delivery of CDL-based LNPs administered *via* other clinically relevant routes. Despite these limitations, our data provide a evidence for the applicability of the disulfide strategy in *in vivo* LNP systems to improve mRNA delivery efficiency. These findings could contribute to the advancement of mRNA-based drug development and therapeutic innovation.

## Methods

### Materials

1,2-Dioleoyl-*sn-glycero*-3-phosphoethanolamine (DOPE) and 1,2-dimirystoyl-*rac-glycero* methoxyethylene glycol 2000 ether (DMG-PEG2k) were purchased from NOF CORPORATION (Tokyo, Japan). Cholesterol (Chol), and methyl-β-cyclodextrin (MβCD) were purchased from SIGMA Aldrich (St. Louis, MO, USA). DLin-MC3-DMA and SM102 were purchased from Selleck Biotech (Houston, TX, USA). 1,1′-Dioctadecyl-3,3,3′,3′-tetramethylindodicarbocyanine (DiD) was purchased from Invitrogen (Carlsbad, CA). Sodium azide was purchased from KISHIDA CHEMICAL Co Ltd. LTD (Osaka, Japan). Amiloride hydrochloride and chlorpromazine hydrochloride were purchased from the Tokyo Chemical Industry Co. Ltd. (Tokyo, Japan). Nano-Glo® Luciferase Assay System was obtained from Promega (Madison, USA). Dulbecco's modified Eagle's medium (DMEM) and fetal bovine serum (FBS) were purchased from Sigma-Aldrich Co. (St. Louis, MO). All other materials were of reagent grade and commercially available.

### Animals

4-Week-old female ICR mice and 4-week-old female C57BL6N mice were purchased from Japan SLC. Mice were bred using ROBORACK automated laboratory rearing system (G-LINX CO., LTD, Japan). The experimental protocols used in this study were reviewed and approved by the Nagoya University Animal Care Committee, in accordance with the “Guide for the Care and Use of Laboratory Animals”. In all experiments, animals were used without fasting.

### LNP preparation

mRNA-loaded LNPs were prepared using the ethanol dilution method modified from previous studies.^4,48^ Briefly, 10 μg mRNA was dissolved in 200 μL of 5 mM citrate buffer (pH 4). Lipids were dissolved in 200 μL of ethanol with the following lipid composition: ionizable cationic lipid/DOPE/Chol/DMG-PEG2k = 50/10/38.5/1.5 molar ratio, total lipid, 400 nmol for 10 μg of mRNA. Under vortex mixing, the lipid ethanol solution was added to citrate buffer containing mRNA, and then 1 mL of citrate buffer was added to dilute the sample. The LNP suspension was centrifuged at 1500 × *g* for 30 min at 25 °C using an Amicon Ultra 100k. LNPs were recovered in PBS (pH 7.4).

### Characterization of mRNA-loaded LNPs

Zeta average size, polydispersity index (PDI), and zeta potential of the LNPs were measured using a Zetasizer Pro (Malvern, UK). The final mRNA concentration and encapsulation efficiency (EE) were determined using a RiboGreen assay kit (Molecular Probes, Eugene, OR, USA) following the manufacturer's protocol. LNPs were diluted in TE buffer (10 mM Tris-HCl, 1 mM EDTA, pH 7.5) containing RiboGreen reagent in the presence or absence of 1% (w/v) Triton X-100. Fluorescence intensity was measured using a Tecan Spark plate reader at an excitation wavelength (*λ*_ex_) of 480 nm and an emission wavelength (*λ*_em_) of 520 nm. RNA concentrations were calculated using RNA standard curves. RNA encapsulation efficiency was determined by comparing the RNA concentrations in the presence and absence of Triton X-100. To measure the apparent p*K*_a_ of the LNP surface, a 6-(*p*-toluidino)-2-naphthalenesulfonic acid (TNS) assay was performed. LNPs were diluted with saline to a 0.5 mM lipid concentration. A 12 μL aliquot of the diluted LNPs was mixed with 188 μL of TNS assay buffer (20 mM citrate buffer (pH 3.5–5.5), 20 mM phosphate buffer (pH 6.0–8.0), or 20 mM Tris-HCl buffer (pH 8.5–9.0) containing 130 mM NaCl and 6.7 μM TNS) in each well of a 96-well black plate. TNS fluorescence (Ex/Em = 321/447 nm) was measured using a Tecan plate reader. The pH and TNS fluorescence values were plotted and the pH at which 50% of the lipid was charged was used as the apparent p*K*_a_.

### mRNA synthesis

Nanoluciferase (NLuc) mRNA was synthesized and purified as previously described.^86^ Briefly, *in vitro* transcription of NLuc mRNA was performed using an in-house prepared T7 RNA polymerase and an in-house developed cap analog, DiPureCap. DiPureCap contains a hydrophobic photocaging group linked at its 7′-methylguanosine that isolates capped RNA species from uncapped ones using different elution times in reversed-phase high-performance liquid chromatography (RP-HPLC). After *in vitro* transcription, the mRNA was recovered by lithium chloride precipitation. mRNA containing DiPureCap was then purified by RP-HPLC using a YMC-TriartBio C4 column (250 × 4.6 mm I.D., S-5 μm, 12 nm) on a Shimadzu Prominence HPLC system (pump, LC-20AD; detector SPD-M40) with solution A (50 mM triethylammonium acetate (TEAA, pH 7.0) containing 5% acetonitrile) and solution B (acetonitrile) at a flow rate of 1 mL min^−1^. The content of solution B was increased from 0 to 20% over 20 min. The column temperature was maintained at 50 °C. Purified RNA was recovered by alcohol precipitation. To remove the hydrophobic photocaging group of DiPureCap, the RNA sample was subjected to 365 nm light irradiation at 4 mW cm^−2^ for 10 min at room temperature using a MAX-350 compact xenon light source (Asahi Spectra). After light irradiation, capped mRNA was purified by RP-HPLC under the same conditions as described above to remove residual dsRNA contamination. Purified RNA was recovered by alcohol precipitation and dissolved in deionized water. For the preparation of ovalbumin (OVA) mRNA, the IVT reaction was performed under the same conditions except that CleanCap Reagent AG – (TriLink)^87^ was used instead of DiPureCap and N1-methylpseudouridine was used instead of uridine. The mRNA was purified using the Monarch RNA Cleanup kit (New England Biolabs).

### Cell culture and *in vitro* transfection assay

HeLa cells were cultured in DMEM supplemented with 10% heat-inactivated FBS, penicillin (100 U mL^−1^), and streptomycin (100 μg mL^−1^) at 37 °C in an atmosphere containing 5% CO_2_ and 95% humidity. HeLa cells were seeded into a 96-well plate at 6000 cells per well 1 d before LNP addition. LNPs were added to each well at any mRNA concentration, followed by incubation at 37 °C and 5% CO_2_ for 24 h. Cells were washed with PBS, and 100 μL of CCK-8 solution diluted 10-fold with the medium was added to each well. Absorbance was measured using a plate reader (Abs 450 nm) at 30 min after incubation. Cell viability was determined by calculating the value when the untreated sample was set at 100% (WST8 assay). After the WST8 assay, the cells were washed with PBS, and 50 μL of PBS was added to each well. NanoGlo-substrate (Promega) and lysis buffer were mixed at a volume ratio of 1 : 50 and 50 μL of the solution was added to each well. 2 minutes after shaking, the luminescence was measured using a plate reader. NLuc expression was expressed as relative light units (RLU).

### Cellular uptake study

24 h before the experiments, HeLa cells were cultured in a 12-well plate at 100 000 cells per well. Different LNPs were prepared as described above, where a DiD label was mixed with lipids (0.5 mol% of total lipids). DiD-labeled LNPs were added to the cells, which were then incubated for 6 h at 37 °C. The medium was then removed, and the cells were washed with PBS and trypsinized. The cell suspension was centrifuged (500 × *g*, 4 °C, 5 min), and the precipitated cells were suspended in 1 ml of FACS buffer (0.5% bovine serum albumin, 0.2% NaN_3_, and 2 mM EDTA in PBS). Centrifugation and resuspension in FACS buffer were repeated twice before the final cell suspension was passed through a nylon mesh to remove cell aggregates. DiD fluorescence of the cells was analyzed using a CytoFLEX flow cytometer.

### Observation of lysosomal localization of LNPs

24 h before adding DiD-labeled LNPs, HeLa cells were seeded in a 96-well plate at 10 000 cells per well (for fluorescence microscopy observation, Keyence BZ-X800) or in a 35 mm glass-bottom dish at 200 000 cells per dish (for confocal microscopy imaging, Mica). DiD-labeled LNPs were added into each well, and the nucleus and lysosomes were stained with Hoechst 33342 (Wako, Japan) and Lysosensor Green DND-189 (Thermo, USA), respectively, 4 h after LNP addition. For semi-quantitative analysis, the percentage of lysosomal colocalization (% colocalization) was calculated using the following formula: % colocalization = area of colocalization (yellow area showing colocalization of lysosomes and LNP)/area of LNP (red area showing LNP localization). The average of the values calculated from 6–10 spot images per well was calculated. In other words, the colocalization rate was calculated from 18–30 images per sample. The lower the percentage of, the higher the rate of escape from the lysosomes.

### *In vivo* NLuc-mRNA delivery

LNPs were subcutaneously injected to 4–6-week-old female ICR mice at 4 μg of mRNA dose per mouse. Mice were anesthetized with isoflurane and then 150 μl of the fluorofurimazine (FFz) substrate solution (2.4 mM) was intraperitoneally injected into each mouse (0.48 μmol FFz per mouse) at 6 h after LNP administration. Ten minutes after the substrate injection, the mice were imaged using an IVIS imaging system (IVIS Lumina LT, PerkinElmer). The luminescent intensity was quantified using Living Image 4.0.

### Antigen-specific antibody production

5–6-Week-old-C57BL/6N-female mice were subcutaneously administered with PBS, naked OVA-encoding mRNA, or LNP encapsulating OVA-encoding mRNA (on day 0 and 7, 4 μg mRNA per mouse). At 7 days after the second administration, blood was collected and the level of OVA-specific IgG1 in the serum was measured using LBIS™ Mouse OVA-IgG1 ELISA kit (FUJIFILM, Japan).

### Induction of antigen-specific CD8 positive cells

5–6-Week-old-C57BL/6N-female mice were subcutaneously administered with PBS, naked OVA-encoding mRNA, or LNP encapsulating OVA-encoding mRNA (on day 0 and 7, 4 μg mRNA per mouse). 14 days after the second administration, the spleen was collected and splenocytes were isolated based on previous publications.^4,48^ Briefly, single splenocytes were prepared by tamping the spleen with the rubber end of a plunger from a 2.5 ml syringe in 5 ml of RPMI1640 supplemented with 10% heat-inactivated FBS, penicillin (100 U ml^−1^), streptomycin (100 μg ml^−1^), 10 mM HEPES, 100 mM sodium pyruvate and 50 nM 2-mercaptoethanol (Gibco). The cell suspension was passed through a nylon mesh to remove any cell aggregates. After centrifugation at 500 g for 5 minutes at 4 °C, the supernatant was removed and ACK lysing buffer was added to the cell precipitate, followed by incubation at room temperature for 5 minutes. The centrifugation and re-suspension in RPMI were repeated three times. The final suspension was passed through a nylon mesh. Cells were suspended in FACS buffer and stained using following reagents; anti-CD16/32 antibody (Proteintech 65057-1-IG), T-Select H-2Kb OVA Tetramer-SIINFEKL-APC (MBL, TS-5001-2C), and anti-CD8 (Mouse) mAb-PE (MBL, K0227-5). A 10 μl portion of 7-AAD (Biolegend) was added to 1 ml of each sample before the analysis to detect dead cells. The resulting stained cells were analyzed by flow cytometry (CytoFLEX) to evaluate OVA-specific CD8 positive populations. A representative gating strategy was shown in Fig. S7.†

### Anti-tumor effect

5-Week-old C57BL/6N mice were subcutaneously injected with LNPs encapsulating the mRNA at day 0 and 7 (each injection dose; 4 μg mRNA per mouse). At 7 days after the second administration, 5 × 10^5^ antigen (OVA)-expressing E.G7-OVA lymphoma cells were subcutaneously injected. Tumor volume was calculated by the following formula: (major axis × minor axis2) × 0.52. The study was conducted with a set period of tumor growth monitoring: 30 days. Mice were sacrificed if any signs of suffering appeared before the time limit was reached.

### Statistical analysis

Multiple treatments were compared using one-way analysis of variance (ANOVA) followed by the Tukey–Kramer or Dunnett test. Pairwise comparisons between treatments were performed using a two-tailed Student's *t*-test. Statistical significance was set at *P* < 0.05. All statistical analyses were conducted with R software version 4.3.1 using “RcmdrPlugin.EZR” packages.^88^

## Author contributions

Conceptualization, S. K. and H. A.; methodology, S. K., K. O. and H. A.; validation, S. K. and H. A.; formal analysis, S. K.; investigation, K. O., S. T., S. K., and N. A.; resources, Y. K., F. H., M. I., and H. A.; data curation, K. O., S. T., and S. K.; writing – original draft preparation, K. O., S. K., and S. T.; writing – review and editing, K. O., S. K., S. T., Y. K., F. H., M. I., N. A., and H. A.; visualization, S. K.; supervision, S. K., and H. A.; project administration, S. K., and H. A.; funding acquisition, H. A. All authors have read and agreed to the published version of the manuscript.

## Conflicts of interest

S. Kimura, Y. Kimura, N. Abe and H. Abe have filed intellectual property patents related to this publication. The authors declare no other competing financial interest.

## Supplementary Material

MD-OLF-D5MD00084J-s001

## Data Availability

All relevant data supporting the findings of this study are included within the manuscript and its ESI.†
