# Supplementary material for: In vivo demonstration of enhanced mRNA delivery by cyclic disulfide-containing lipid nanoparticles for facilitating endosomal escape
Source: RSC Med Chem. 2025 Jun 27;16(9):4122–37. doi: 10.1039/d5md00084j (PMC12257974; doi:10.1039/d5md00084j)

Supplementary Information (SI) for RSC Medicinal Chemistry.
This journal is © The Royal Society of Chemistry 2025

SUPPORTING INFORMATION

In Vivo Demonstration of Enhanced mRNA Delivery by Cyclic Disulfide-Containing Lipid Nanoparticles for Facilitating Endosomal Escape

Seigo Kimura [1*], Kana Okada [2], Noriaki Matsubara [2], Fangjie Lyu [2], Susumu Tsutsumi [2], Yasuaki Kimura [2], Fumitaka Hashiya [2], Masahito Inagaki [2], Naoko Abe [2], and Hiroshi Abe [2,3]*

[1] Integrated Research Consortium on Chemical Sciences (IRCCS), Nagoya University, Nagoya, Aichi 464-8602, Japan

[2] Department of Chemistry, Graduate School of Science, Nagoya University, Nagoya, Aichi 464-8602, Japan

[3] Institute for Glyco-core Research (iGCORE), Nagoya University, Nagoya, Aichi 464-8601, Japan

*Correspondence: S.K.; kimura.seigo.v9@f.mail.nagoya-u.ac.jp, H.A.; h-abe@chem.nagoya-u.ac.jp

Supplementary Methods: synthesis of CDLs

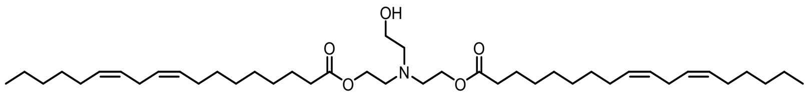

C18-2-A'

Thionyl chloride (30 µL, 0.42 mmol, 1.2 eq) was dissolved in DCM 2 mL, and the solution was stirred in the ice bath. Linoleic acid (110 µL, 0.35 mmol, 1.0 eq) was dissolved in DCM 1.0 mL and added to the mixture by dropwise. Stirred for 3 hours, confirmed the reaction was completed by TLC. The mixture was evaporated completely.

The starting material was dissolved in DCM 2 mL. Triethylamine 48 µL (0.35 mmol, 1.0 eq) and triethanolamine 23 µL (0.18 mmol, 0.5 eq) were added to the solution, then stirred at room temperature overnight. After confirmed the completion of the reaction by TLC, added MQ 5mL and extract with DCM. The organic phase was washed with brine twice and Na_2SO_4 dried, evaporated. Purified by column chromatography (DCM/MeOH=19/1~12/1). Target compound was obtained (24 mg, yield 20%).

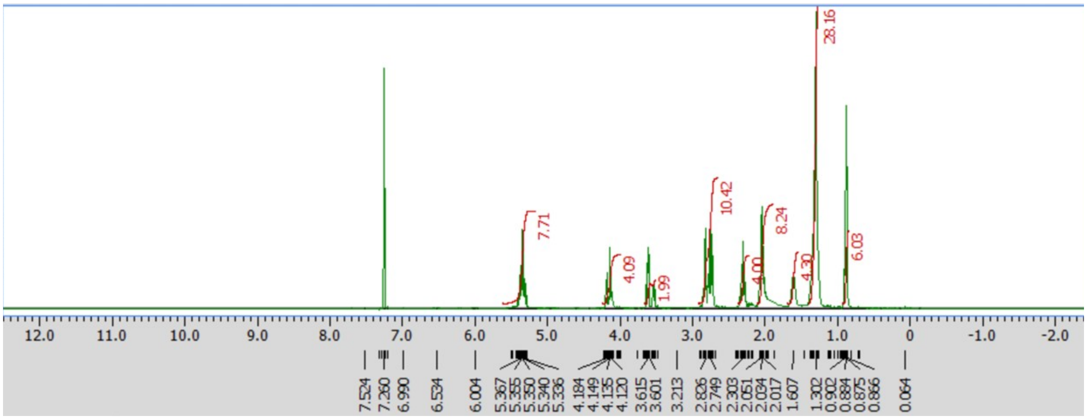

^{1}H-NMR (400 MHz, $CDCl_3$)

δ: 0.866-0.902 (6H, t, J = 6.8 Hz), 1.280-1.302 (28H, m), 1.607 (4H, s), 2.017-2.07 (8H, q), 2.28-2.34 (4H, m), 2.72-2.84 (10H, m), 3.60-3.63 (2H, t, J = 4.0 Hz), 4.12-4.20 (4H, m), 5.30-5.38 (8H, m) ppm.

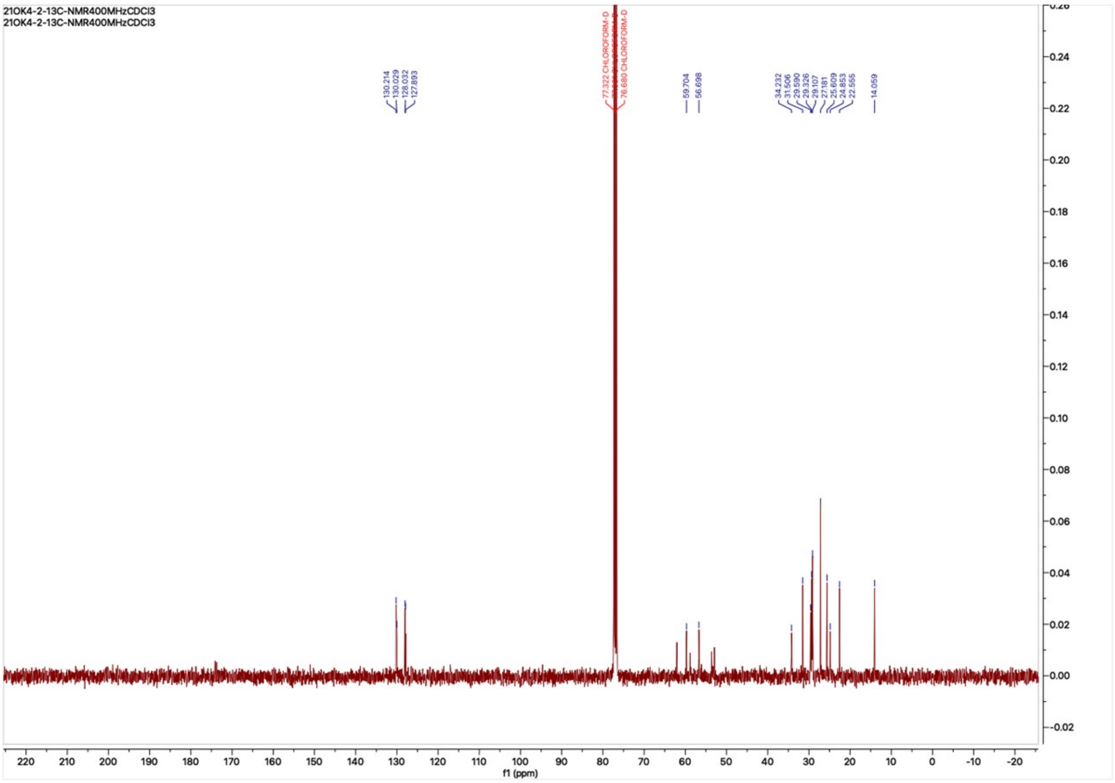

^{13}C-NMR (400 MHz CDCl$_3$)

δ: 14.059, 22.555, 24.853, 25.609, 27.181, 29.107, 29.326, 29.590, 31.506, 34.232, 56.698, 59.704, 127.893, 128.032, 130.029, 130.214 ppm.

HRMS(ESI) calcd. for $C_{42}H_{75}NO_5$ $[M+H]^+$ 674.5645 found 674.5725.

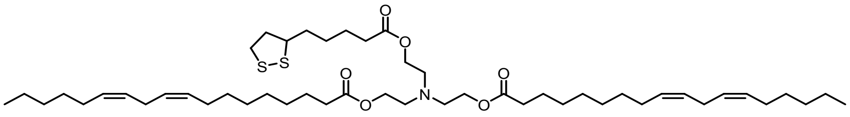

C18-2-A

C18-2-A'(100 mg, 0.14 mmol, 1.0 eq) was dissolved in DCM 10 mL. DMAP (17 mg, 0.14 mmol, 1.0 eq) and lipoic acid (29 mg, 0.14 mmol, 1.0eq) was added to the solution. Stirred in the ice bath, then DCC (29 mg, 0.14 mmol, 1.0 eq) dissolved in DCM 1,0 mL was added by dropwise. The reaction mixture was stirred at room temperature overnight. After confirming the complete of the reaction by TLC, the reaction mixture was filtered and concentrated by evaporation. The crude was purified by column chromatography (Hexane/ EtOAc =5/1) to obtain the target compound (72 mg, yield 60%).

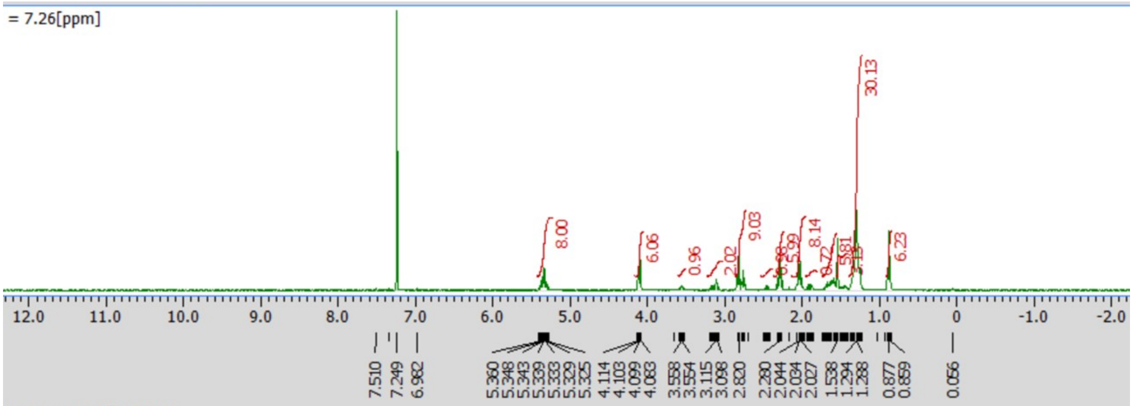

^{1}H-NMR (400 MHz $CDCl_3$)

δ: 0.899-0.894 (6H, m), 1.247-1.271 (30H, m), 1.288-1.294 (3H,m), 1.577-1.660 (6H, m), 1.872-1.939 (1H, q, J = 6.8 Hz), 2.009-2.061 (8H, m), 2.261-2.306 (6H, m), 2.412-2.491 (1H, m), 2.741-2.836 (9H, m), 3.070-3.201 (2H, m), 3.52 2-3.590 (1H, m), 4.083-4.136 (6H, m), 5.288-5.390 (8H, m) ppm.

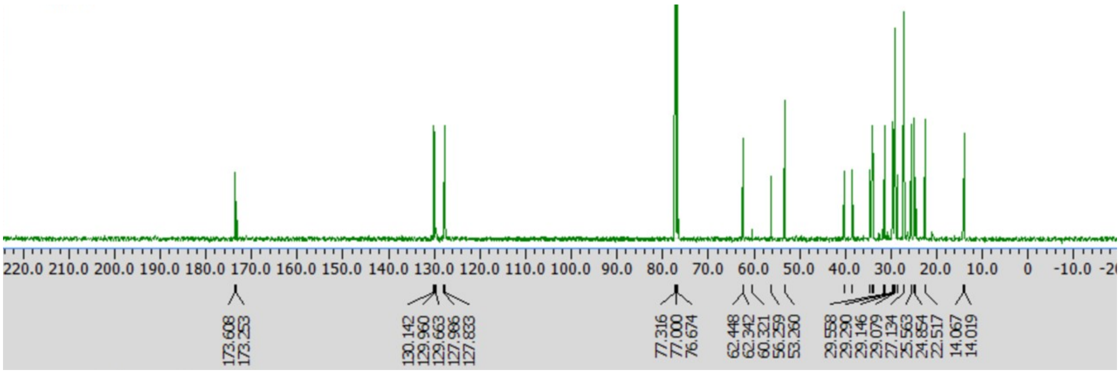

^{13}C-NMR (400 MHz $CDCl_3$)

δ: 14.019, 14.067, 22.517, 24.854, 25.563, 27.134, 29.079, 29.146, 29.290, 29.558, 53.260, 56.259, 60.321, 62.342, 62.448, 127.833, 127.986, 129.663, 129.960, 130.142, 173.253, 173.608 ppm.

HRMS(ESI) calcd. for $C_{50}H_{87}NO_6S_2$ $[M+Na]^+$ 884.5872 found 884.6091.

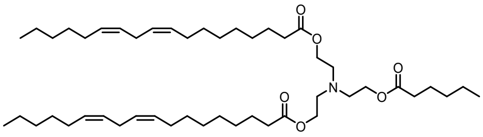

Control lipid

C18-2-A’ (0.2 g, 0.28 mmol, 1.0 eq) was dissolved in DCM 10 mL. DMAP (34 mg, 0.28 mmol, 1.0 eq) and Hexanoic acid (32 mg, 0.28 mmol, 1.0eq) was added to the solution. Stirred in the ice bath, then DCC (58 mg, 0.28 mmol, 1.0 eq) dissolved in DCM 1,0 mL was added by dropwise. The reaction mixture was stirred at room temperature overnight. After confirming the complete of the reaction by

TLC, the reaction mixture was filtered and concentrated by evaporation. The crude was purified by column chromatography (Hexane/ EtOAc =5/1) to obtain the target compound (0.15 g, yield 70%).

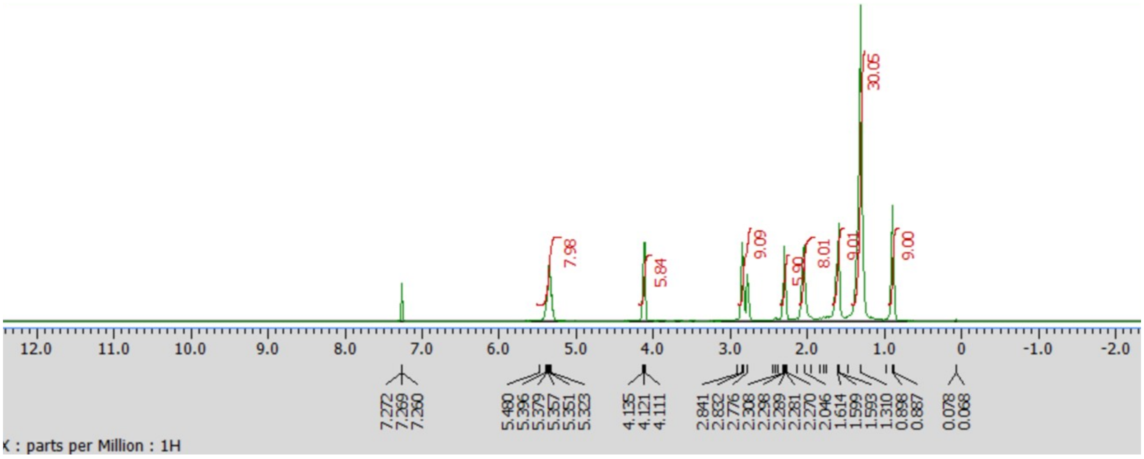

^{1}H-NMR (400 MHz $CDCl_3$)

δ: 0.887-0.898 (9H, d), 1.310-1.480 (30H, m), 1.593-1.614 (9H, t, J = 6.0 Hz), 1.948-2.046 (8H, d), 2.281-2.308 (6H, m), 2.776-2.915 (9H, m), 4.111-4.135 (6H, t, J = 4.0 Hz), 5.323-5.396 (8H, m) ppm.

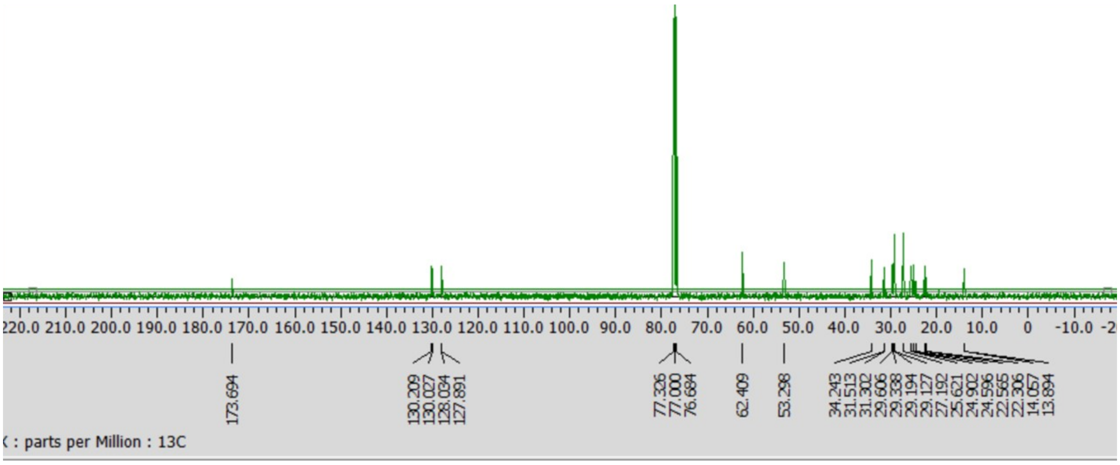

^{13}C-NMR (400 MHz $CDCl_3$)

δ: 13.894, 14.057, 22.306, 22.565, 24.596, 24.902, 25.621, 27.192, 29.127, 29.194, 29.338, 29.606, 31.302, 31.513, 34.243, 53.298, 62.409, 127.891, 128.034, 130.027, 130.209, 173.694 ppm.

HRMS(ESI) calcd. for $C_{48}H_{85}NO_6$ $[M+Na]^+$ 772.6385 found 772.6707.

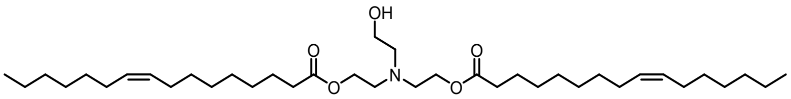

C16-1-A'

Palmitoleic acid (0.10 g, 0.39 mmol, 2.0 eq) was dissolved in DCM 4 mL. Triethanolamine (58 mg, 0.20 mmol, 1.0 eq) and DMAP (47 mg, 0.39 mmol, 2.0 eq) were added. Then stirred at 0℃. DCC (80 mg, 0.39 mmol, 2.0 eq) was dissolved in DCM 2mL and added to the solution by dropwise. After checking the completion of the reaction by TLC, residue was removed by filtering. Then evaporate and purified by column chromatography (DCM/MeOH=15/1). The target material (43 mg, yield 35%) was obtained.

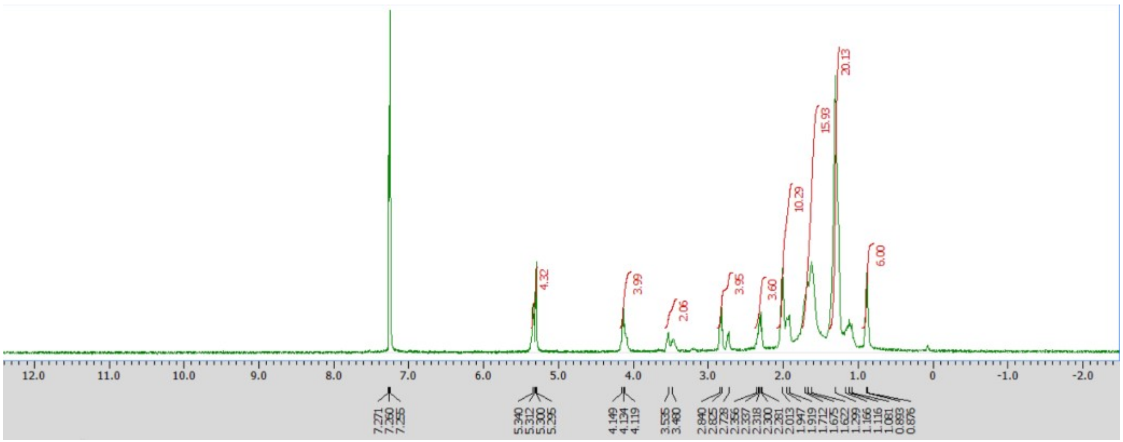

^{1}H-NMR (400 MHz $CDCl_3$)

δ: 0.876-0.893 (6H, m), 1.116-1.299 (20H, m), 1.62-1.71 (16H, m), 1.91-2.01 (10H, m), 2.28-2.34 (4H, m), 2.72-2.84 (4H, m), 3.48-3.53 (2H, m), 4.12-4.15 (4H, m), 5.30-5.34 (4H, q) ppm.

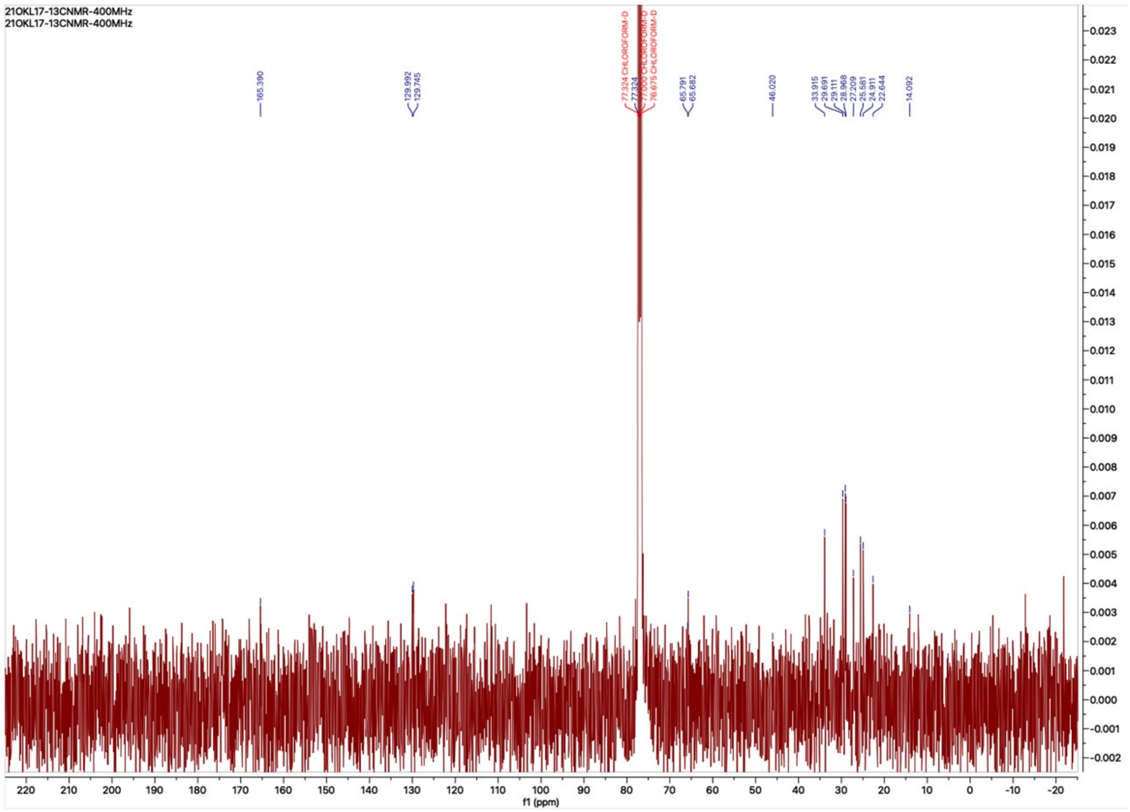

^{13}C-NMR (400 MHz $CDCl_3$)

δ: 14.092, 22.644, 24.911, 25.581, 27.209, 28.968, 29.111, 29.691, 33.915, 46.020, 65.682, 65.791, 129.745, 129.992, 165.390 ppm.

HRMS(ESI) calcd. for $C_{38}H_{71}NO_5$ $[M+H]^+$ 621.5322 found 621.5320.

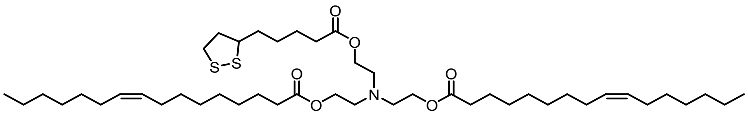

C16-1-A

C16-1-A' (43 mg, 0.07 mmol, 1.0 eq) was dissolved in DCM 2 mL. a-Lipoic acid (25 mg, 0.12 mmol, 1.5 eq) and DMAP (15 mg, 0.12 mmol, 1.5 eq) were added. Then stirred at 0°C. DCC (25 mg, 0.12 mmol, 1.5 eq) was dissolved in DCM 1 mL and added to the solution by dropwise. Stirred in ice bath for 3 hours, at r.t overnight. After checking the completion of the reaction by TLC, residue was removed by filtering. Then evaporate and purified by column chromatography (DCM/MeOH=15/1). The target material (0.12 g, yield 98 %) was obtained.

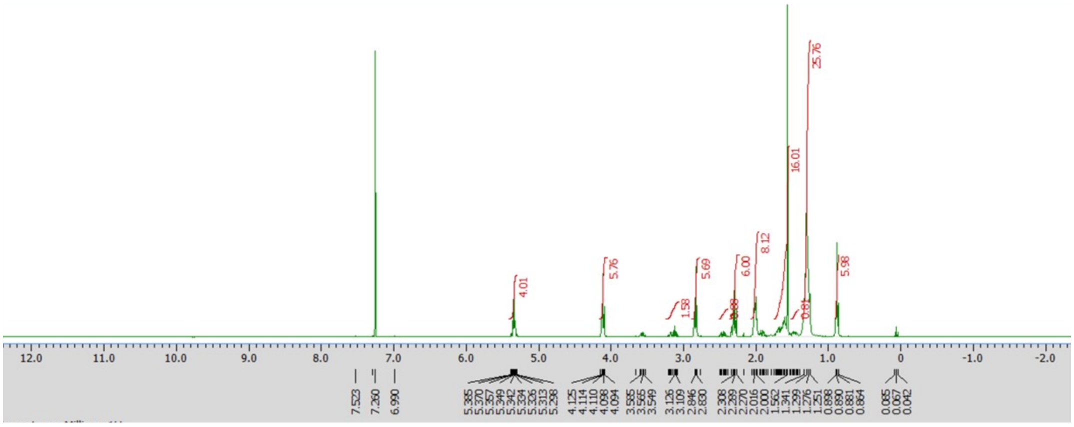

^{1}H-NMR (400 MHz $CDCl_3$)

δ: 0.86-0.90 (6H, m), 1.25-1.34 (26H, m), 1.42-1.51 (1H, m), 1.53-1.75 (16H, m), 1.98-2.06 (8H, m), 2.27-2.34 (6H, m), 2.41-2.50 (1H, m), 2.82-2.85 (6H, t, J = 6.0 Hz), 2.82-2.85 (6H, t, J = 6.0 Hz), 3.08-3.17 (2H, m), 4.09-4.15 (6H, m), 5.30-5.39 (4H, m) ppm.

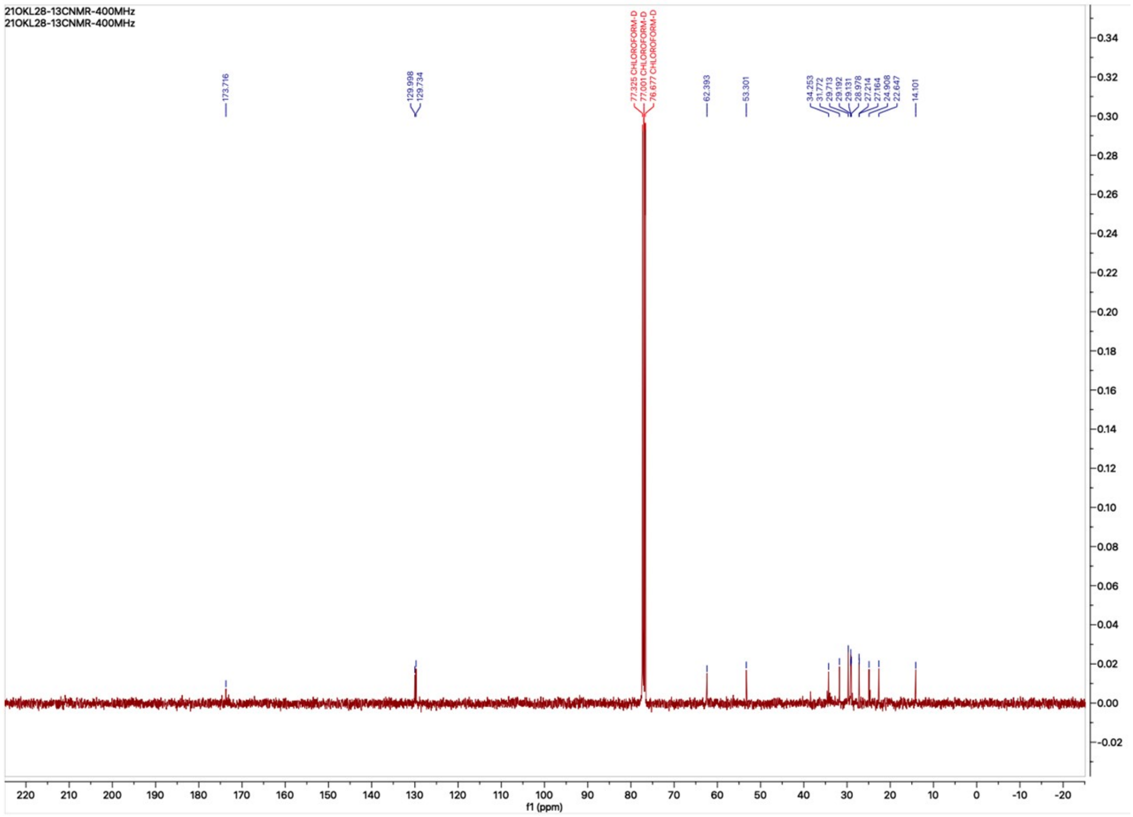

^{13}C-NMR (400 MHz $CDCl_3$)

δ: 14.101, 22.647, 24.721, 24.908, 27.164, 27.214, 28.978, 29.131, 29.192, 29.289, 29.713, 31.772, 34.253, 34.344, 34.697,38.569, 53.301, 62.393, 62.604, 129.734, 129.998, 173.716 ppm.

HRMS(ESI) calcd. for $C_{46}H_{83}NO_6S_2$ $[M+H]^+$ 810.5742 found 810.5752.

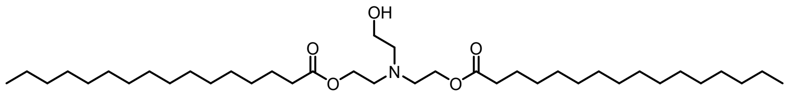

C16-0-A'

Stearic acid (0.76g, 2.68 mmol, 2.0 eq) was dissolved in DCM 18 mL. Triethanolamine (0.20 g, 1.34 mmol, 1.0 eq) and DMAP (0.33 g, 2.68 mmol, 2.0 eq) were added. Then stirred at 0 °C. DCC (0.55 g, 2.68 mmol, 2.0 eq) was dissolved in DCM 6 mL and added to the solution by dropwise. After checking the completion of the reaction by TLC, residue was removed by filtering. Then evaporate and purified by column chromatography (DCM/MeOH=15/1). The target material (0.59 g, yield 65%) was obtained.

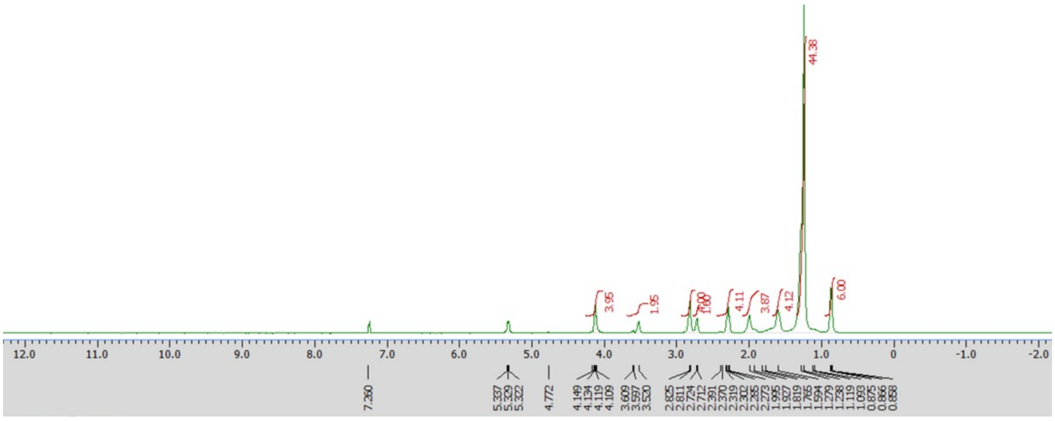

^{1}H-NMR (400 MHz $CDCl_3$)

δ: 0.87-0.90 (6H, t, J = 6.8 Hz), 1.25-1.29 (46H, m), 1.61-1.78 (6H, m), 1.94-2.01 (4H, m), 2.29-2.34 (4H, m), 2.73-2.84 (6H, m), 3.54-3.63 (2H, m), 4.13-4.18 (4H, m) ppm.

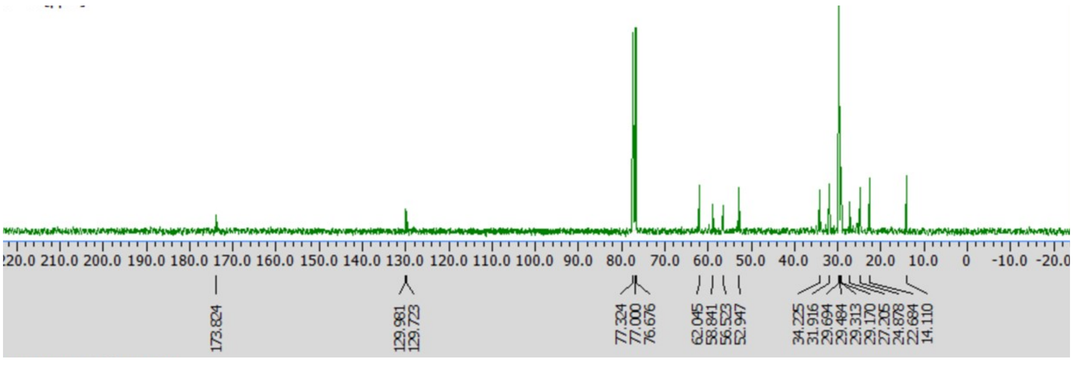

^{13}C-NMR (400 MHz $CDCl_3$)

δ: 14.110, 22.684, 24.878, 27.205, 29.170, 29.313, 29.5484, 29.694, 31.916, 34.225, 52.947, 56.523, 58.841, 62.045, 129.723, 129.981, 173.824 ppm.

HRMS(ESI) calcd. for $C_{38}H_{75}NO_5$ $[M+H]^+$ 626.5653 found 626.5677.

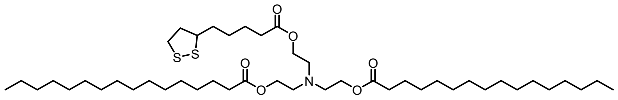

C16-0-A

C16-0-A’(0.30 g, 0.48 mmol, 1.0 eq) was dissolved in DCM 8 mL . a-Lipoic acid (0.12 g, 0.58 mmol, 1.2 eq) and DMAP (70 mg, 0.58 mmol, 1.2 eq) were added. Then stirred at 0℃. DCC (0.12 g, 0.58 mmol, 1.2 eq) was dissolved in DCM 4 mL and added to the solution by dropwise. After checking the completion of the reaction by TLC, residue was removed by filtering. Then evaporate and purified by column chromatography (DCM/MeOH=15/1). The target material (0.35 g, yield 89%) was obtained.

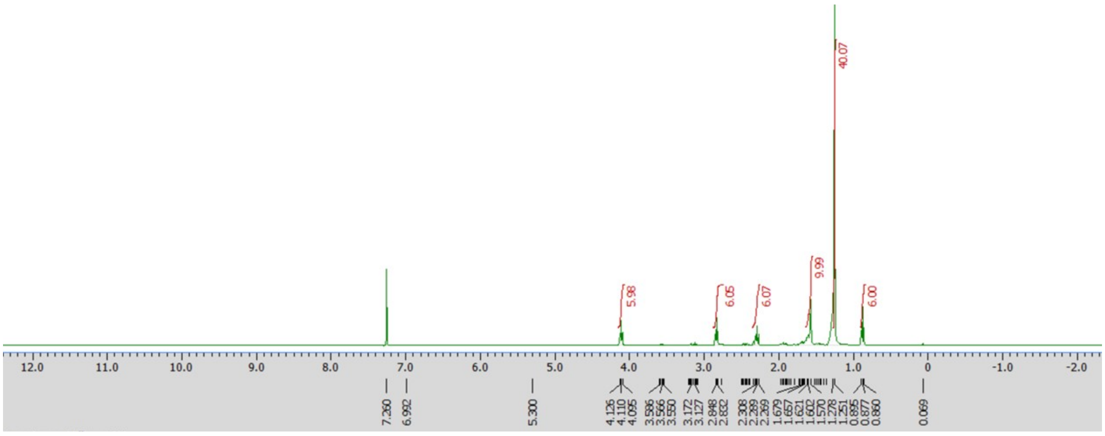

^{1}H-NMR (400 MHz $CDCl_3$)

δ: 0.86-0.90 (6H, t, J = 6.8 Hz), 1.25-1.29 (46H, m), 1.57-1.73 (16H, m), 1.83-1.97 (1H, m), 2.27-2.32 (6H, m), 2.82-2.85 (6H, t, J = 6.0 Hz), 4.10-4.14 (6H, t, J = 6.0 Hz) ppm.

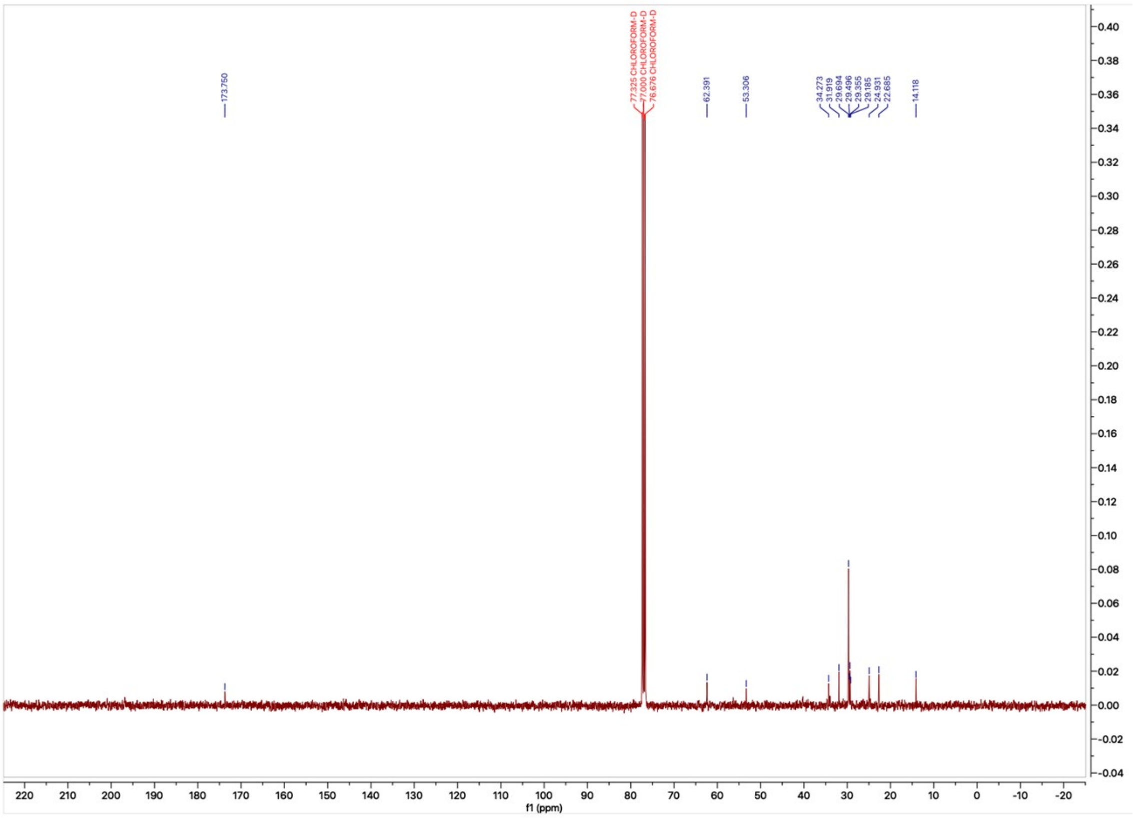

^{13}C-NMR (400 MHz CDCl$_3$)

δ: 14.118, 22.685, 24.931, 29.185, 29.355, 29.496, 29.694, 31.919, 34.273, 53.306, 62.391, 173.750 ppm.

HRMS(ESI) calcd. for $C_{46}H_{87}NO_6S_2$ $[M+Na]^+$ 836.5872 found 836.5860.

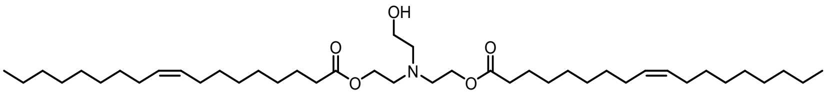

C18-1-A'

Oleic acid (0.76g, 2.68 mmol, 2.0 eq) was dissolved in DCM 17 mL. Triethanolamine (0.2 g, 1.34 mmol, 1.0 eq) and DMAP (0.33 g, 2.68 mmol, 2.0 eq) were added. Then stirred at 0℃. DCC (0.55 g, 2.68 mmol, 2.0 eq) was dissolved in DCM 6 mL and added to the solution by dropwise. After checking the completion of the reaction by TLC, residue was removed by filtering. Then evaporate and purified by column chromatography (DCM/MeOH=15/1). The target material (0.89 g, yield 96%) was obtained.

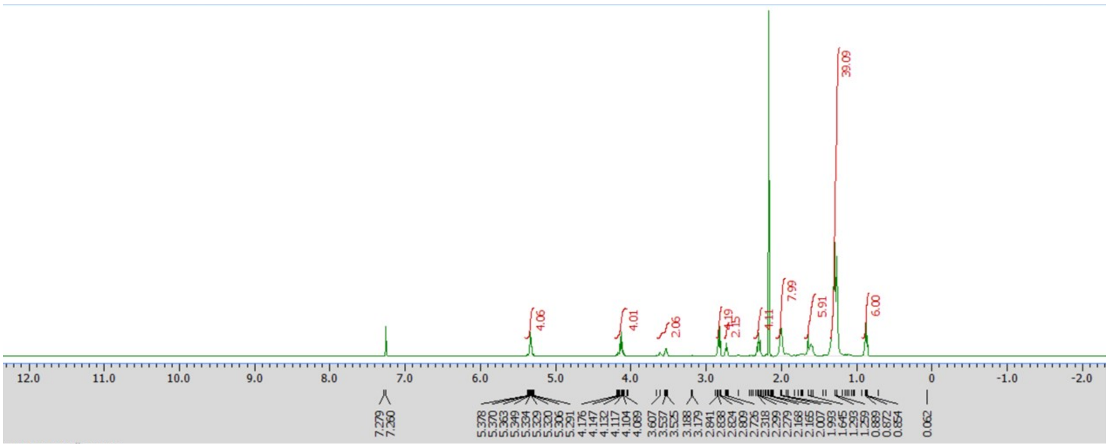

^{1}H-NMR (400 MHz $CDCl_3$)

δ: 0.85-0.93 (6H, m), 1.26-1.29 (30H, m), 1.59-1.65 (6H,m), 1.99-2.01 (8H,d), 2.13-2.18 (8H,m), 2.28-2.32 (4H, t, *J* = 2.0 Hz), 2.71-2.74 (4H,m), 2.81-2.88 (4H,m), 3.51-3.66 (2H, m), 4.10-4.19 (4H, m), 5.29-5.37 (4H, m) ppm.

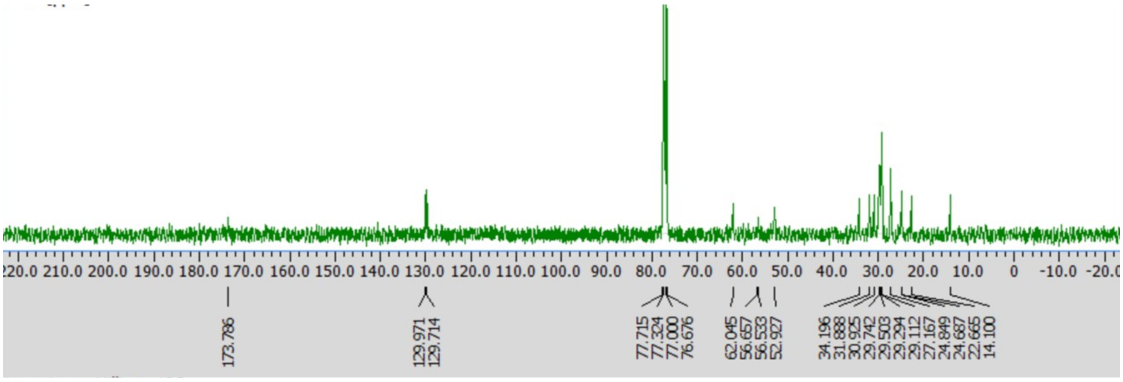

^{13}C-NMR (400 MHz $CDCl_3$)

δ: 14.100, 22.665, 24.687, 24.849, 27.167, 29.112, 29.294, 29.503, 29.742, 30.925, 31.888, 34.196, 52.927, 56.533, 56.657, 62.045, 129.714, 129.971, 173.786 ppm.

HRMS(ESI) calcd. for $C_{42}H_{79}NO_5$ $[M+H]^+$ 677.5995 found 677.5990.

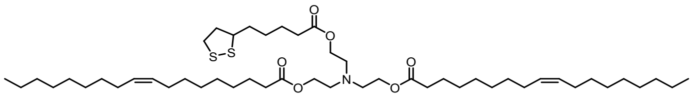

C18-1-A

C18-1-A’ (0.33 g, 0.48 mmol, 1.0 eq) was dissolved in DCM 10 mL a-Lipoic acid (0.12 g, 0.58 mmol, 1.2 eq) and DMAP (70 mg, 0.58 mmol, 1.2 eq) were added. Then stirred at 0℃. DCC (0.12 g, 0.58 mmol, 1.2 eq) was dissolved in DCM 4 mL and added to the solution by dropwise. After checking the completion of the reaction by TLC, residue was removed by filtering. Then evaporate and purified by column chromatography (DCM/MeOH=15/1). The target material (0.37 g, yield 90%) was obtained.

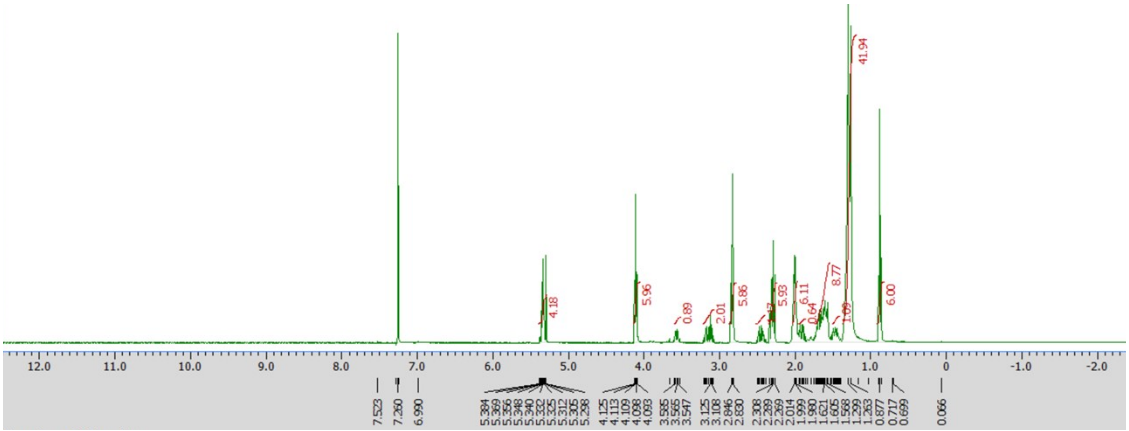

^{1}H-NMR 400 MHz $CDCl_3$

δ: 0.860-0.877 (6H, t, J = 6.8 Hz), 1.164-1.299 (42H, m), 1.394-1.68 (1H,m), 1.605-1.693 (9H,m), 1.704-1.882 (1H, m), 1.980-2.014 (6H, m), 2.269-2.336 (6H, m), 2.387-2.502 (1H, m), 2.814-2.846 (6H, t, J = 6.4 Hz), 3.080-3.212 (2H, m), 3.531-3.601 (1H, m), 4.093-4.129 (6H, m), 5.298-5.384 (4H, m) ppm.

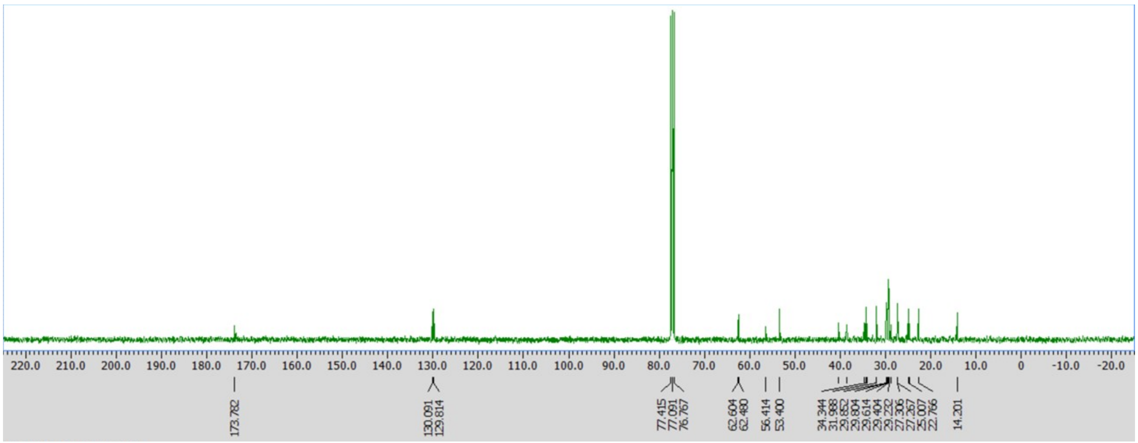

^{13}C-NMR (400 MHz $CDCl_3$)

δ: 14.201, 22.766, 25.007, 27.267, 27.306, 29.232, 29.404, 29.614, 29.804, 29.852, 31.988, 34.344, 53.400, 56.414, 62.480, 62.604, 129.814, 130.091, 173.782 ppm.

HRMS(ESI) calcd. for $C_{50}H_{91}NO_6S_2$ $[M+H]^+$ 866.6296 found 866.6322.

C18-0-A

Palmiltic acid (0.68 g, 2.68 mmol, 2.0 eq) was dissolved in DCM 15 mL. Triethanolamine (0.2 g, 1.34 mmol, 1.0 eq) and DMAP (0.33 g, 2.68 mmol, 2.0 eq) were added. Then stirred at 0 °C. DCC (0.55 g, 2.68 mmol, 2.0 eq) was dissolved in DCM 6 mL and added to the solution by dropwise. After checking the completion of the reaction by TLC, residue was removed by filtering, evaporated completely. The reaction mixture (0.10 g, 0.14 mmol, 1.0 eq) was dissolved in DCM 1 mL. a-Lipoic acid (31 mg, 0.15 mmol, 1.1 eq) and DMAP (18 mg, 0.15 mmol, 1.1 eq) were added. Then stirred at 0°C. DCC (31 mg, 0.15 mmol, 1.1 eq) was dissolved in DCM 1 mL and added to the solution by dropwise. After checking the completion of the reaction by TLC, residue was removed by filtering, then evaporated and purified by column chromatography (DCM/MeOH=15/1). The target material (91 mg, yield 75 %) was obtained.

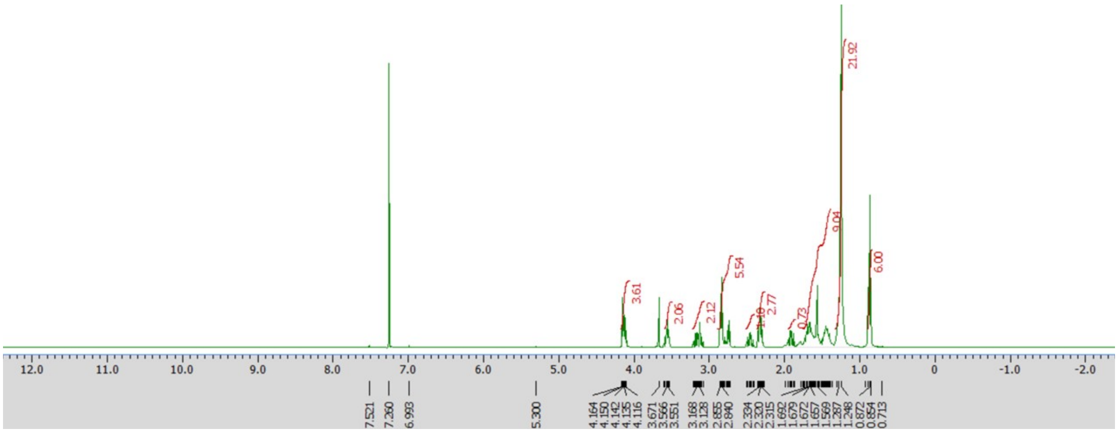

^{1}H-NMR (400MHz $CDCl_3$)

δ: 0.854-0.872 (6H, m), 1.248-1.287 (22H, m), 1.569-1.672 (9H, m), 1.867-1.987 (1H, m), 2.300-2.334 (3H, m), 2.425-2.489 (1H, m), 2.729-2.855 (6H, m), 3.100-3.186 (2H, m), 3.526-3.604 (2H, m), 4.101-4.167 (4H, m) ppm.

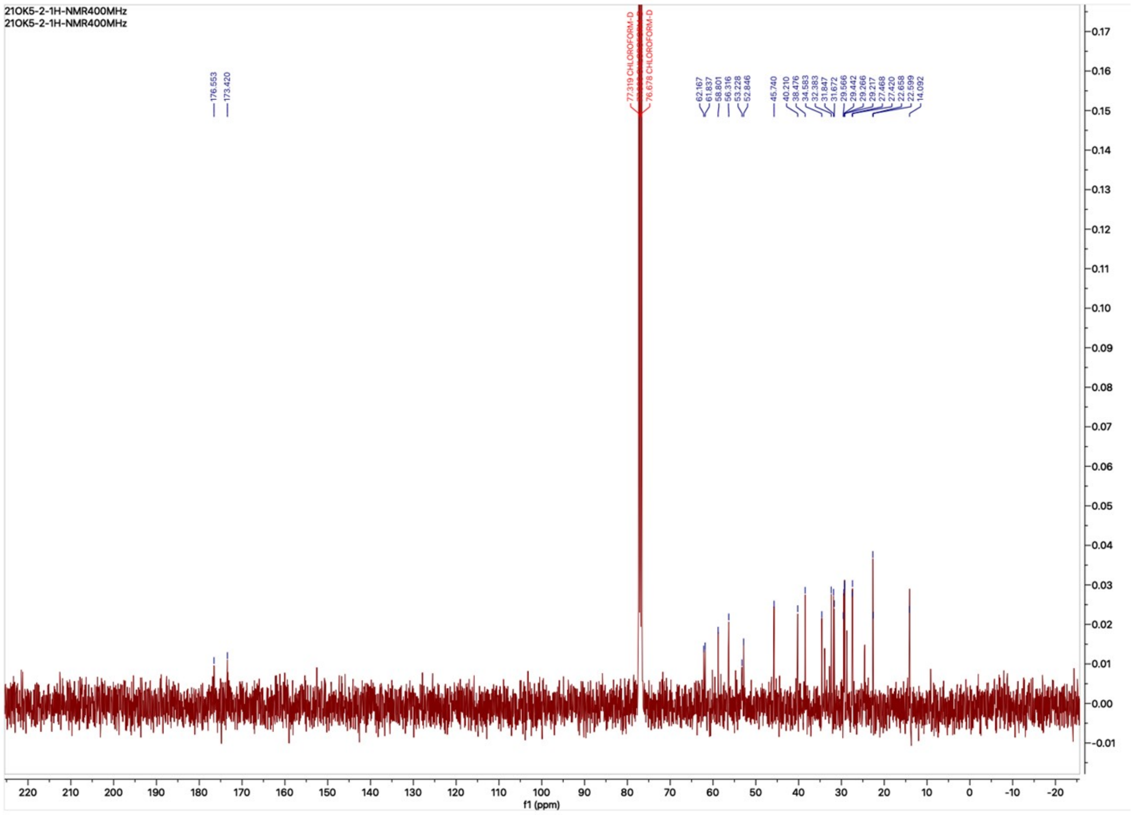

^{13}C-NMR (400MHz $CDCl_3$)

δ: 14.092, 22.658, 27.420, 22.468, 29.217, 29.266, 29.442, 31.672, 31.847, 32.383, 38.476, 40.210, 45.740, 52.846, 53.228, 56.316, 56.421, 58.801, 61.837, 62.167, 173.420, 176.553 ppm.

HRMS(ESI) calcd. for $C_{50}H_{95}NO_6S_2$ $[M+Na]^+$ 892.6498 found 892.6477.

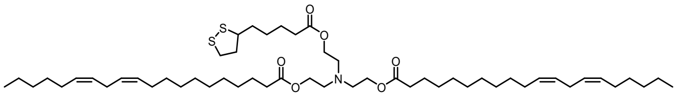

C20-2-A

11,14-Eicosadienoic acid (0.68 g, 2.68 mmol, 2.0 eq) was dissolved in DCM 15 mL. Triethanolamine (0.20 g, 1.34 mmol, 1.0 eq) and DMAP (0.33 g, 2.68 mmol, 2.0 eq) were added. Then stirred at 0°C. DCC (0.55 g, 2.68 mmol, 2.0 eq) was dissolved in DCM 6 mL and added to the solution by dropwise. After checking the completion of the reaction by TLC, residue was removed by filtering, evaporated completely. The reaction mixture (0.11 g, 0.14 mmol, 1.0 eq) was dissolved in DCM 1 mL. a-Lipoic acid (30 mg, 0.15 mmol, 1.1 eq) and DMAP (18 mg, 0.15 mmol, 1.1 eq) were added. Then stirred at 0°C. DCC (31 mg, 0.15 mmol, 1.1 eq) was dissolved in DCM 1 mL and added to the solution by dropwise. After checking the completion of the reaction by TLC, residue

was removed by filtering. Then evaporate and purified by column chromatography (DCM/MeOH=15/1). The target material (61 mg, yield 46 %) was obtained.

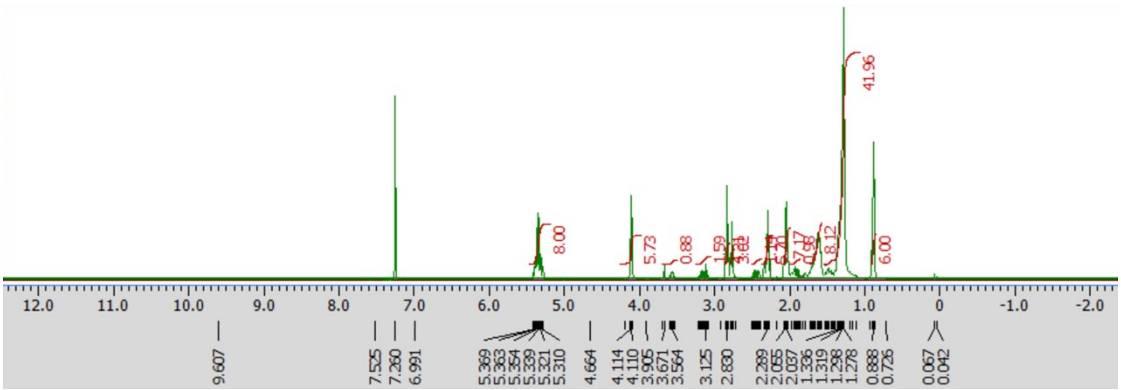

^{1}H-NMR (400MHz CDCl$_3$)

δ: 0.869-0.905 (6H, t, J = 7.2 Hz), 1.121-1.336 (42H, m), 1.602-1.693 (8H, m), 1.882-1.950 (1H, m), 2.019-2.072 (6H, m), 2.386-2.502 (1H, m), 2.754-2.770 (4H, t, J = 6.4 Hz), 2.830-2.846 (4H, t, J = 6.4 Hz), 3.108-3.183 (2H, m), 3.547-3.692 (1H, m), 4,110-4.114 (6H, d), 5.310-5.369 (8H, m) ppm.

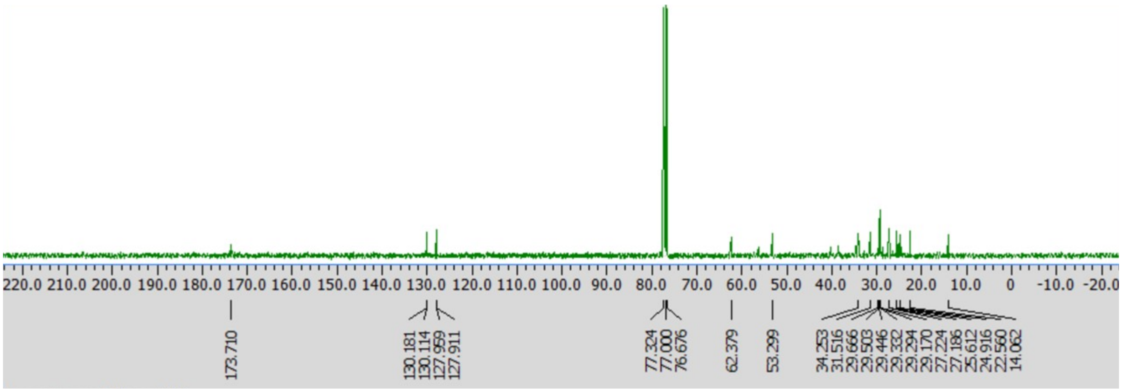

^{13}C-NMR (400MHz CDCl$_3$)

δ: 14.062, 22.560, 24.916, 25.612, 27.186, 27.224, 29.170, 29.294, 29.332, 29.446, 29.503, 29.666, 31.516, 34.253, 53.299, 62.379, 127.911, 127.959, 130.114, 130.181, 173.710 ppm.

HRMS(ESI) calcd. for $C_{54}H_{95}NO_6S_2$ $[M+Na]^+$ 940.6498 found 940.6501.

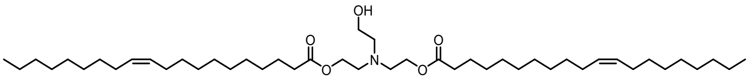

C20-1-A’

Cis-11-Eicosenoic acid (0.10 g, 0.32 mmol, 2.0 eq) was dissolved in DCM 10 mL. Triethanolamine (24 mg, 0.16 mmol, 1.0 eq) and DMAP (39 mg, 0.16 mmol, 2.0 eq) were added. Then stirred at 0℃. DCC (66 mg, 0.16 mmol, 2.0 eq) was dissolved in DCM 6 mL and added to the solution by dropwise. After checking the completion of the reaction by TLC, residue was removed by filtering. Then evaporate and purified by column chromatography (DCM/MeOH=15/1). The target material (0.10 g, yield 85%) was obtained.

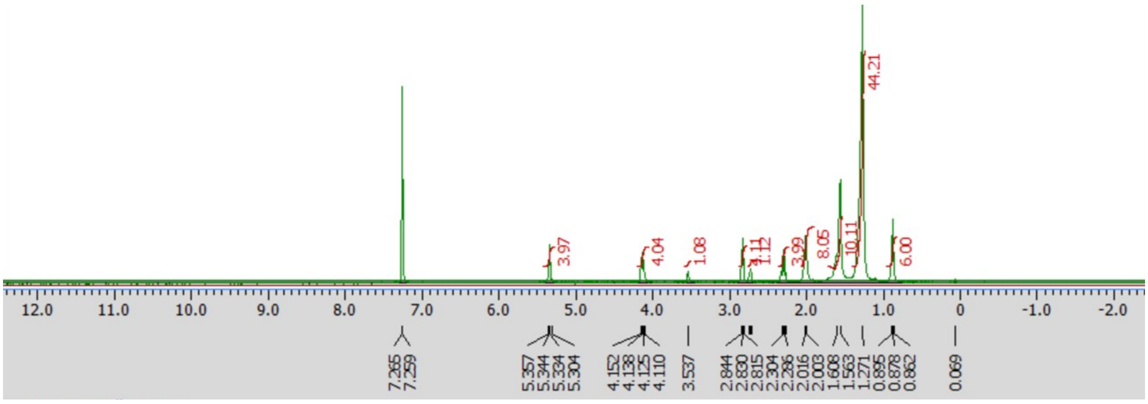

^{1}H-NMR (400MHz CDCl$_3$)

δ: 0.862-0.895 (6H, t, J = 6.4 Hz), 1.271 (44H, m), 1.563-1.608 (10H, m), 2.003-2.016 (8H, d), 2.268-2.322 (4H, m), 2.717-2.743 (1H, t, J = 5.2 Hz), 2.815-2.844 (4H, t, J = 6.0 Hz), 3.537 (s, 1H), 4.096-4.152 (4H, m), 5.304-5.357 (4H, m) ppm.

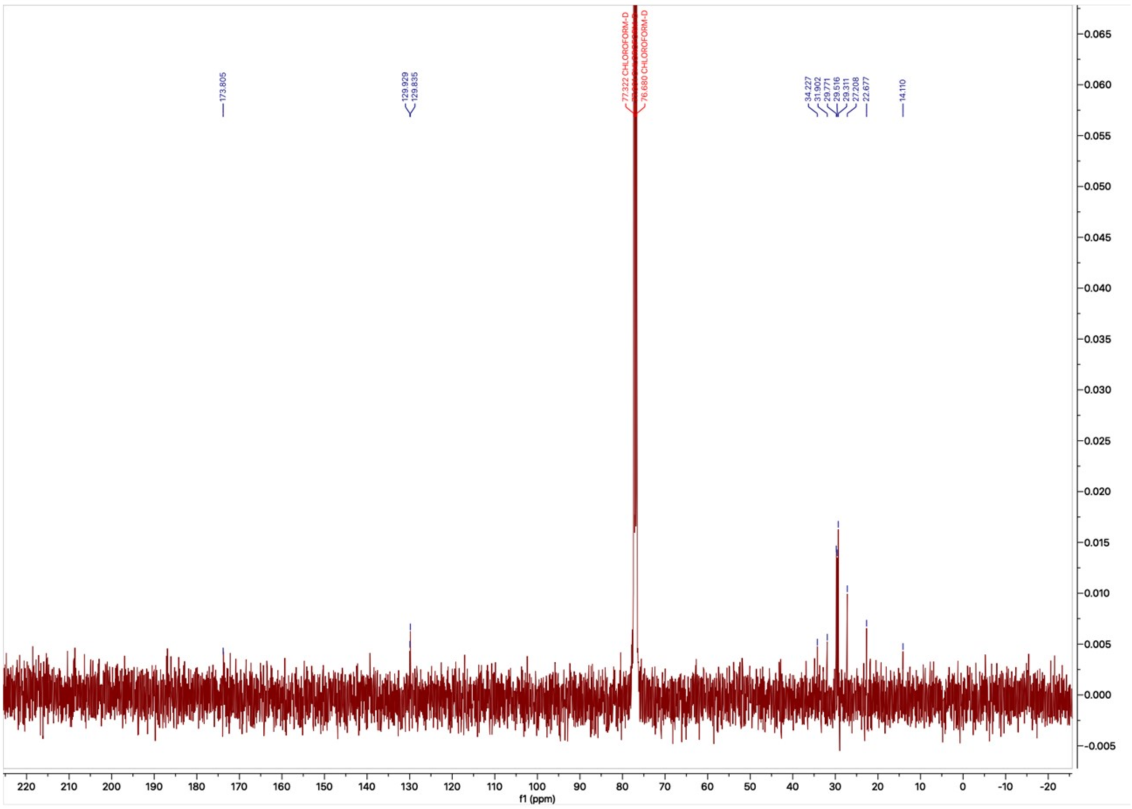

^{13}C-NMR (400MHz CDCl$_3$)

δ: 14.110, 22.677, 27.208, 29.311, 29.516, 29.771, 31.902, 34.227, 129.835, 129.929, 173.805 ppm.

HRMS(ESI) calcd. for $C_{46}H_{87}NO_5$ $[M+H]^+$ 734.6592 found 734.6590.

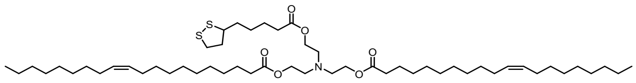

C20-1-A

C20-1-A’ (0.10 g, 0.14 mmol, 1.0 eq) was dissolved in DCM 10 mL. a-Lipoic acid (35 mg, 0.17 mmol, 1.2 eq) and DMAP (22 mg, 0.17 mmol, 1.2 eq) were added. Then stirred at 0℃. DCC (35 mg, 0.17 mmol, 1.2 eq) was dissolved in DCM 4 mL and added to the solution by dropwise. After checking the completion of the reaction by TLC, residue was removed by filtering. Then evaporate and purified by column chromatography (DCM/MeOH=15/1). The target material (61mg, yield 47 %) was obtained.

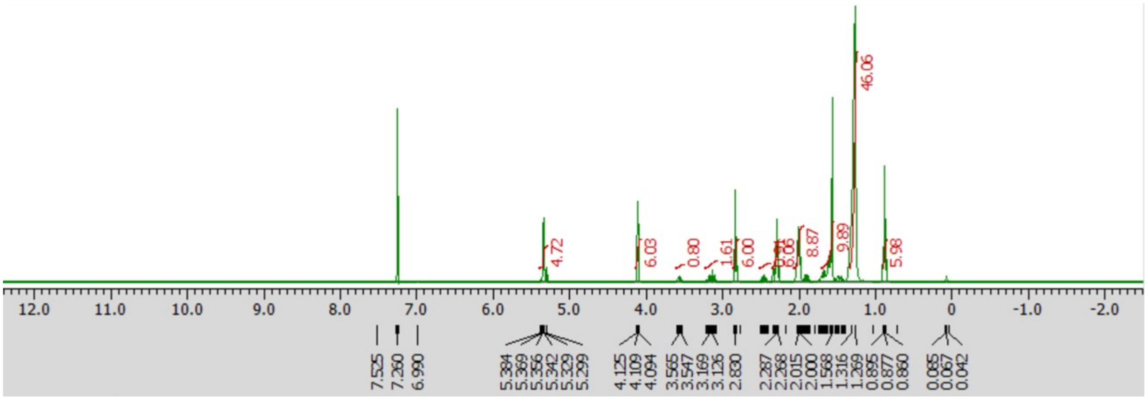

^{1}H-NMR (400MHz CDCl$_3$)

δ: 0.860-0.895 (6H, t, J = 7.2 Hz), 1.269-1.316 (46H, m), 1.583-1.693 (10H, m), 1.983-2.031 (9H, m), 2.268-2.316 (6H, m), 2.405-2.503 (1H, m), 2.814-2.846 (6H, t, J = 6.4 Hz), 3.080-3.212 (2H, m), 3.531-3.601 (1H, m), 4.094-4.125 (6H, t, J = 6.4 Hz), 5.299-5.384 (5H, m) ppm.

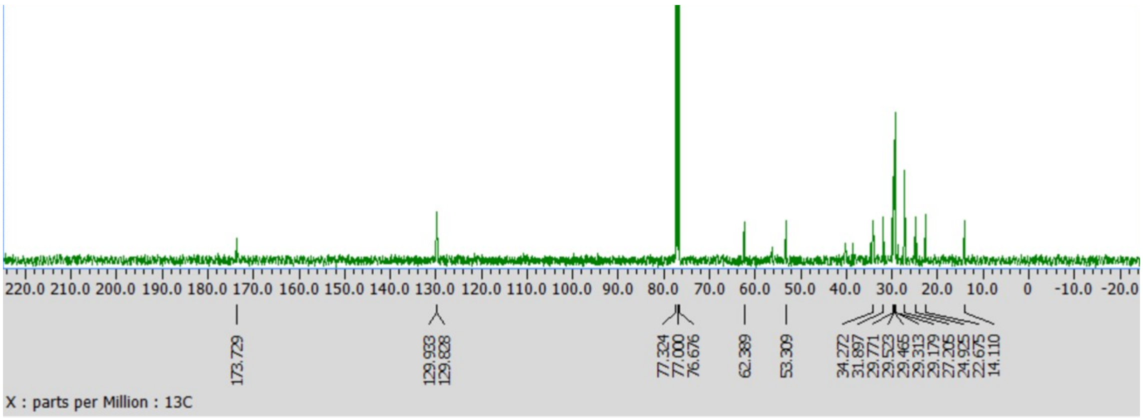

^{13}C-NMR (400MHz CDCl$_3$)

δ: 14.110, 22.675, 24.925, 27.205, 29.179, 29.313, 29.465, 29.523, 29.771, 31.897, 34.272, 53.309, 62.389, 129.828, 129.933, 173.729 ppm.

HRMS(ESI) calcd. for $C_{54}H_{99}NO_6S_2$ $[M+Na]^+$ 944.6811 found 944.6799.

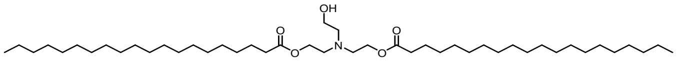

C20-0-A'

Arachidic acid (0.30 g, 0.96 mmol, 2.0 eq) was dissolved in DCM 8 mL. Triethanolamine (69 mg, 0.48 mmol, 1.0 eq) and DMAP (119 mg, 0.96 mmol, 2.0 eq) were added. Then stirred at 0℃. DCC (0.20 g, 0.96 mmol, 2.0 eq) was dissolved in DCM 6 mL and added to the solution by dropwise. After checking the completion of the reaction by TLC, residue was removed by filtering. Then evaporated and purified by column chromatography (DCM/MeOH=15/1). The target material (0.48 g, yield 64%) was obtained.

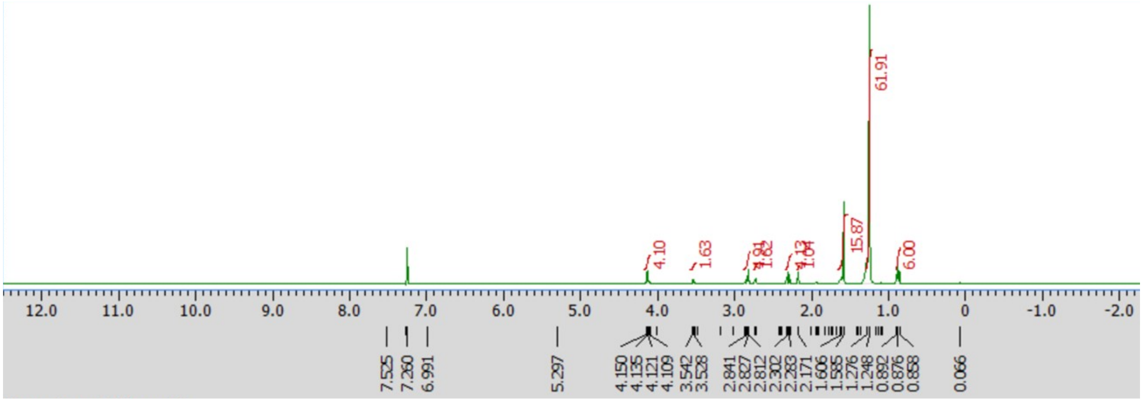

^{1}H-NMR (400MHz $CDCl_3$)

δ: 0.858-0.892 (6H, t, J = 7.2 Hz), 1.161-1.363 (62H, m), 1.585-1.676 (16H, m), 2.171 (1H, s), 2.283-2.308 (4H, m), 2.714-2.740 (2H, t, J = 5.2 Hz), 2.812-2.841 (5H, m), 3.481-3.555 (2H, m), 4.109-4.150 (4H, m) ppm.

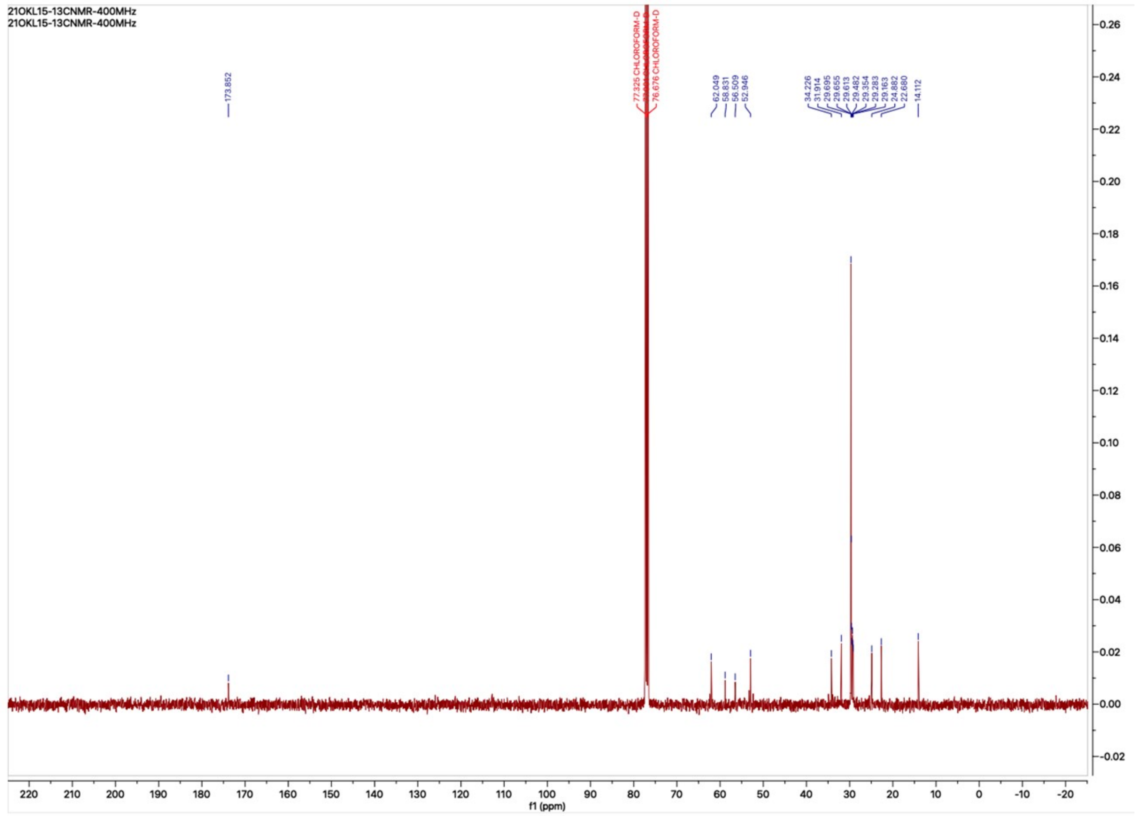

^{13}C-NMR (400MHz $CDCl_3$)

δ: 14.112, 22.680, 24.882, 29.163, 29.283, 29.354, 29.482, 29.695, 31.914, 34.226, 52.946, 56.509, 58.831, 62.049, 173.852 ppm.

HRMS(ESI) calcd. for $C_{46}H_{91}NO_5$ $[M+H]^+$ 738.6905 found 738.6905.

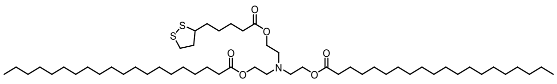

C20-0-A

C20-0-A' (0.10 g, 0.14 mmol, 1.0 eq) was dissolved in DCM 9 mL. a-Lipoic acid (35 mg, 0.17 mmol, 1.2 eq) and DMAP (22 mg, 0.17 mmol, 1.2 eq) were added. Then stirred at 0℃. DCC (35 mg, 0.17 mmol, 1.2 eq) was dissolved in DCM 4 mL and added to the solution by dropwise. After checking the completion of the reaction by TLC, residue was removed by filtering. Then evaporated and purified by column chromatography (DCM/MeOH=15/1). The target material (97 mg, yield 75%) was obtained.

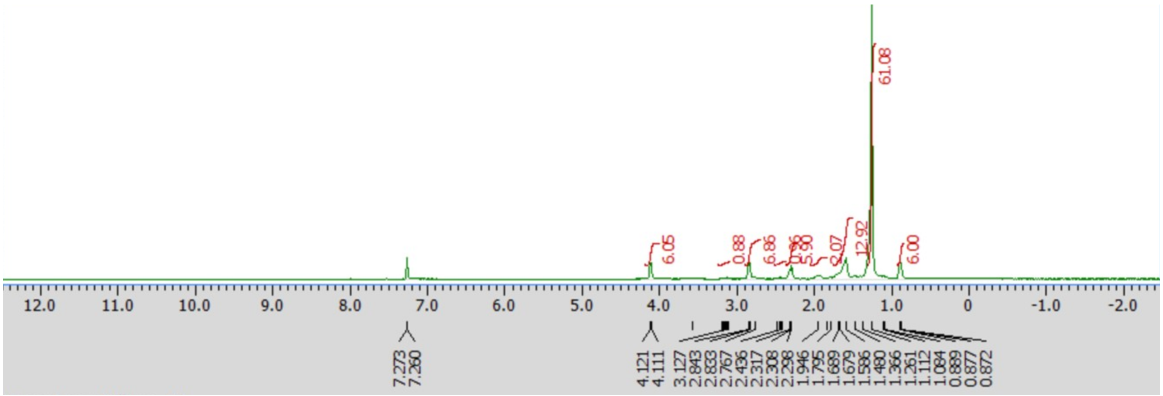

^{1}H-NMR (400MHz $CDCl_3$)

δ: 0.872-0.889 (6H, t, J = 4.8 Hz), 1.261-1.366 (61H, m), 1.586-1.689 (13H, m), 1.946 (2H, s), 2.298-2.317 (6H, m), 2.409-2.482 (1H, m), 2.767-2.843 (7H, m), 3.109-3.195 (1H, m), 4.111-4.121 (6H, d) ppm.

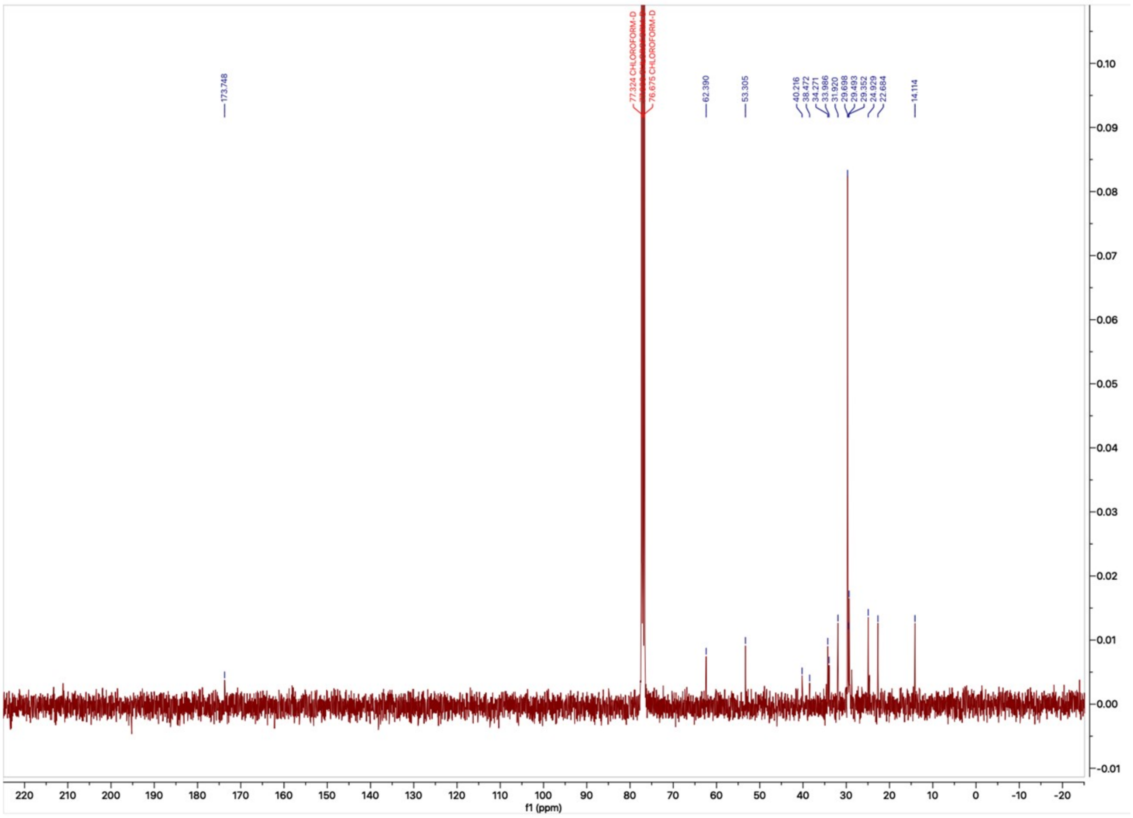

^{13}C-NMR (400MHz $CDCl_3$)

δ: 14.114, 22.684, 24.929, 24.725, 29.352, 29.493, 29.698, 31.920, 33.738, 33.986, 34.271, 38.472, 40.216, 53.305, 62.390, 173.748 ppm.

HRMS(ESI) calcd. for $C_{54}H_{103}NO_6S_2$ $[M+Na]^+$ 948.7124 found 948.7113.

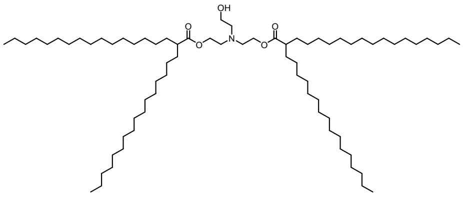

C18-0-B'

2-Hexadecyloctadecanoic Acid (0.20 g, 0.39 mmol, 2.0 eq) was dissolved in DCM 10 mL. Triethanolamine (28 mg, 0.20 mmol, 1.0 eq) and DMAP (48 mg, 0.39 mmol, 2.0 eq) were added. Then stirred at 0℃. DCC (80 mg, 0.39 mmol, 2.0 eq) was dissolved in DCM 5 mL and added to the solution by dropwise, stirred overnight. After checking the completion of the reaction by TLC, residue was removed by filtering. Then evaporated and purified by column chromatography (DCM/MeOH=15/1). The target material (0.28 g, yield 64%) was obtained.

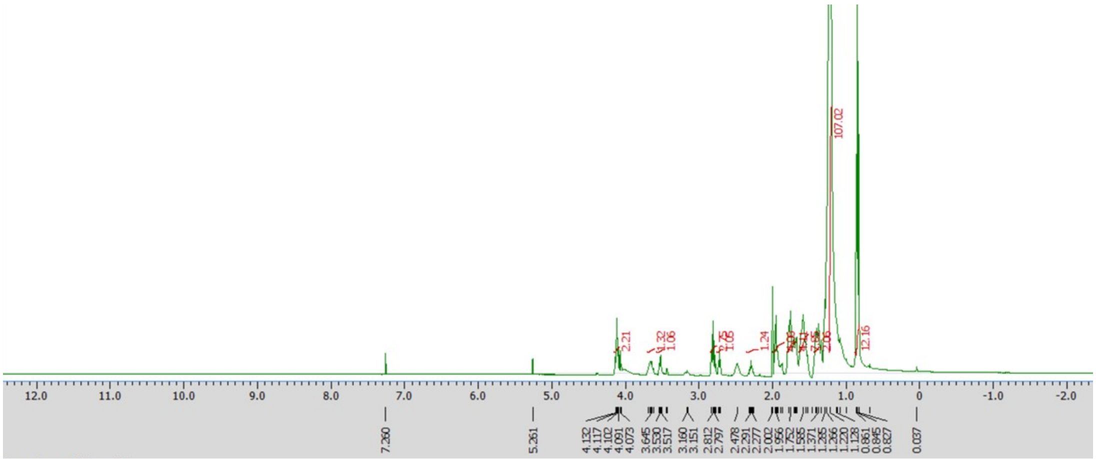

^{1}H-NMR (400 MHz $CDCl_3$)

δ: 0.827-0.861(12H, t, *J* = 7.2 Hz), 1.118-1.285 (107H, m), 1.338-1.411 (2H, m), 1.547-1.585 (8H, m), 1.676-1.778 (4H, m), 1.870-1.956 (4H, m), 2.269-2.313 (1H, m), 2.704-2.731 (1H, t, *J* = 5.6 Hz), 2.782-2.827 (3H, m) ppm.

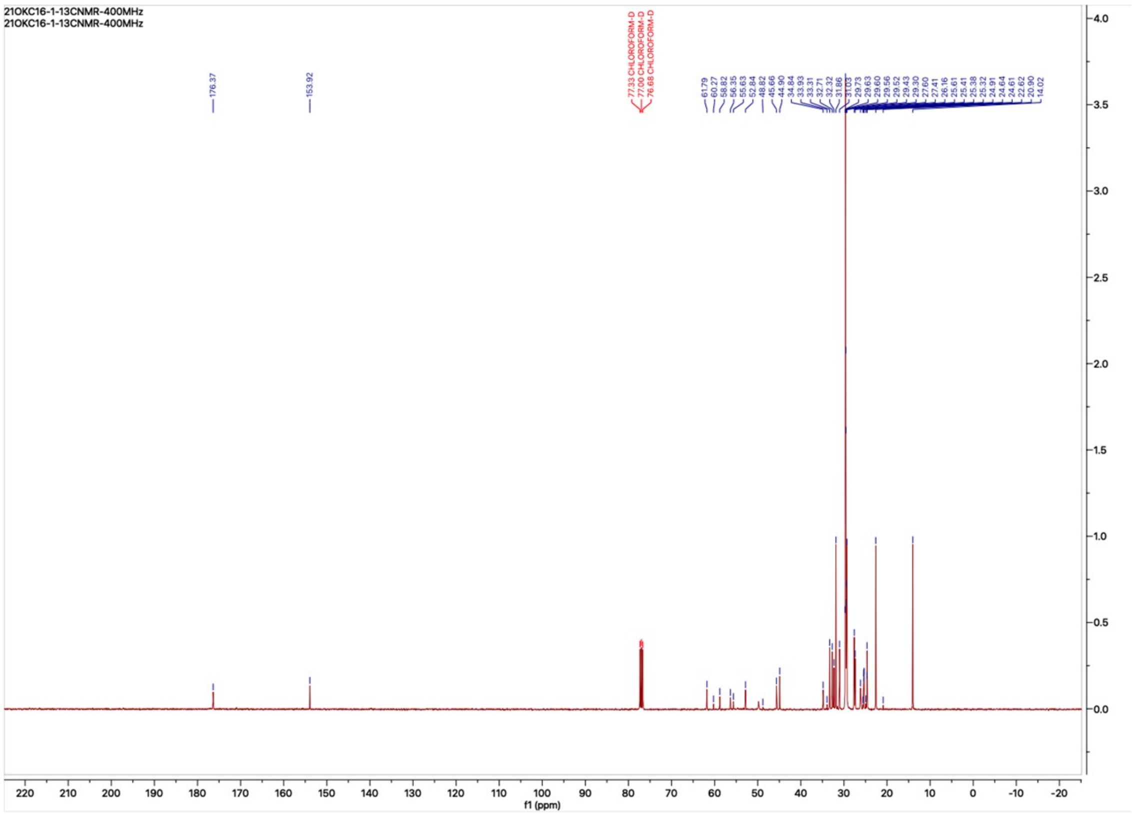

^{13}C-NMR (400 MHz $CDCl_3$)

δ: 14.024, 22.617, 24.649, 27.415, 27.606, 29.303, 29.473, 29.637, 29.732, 31.029, 31.859, 32.327, 32.718, 33.318, 44.897, 45.669, 52.842, 61.788, 153.920, 176.361 ppm.

HRMS(ESI) calcd. for $C_{74}H_{147}NO_2$ $[M+Na]^+$ 1153.1176 found 1153.1172.

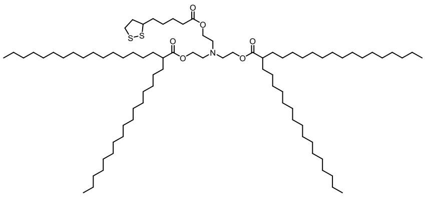

C18-0-B

C18-0-B' (0.28 g, 0.25 mmol, 1.0 eq) was dissolved in DCM 7 mL. a-Lipoic acid (62 mg, 0.30 mmol, 1.2 eq) and DMAP (43 mg, 0.30 mmol, 1.2 eq) were added. Then stirred at 0°C. DCC (62 mg, 0.30 mmol, 1.2 eq) was dissolved in DCM 2 mL and added to the solution by dropwise. After checking the completion of the reaction by TLC, residue was removed by filtering. Then evaporate and purified by column chromatography (DCM/MeOH=15/1). The target material (96 mg, yield 29%) was obtained.

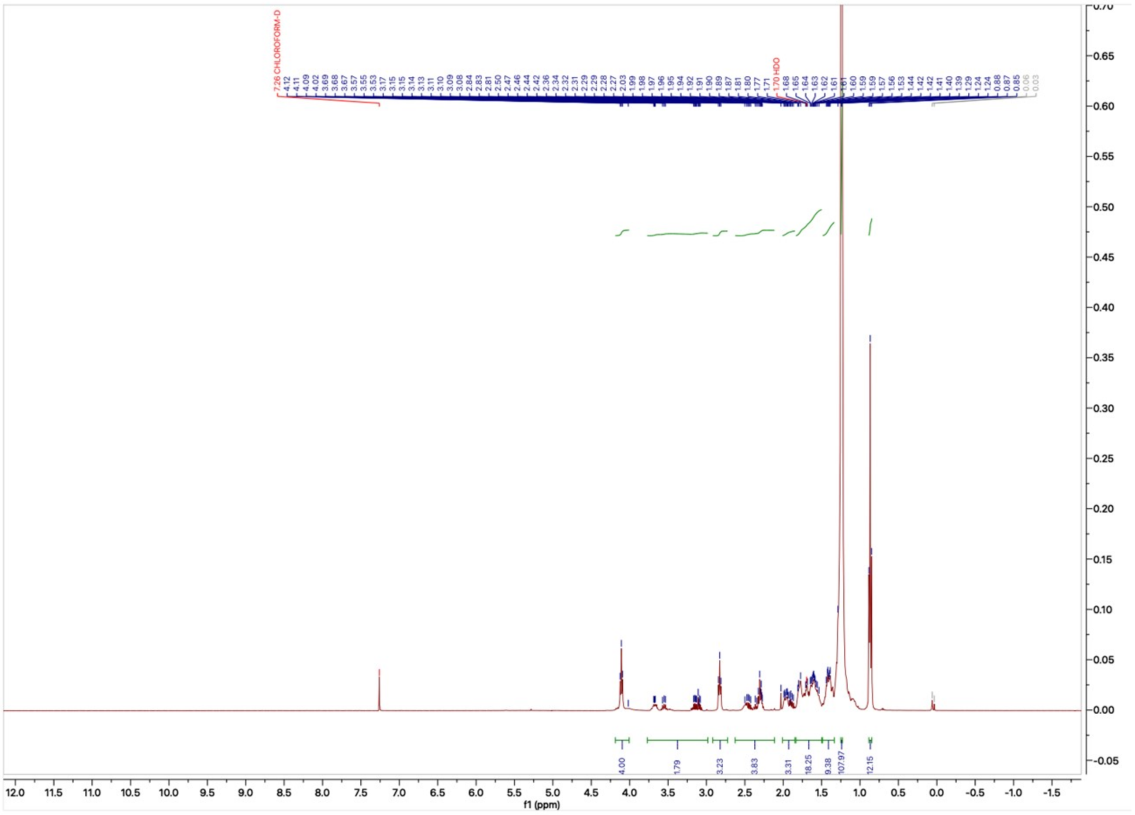

^{1}H-NMR (400 MHz $CDCl_3$)

δ: 0.849-0.884(12H, t, *J* = 7.2 Hz), 1.146-1.287 (108H, m), 1.389-1.420 (4H, m), 1.571-1.775 (16H, m), 1.901-2.030 (3H, m), 2.273-2.472 (5H, m), 2.811-2.842 (4H, t, *J* = 6.0 Hz), 3.080-3.167 (1H, m), 3.534-3.686 (2H, m), 4.093-4.124 (4H, t, *J* = 6.4 Hz) ppm.

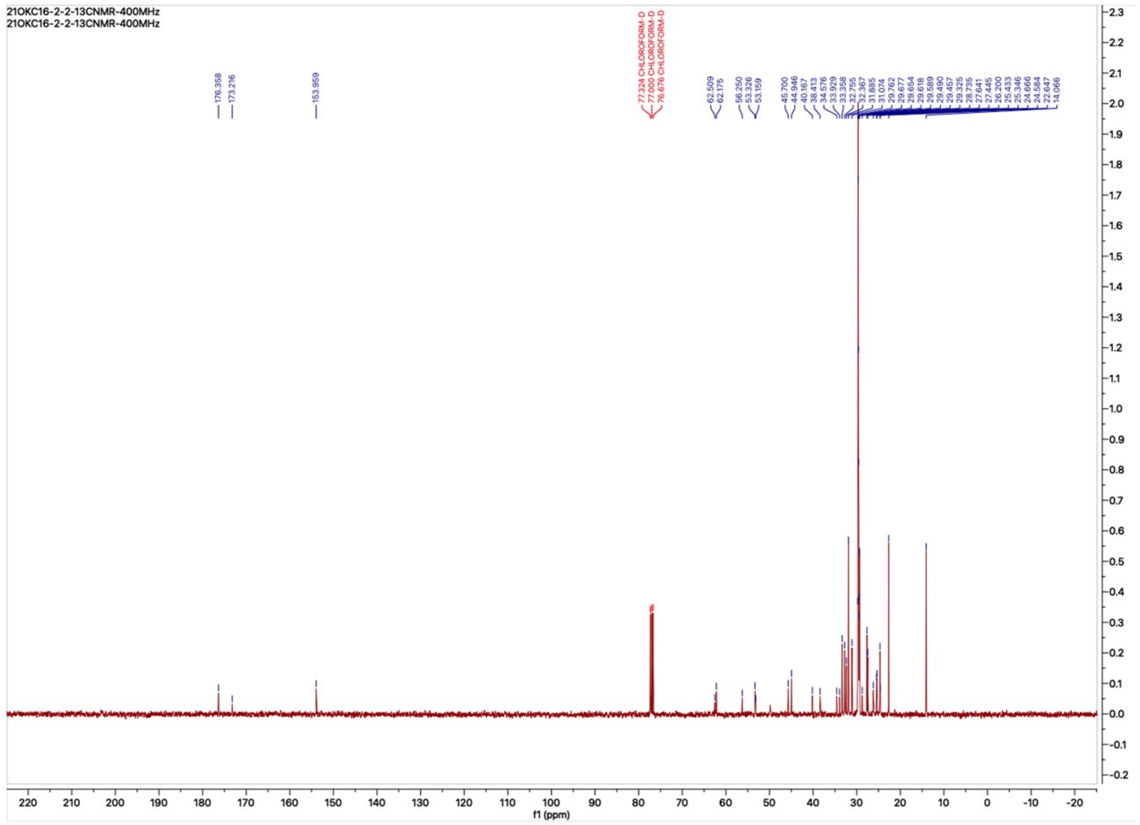

^{13}**C-NMR (400 MHz $CDCl_3$)**

δ: 14.072, 22.656, 24.678, 27.453, 27.653, 29.332, 29.465, 29.494, 29.666, 29.771, 31.077, 31.897, 32.765, 33.366, 38.421, 40.176, 44.954, 45.708, 49.837, 53.166, 53.328, 56.526, 62.179, 62.513, 153.958, 173.214, 176.352 ppm.

HRMS(ESI) calcd. for $C_{82}H_{159}NO_6S_2$ $[M+H]^+$ 1319.1689 found 1319.1734.

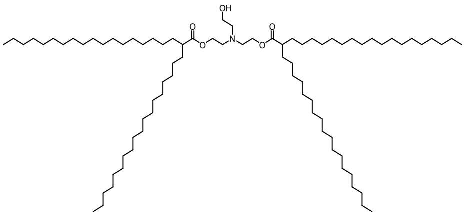

C20-0-B'

2-Octadecyleicosanoic Acid (0.30 g, 0.53 mmol, 2.0 eq) was dissolved in DCM 12 mL. Triethanolamine (37 mg, 0.26 mmol, 1.0 eq) and DMAP (65 mg, 0.53 mmol, 2.0 eq) were added. Then stirred at 0°C. DCC (0.11 g, 0.53 mmol, 2.0 eq) was dissolved in DCM 5 mL and added to the solution by dropwise, stirred overnight. After checking the completion of the reaction by TLC,

residue was removed by filtering. Then evaporated and purified by column chromatography (DCM/MeOH=15/1). The target material (0.38 g, yield 58%) was obtained.

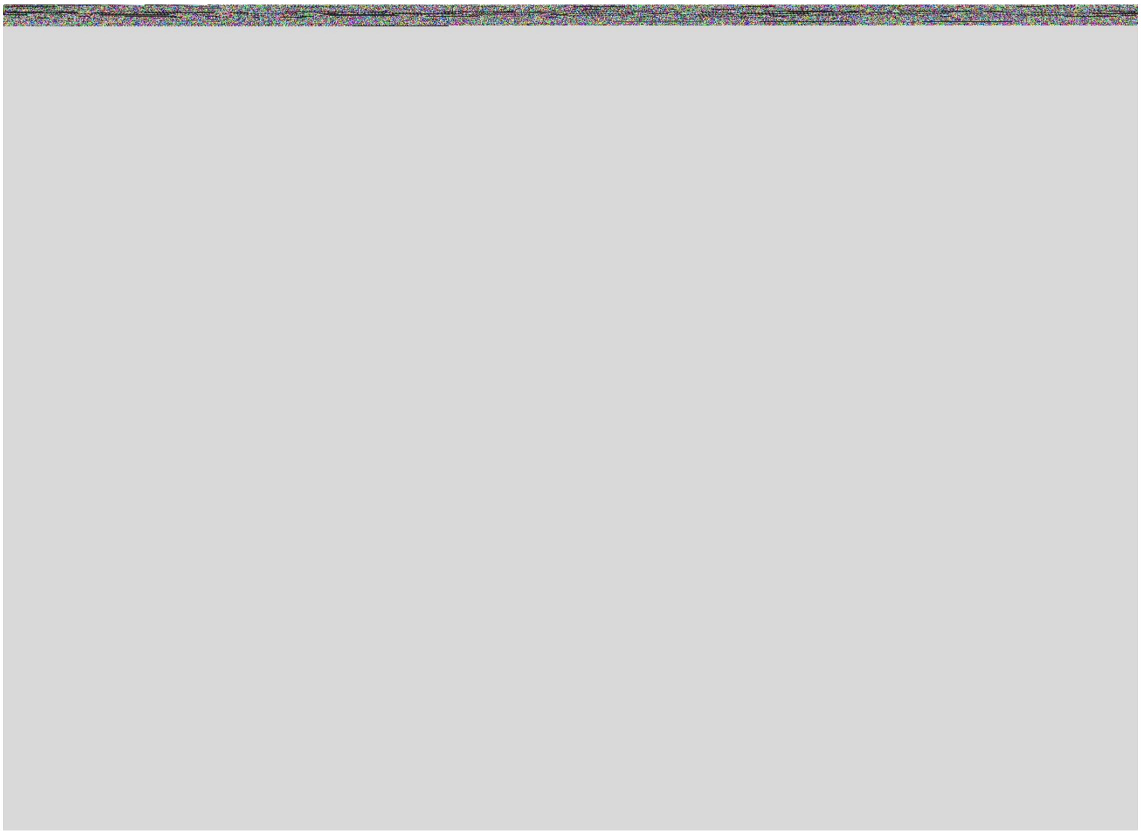

^{1}H-NMR (400 MHz $CDCl_3$)

δ: 0.856-0.890 (12H, t, *J* = 6.8 Hz), 1.110-1.291 (105H, m), 1.313-1.425 (7H, m), 1.567-1.622 (7H, m), 1.728-1.781 (8H, m), 1.900-2.038 (6H, m), 2.315-2.390 (3H, m), 2.486-2.501 (1H, d), 2.733-2.759 (2H, t, *J* = 5.2 Hz), 2.825-2.854 (3H, t, *J* = 6.0 Hz), 3.165-3.215 (1H, t). 3.482 (1H, s), 3.543 (1H, d), 3.659-3.691 (1H, d), 4.088-4.159 (4H, m) ppm.

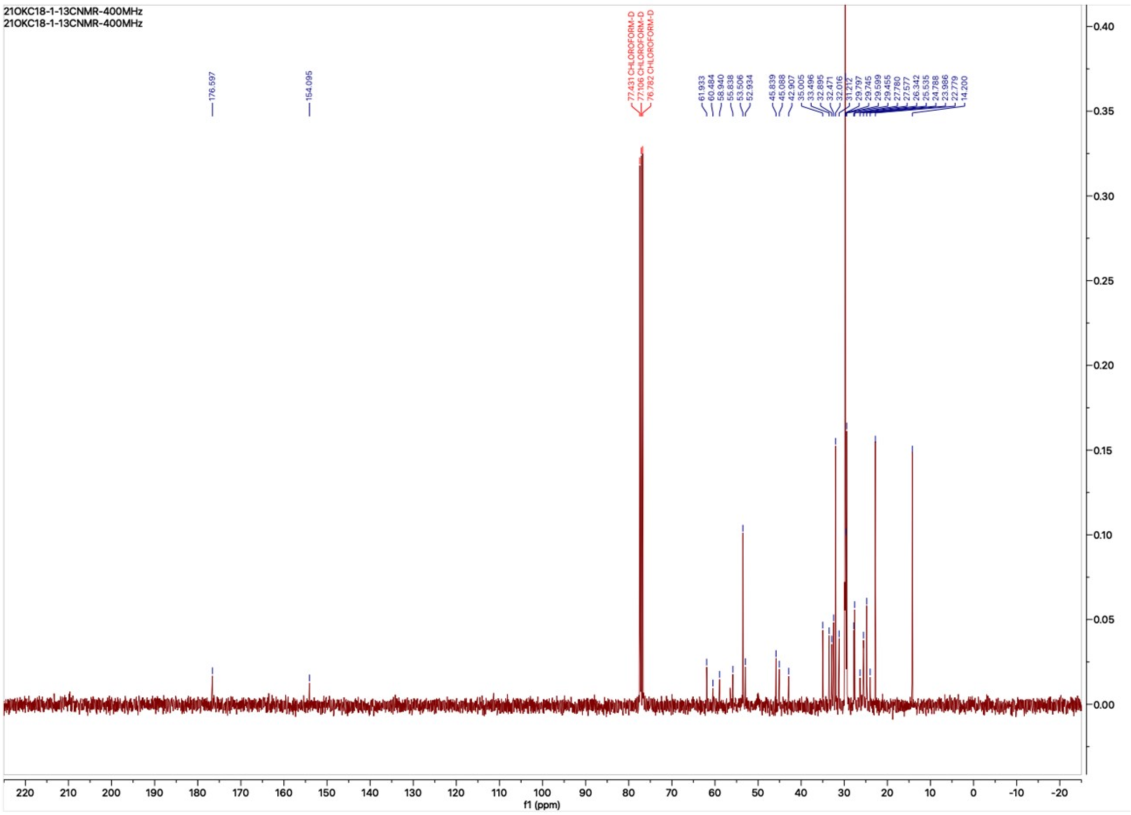

^{13}C-NMR (400 MHz CDCl$_3$)

δ: 14.100, 22.675, 24.687, 27.472, 29.351, 29.494, 29.694, 29.790, 31.907, 42.799, 44.983, 45.736, 52.823, 53.395, 55.731, 58.831, 61.806, 153.977, 176.476 ppm.

HRMS(ESI) calcd. for $C_{82}H_{163}NO_5$ $[M+H]^+$ 1243.2539 found 1243.2469.

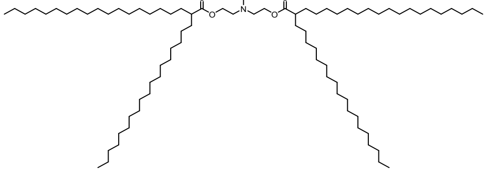

C20-0-B

C20-0-B' (0.10 g, 0.08 mmol, 1.0 eq) was dissolved in DCM 3 mL. a-Lipoic acid (31 mg, 0.16 mmol, 2.0 eq) and DMAP (23 mg, 0.16 mmol, 2.0 eq) were added. Then stirred at 0°C. DCC (31 mg, 0.16 mmol, 2.0 eq) was dissolved in DCM 1 mL and added to the solution by dropwise. After checking the completion of the reaction by TLC, residue was removed by filtering. Then evaporated and purified by column chromatography (DCM/MeOH=10/1). The target material (68 mg, yield 59%) was obtained.

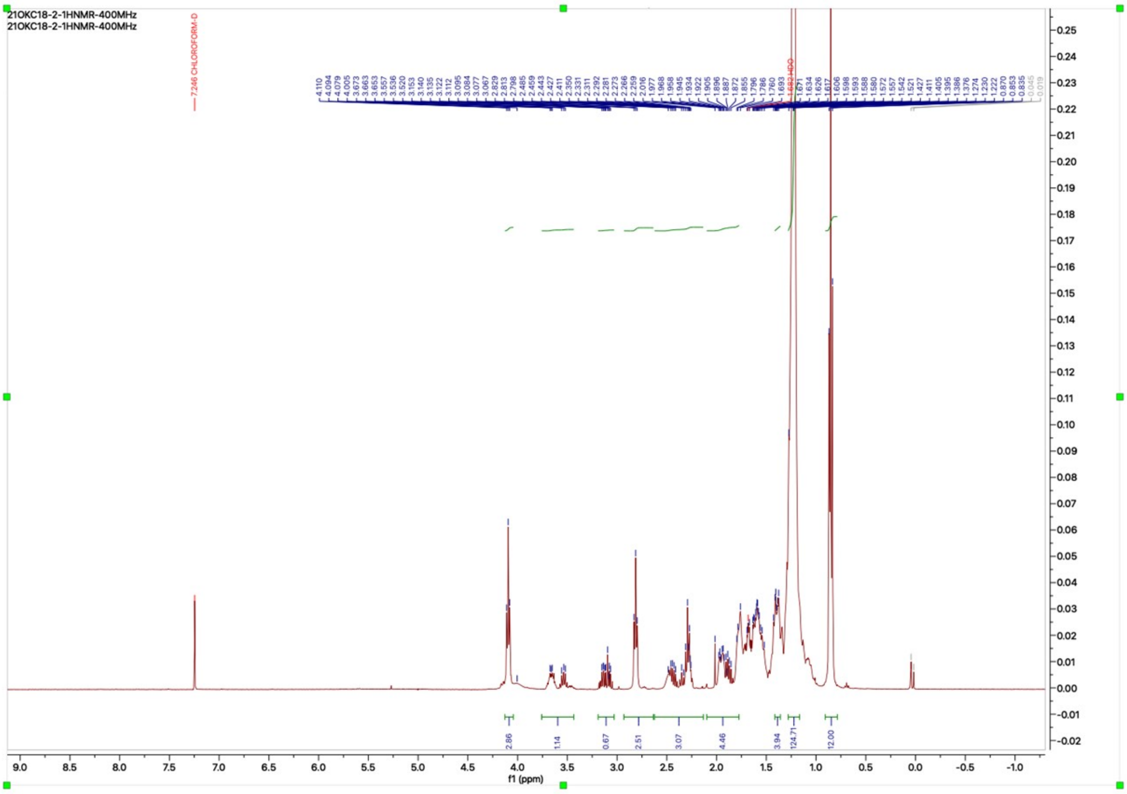

^{1}H-NMR (400 MHz $CDCl_3$)

δ: 0.849-0.884 (12H, t, J = 7.2 Hz), 1.146-1.287 (124H, m), 1.389-1.420 (4H, m), 1.571-1.775 (16H, m), 1.901-2.030 (3H, m), 2.273-2.472 (5H, m), 2.811-2.842 (4H, t, J = 6.0 Hz), 3.080-3.167 (1H, m), 3.534-3.686 (2H, m), 4.093-4.124 (4H, t, J = 6.4 Hz) ppm.

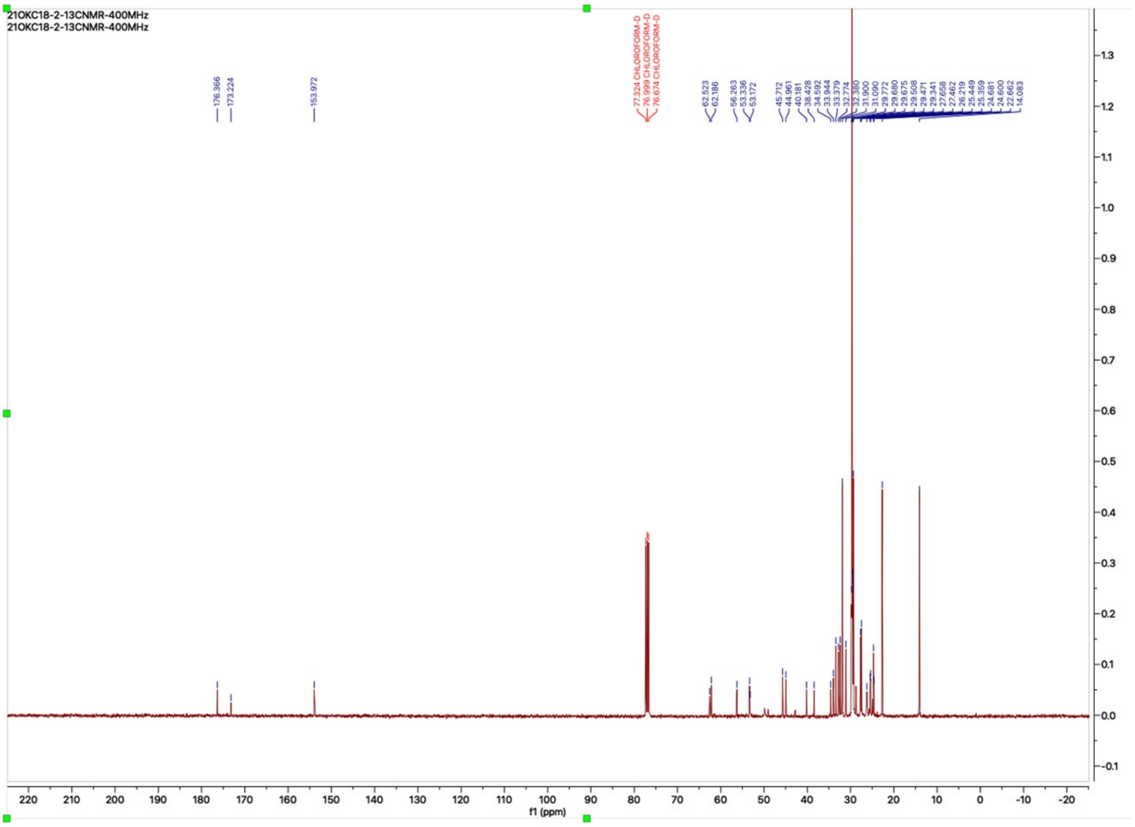

^{13}C-NMR (400 MHz CDCl$_3$)

δ: 14.083, 22.662, 24.600, 24.681, 25.359, 25.449, 27.462, 27.658, 29.341, 29.471, 29.508, 29.675, 29.772, 31.090, 31.900, 32.380, 32.774, 33.379, 33.944, 38.428, 40.181, 44.961, 45.712, 53.172, 53.336, 56.263, 62.186, 62.523, 153.972, 173.224, 176.366 ppm.

HRMS(ESI) calcd. for $C_{90}H_{175}NO_6S_2$ $[M+Na]^+$ 1454.2758 found 1454.2786.

Supplementary materials. Coding Sequences (CDS) for mRNAs used in this study.

NLuc

ATGGTCTTCACACTCGAAGATTTCGTTGGGGACTGGCGACAGACAGCCGGCTACAACCT
GGACCAAGTCCTTGAACAGGGAGGTGTGTCCAGTTTGTTTCAGAATCTCGGGGTGTCCG
TAACTCCGATCCAAAGGATTGTCCTGAGCGGTGAAAATGGGCTGAAGATCGACATCCAT
GTCATCATCCCGTATGAAGGTCTGAGCGGCGACCAAATGGGCCAGATCGAAAAAATTTT
TAAGGTGGTGTACCCTGTGGATGATCATCACTTTAAGGTGATCCTGCACTATGGCACAC
TGGTAATCGACGGGGTTACGCCGAACATGATCGACTATTTCGGACGGCCGTATGAAGGC
ATCGCCGTGTTCGACGGCAAAAAGATCACTGTAACAGGGACCCTGTGGAACGGCAACA
AAATTATCGACGAGCGCCTGATCAACCCCGACGGCTCCCTGCTGTTCCGAGTAACCATC
AACGGAGTGACCGGCTGGCGGCTGTGCGAACGCATTCTGGCGTAA

OVA

ATGGGCTCCATCGGTGCAGCAAGCATGGAATTTTGTTTTGATGTATTCAAGGAGCTCAA
AGTCCACCATGCCAATGAGAACATCTTCTACTGCCCCATTGCCATCATGTCAGCTCTAG
CCATGGTATACCTGGGTGCAAAAGACAGCACCAGGACACAAATAAATAAGGTTGTTCG
CTTTGATAAACTTCCAGGATTCGGAGACAGTATTGAAGCTCAGTGTGGCACATCTGTAA
ACGTTCACTCTTCACTTAGAGACATCCTCAACCAAATCACCAAACCAAATGATGTTTAT
TCGTTCAGCCTTGCCAGTAGACTTTATGCTGAAGAGAGATACCCAATCCTGCCAGAATA
CTTGCAGTGTGTGAAGGAACTGTATAGAGGAGGCTTGGAACCTATCAACTTTCAAACAG
CTGCAGATCAAGCCAGAGAGCTCATCAATTCCTGGGTAGAAAGTCAGACAAATGGAAT
TATCAGAAATGTCCTTCAGCCAAGCTCCGTGGATTCTCAAACTGCAATGGTTCTGGTTA
ATGCCATTGTCTTCAAAGGACTGTGGGAGAAAGCATTTAAGGATGAAGACACACAAGC
AATGCCTTTCAGAGTGACTGAGCAAGAAAGCAAACCTGTGCAGATGATGTACCAGATT
GGTTTATTTAGAGTGGCATCAATGGCTTCTGAGAAAATGAAGATCCTGGAGCTTCCATT
TGCCAGTGGGACAATGAGCATGTTGGTGCTGTTGCCTGATGAAGTCTCAGGCCTTGAGC
AGCTTGAGAGTATAATCAACTTTGAAAAACTGACTGAATGGACCAGTTCTAATGTTATG
GAAGAGAGGAAGATCAAAGTGTACTTACCTCGCATGAAGATGGAGGAAAAATACAACC
TCACATCTGTCTTAATGGCTATGGGCATTACTGACGTGTTTAGCTCTTCAGCCAATCTGT
CTGGCATCTCCTCAGCAGAGAGCCTGAAGATATCTCAAGCTGTCCATGCAGCACATGCA
GAAATCAATGAAGCAGGCAGAGAGGTGGTAGGGTCAGCAGAGGCTGGAGTGGATGCT
GCAAGCGTCTCTGAAGAATTTAGGGCTGACCATCCATTCCTCTTCTGTATCAAGCACAT
CGCAACCAACGCCGTTCTCTTCTTTGGCAGATGTGTTTCCCCTTAA

Supplementary Table 1. Structure, molecular weight, and yield of the alkyl chain of the synthesized cyclic disulfide-containing lipids (CDLs)

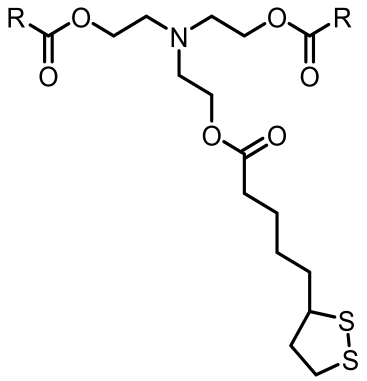

#Sample	Sample name	Alkyl residue□R=	MW	Yield （%）□
1	C16-0-A	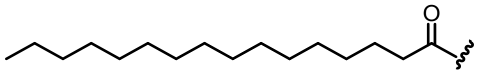	813.60	58
3	C16-1-A	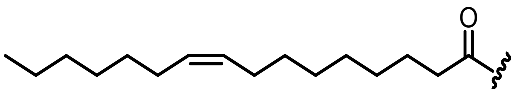	809.57	34
5	C18-0-A	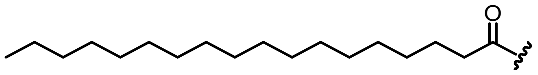	869.66	33
6	C18-0-B	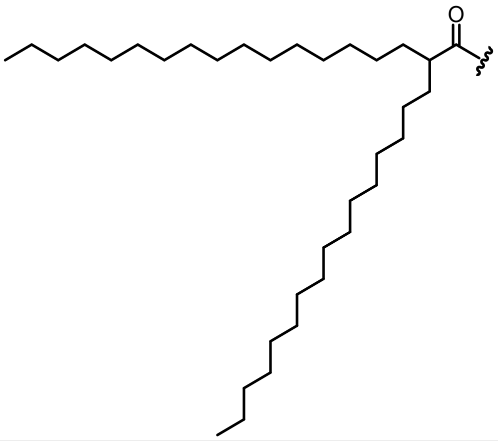	1318.16	19

7	C18-1-A	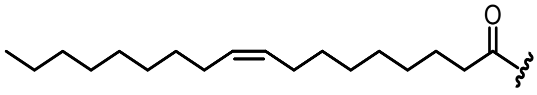	865.63	86
8	C18-2-A	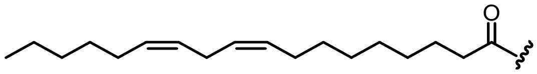	861.60	60
9	C20-0-A	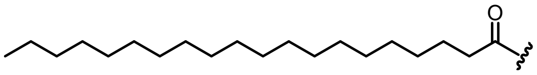	925.72	48
10	C20-0-B	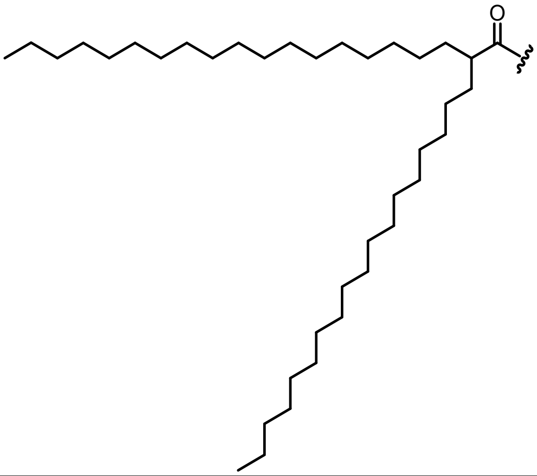	1430.29	38
11	C20-1-A	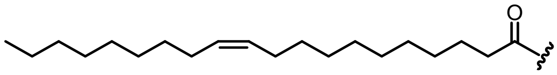	921.69	40
12	C20-2-A	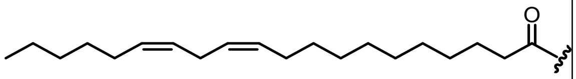	917.66	46

Supplementary Table 2. Characterizations of different LNPs containing various CDLs. Each value represents the mean of 2~3 different experiments.

Ionizable lipid	Additional lipid	Z-average (nm)	PdI	Zeta-potential (mV)	EE%	pKa
MC3	-	151.4	0.079	3.13	100.0	6.28
MC3	Ctrl	135.5	0.065	-6.15	96.4	5.82
MC3	CDL1 (C16-0-A)	156.0	0.116	-3.01	92.2	6.29
MC3	CDL3 (C16-1-A)	148.4	0.058	-0.69	92.2	6.01
MC3	CDL5 (C18-0-A)	157.5	0.157	-0.60	92.2	6.36
MC3	CDL6 (C18-0-B)	163.9	0.108	-1.17	97.7	5.86
MC3	CDL7 (C18-1-A)	162.3	0.069	-1.19	92.1	6.26
MC3	CDL8 (C18-2-A)	131.8	0.109	-3.03	89.0	6.17
MC3	CDL9 (C20-0-A)	150.9	0.071	-4.35	88.9	6.45
MC3	CDL10 (C20-0-B)	141.9	0.122	3.09	97.5	6.28
MC3	CDL11 (C20-1-A)	153.9	0.045	-2.39	90.2	6.22
MC3	CDL12 (C20-2-A)	143.8	0.098	0.29	92.4	6.29
SM102	-	133.1	0.100	2.79	100.0	6.85
SM102	Ctrl	130.5	0.080	-7.07	98.5	6.25
SM102	CDL1 (C16-0-A)	145.4	0.102	-1.50	95.9	6.72
SM102	CDL3 (C16-1-A)	104.5	0.073	-2.78	94.1	6.67
SM102	CDL5 (C18-0-A)	167.4	0.119	1.25	94.9	6.93
SM102	CDL6 (C18-0-B)	189.1	0.091	1.66	97.9	6.28
SM102	CDL7 (C18-1-A)	156.7	0.018	-3.37	100.0	6.84
SM102	CDL8 (C18-2-A)	89.7	0.139	-3.70	96.0	6.78
SM102	CDL9 (C20-0-A)	115.1	0.038	-1.78	92.1	6.87
SM102	CDL10 (C20-0-B)	160.0	0.118	4.12	87.2	6.78
SM102	CDL11 (C20-1-A)	152.8	0.063	-0.83	96.4	6.65
SM102	CDL12 (C20-2-A)	147.3	0.079	-0.48	95.7	6.71

Supplementary Fig 1. Heatmaps of cell viability and NLuc expression for different LNP formulations. RLU; relative light unit.

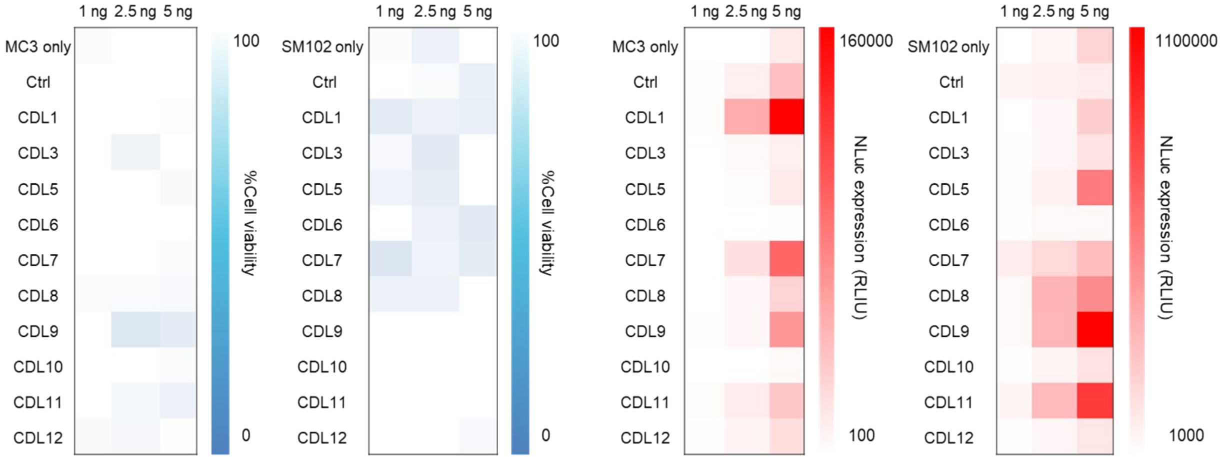

Supplementary Fig 2. Effect of the type of phospholipid on the delivery efficiency of CDL-incorporated LNPs. RLU; relative light unit.

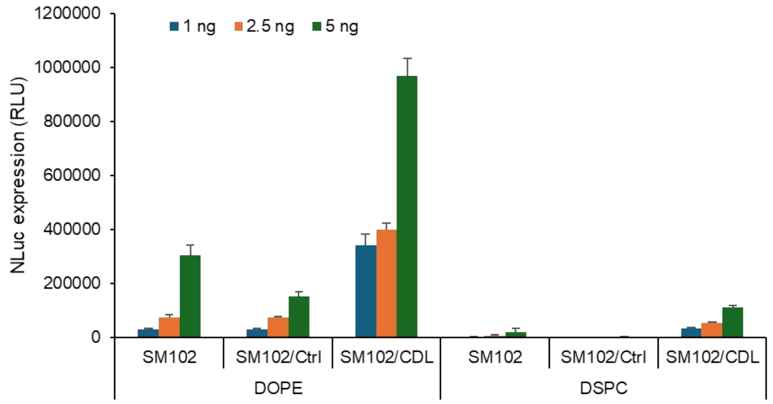

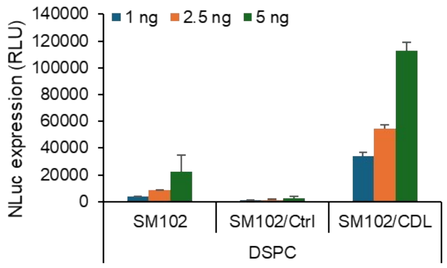

Supplementary Fig 3. Correlation between cellular uptake (DiD MFI) and NLuc expression (RLU). Each dot represents the mean value of 3 different experiments.

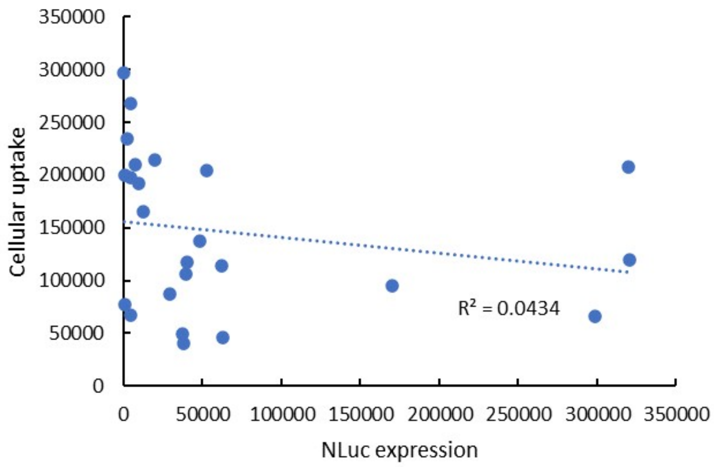

Supplementary Fig 4. Lysosomal colocalization of different LNPs. HeLa cells were treated with DiD-labeled LNPs. At 4 hours after LNP addition CLSM observation was conducted. (A) and (B) show representative images for SM102, SM102/CDL9 LNP formulations, and MC3, MC3/CDL1 LNP formulations, respectively. Line profiles are shown in (C) and (D). Red: LNP (DiD), Green: Lysosome (Lysosensor), Blue: Nucleus (Hoechst), a.u.; arbitrary unit.

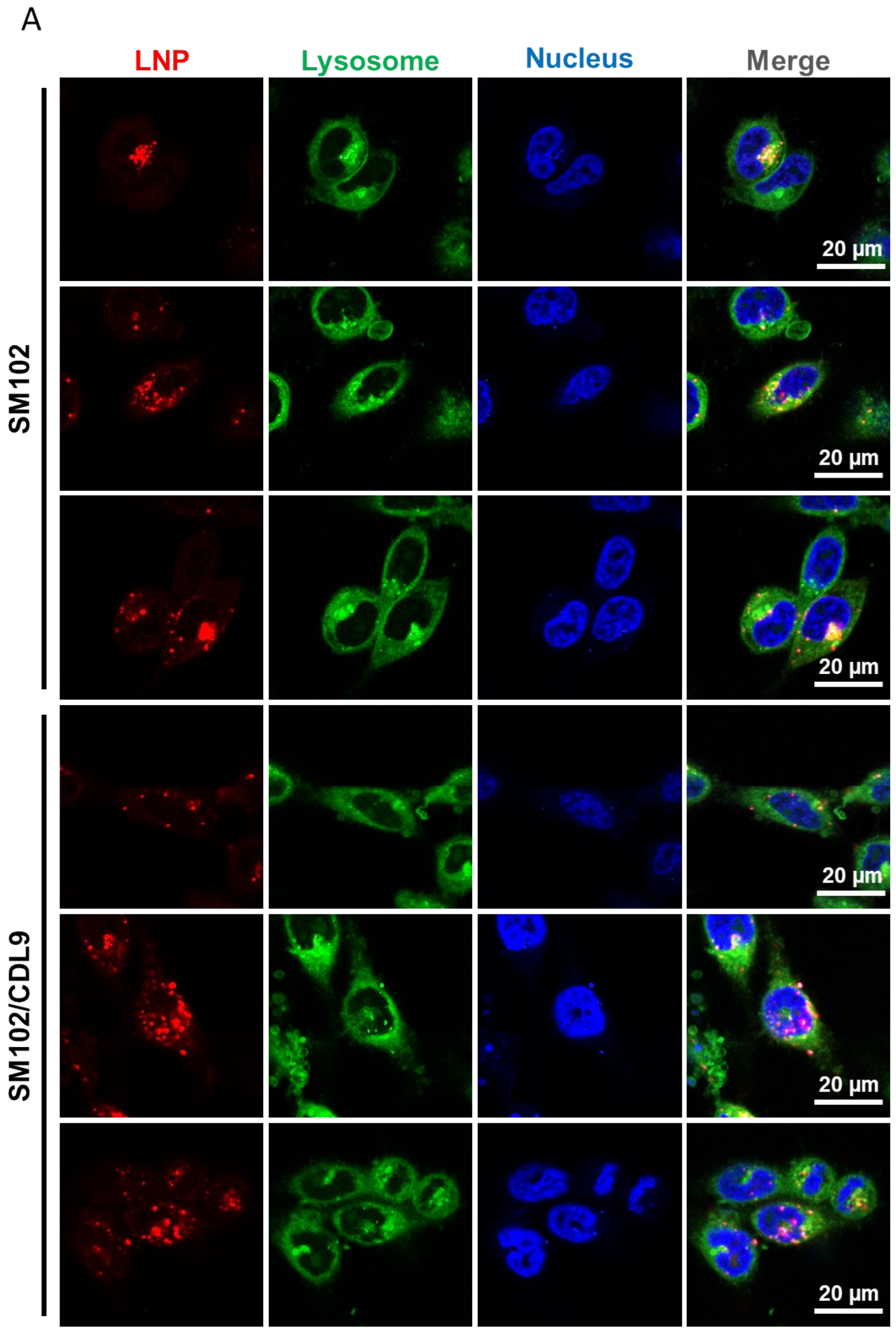

B

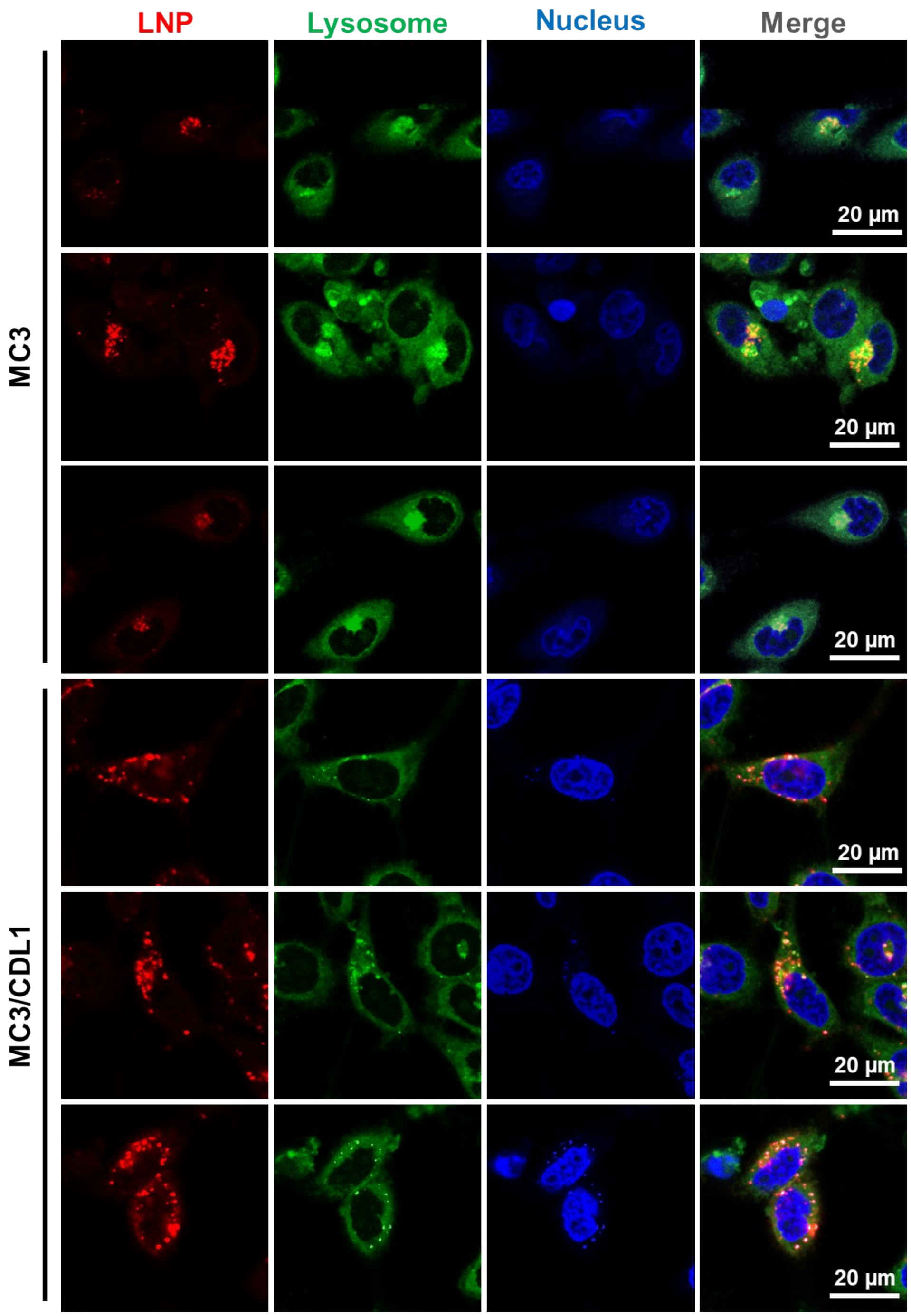

LNP
Lysosome
Nucleus
Merge
MC3
MC3/CDL1
20 μm
20 μm
20 μm
20 μm
20 μm
20 μm

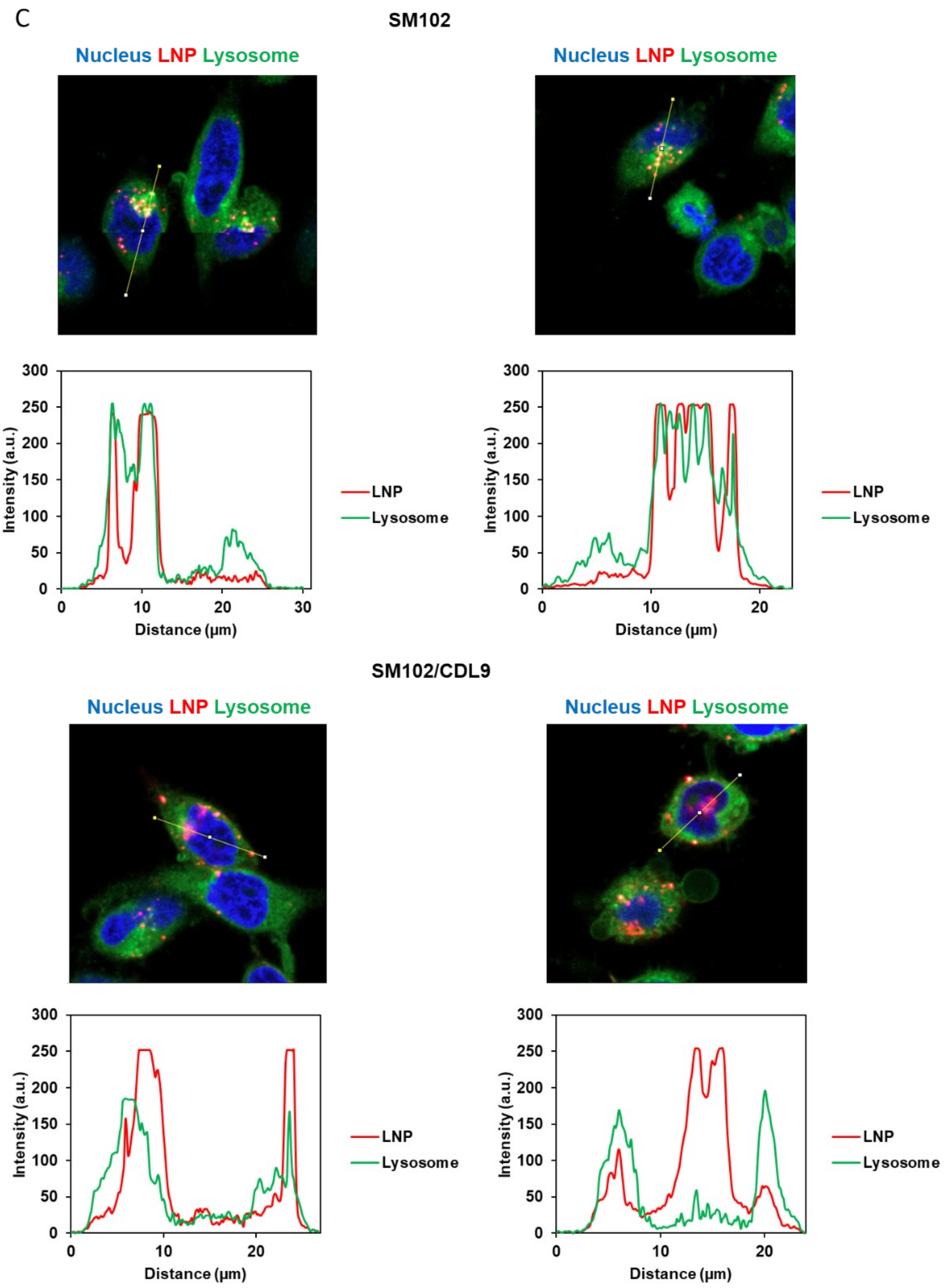
C
SM102
Nucleus LNP Lysosome
Nucleus LNP Lysosome
300
250
200
150
100
50
0
Intensity (a.u.)
0
10
20
30
Distance (µm)
LNP
Lysosome
0
10
20
Distance (µm)
SM102/CDL9
Nucleus LNP Lysosome
Nucleus LNP Lysosome
Intensity (a.u.)
Distance (µm)
LNP
Lysosome

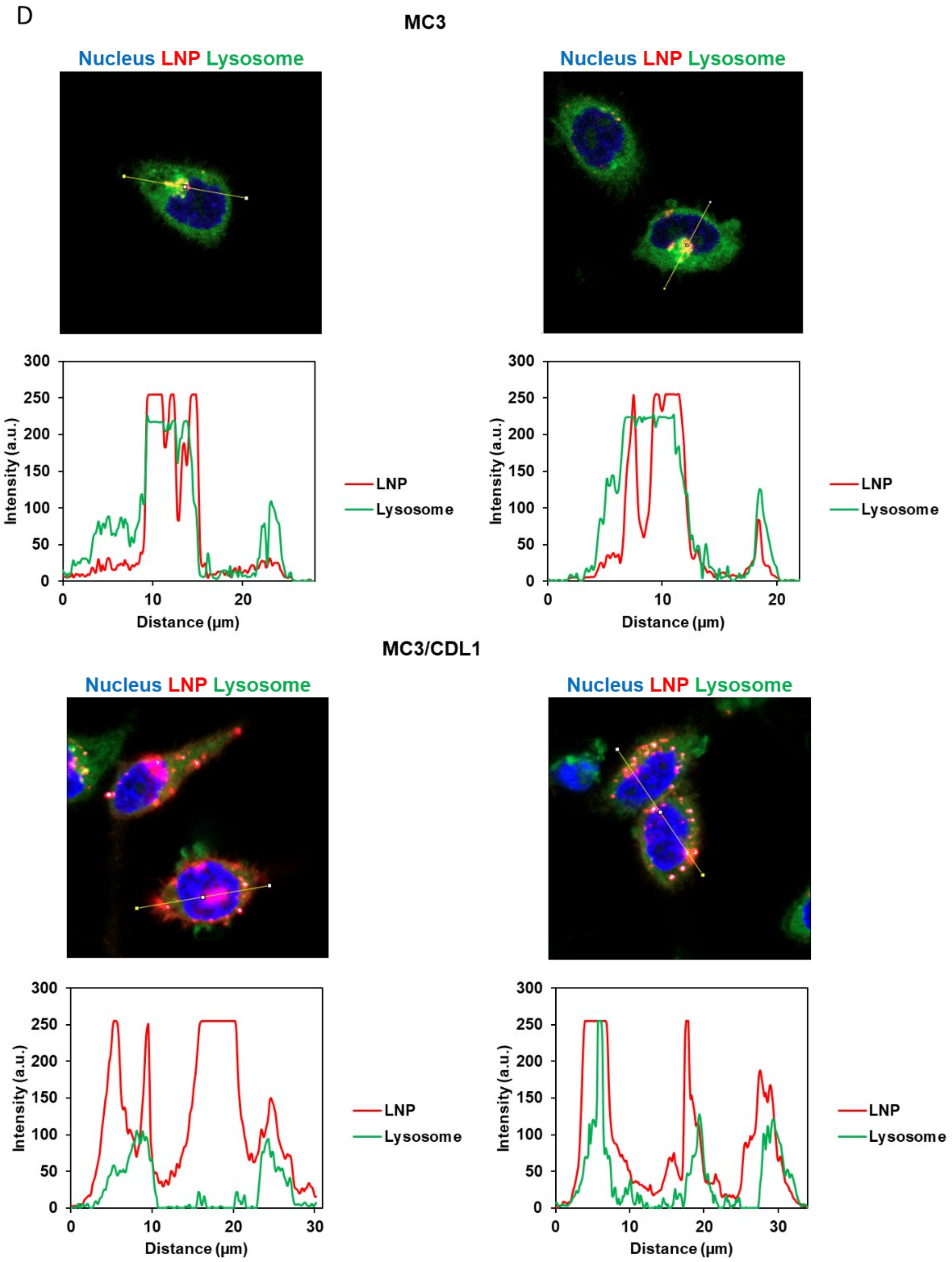
D
MC3
Nucleus LNP Lysosome
Nucleus LNP Lysosome
Intensity (a.u.)
Distance (µm)
LNP
Lysosome
MC3/CDL1
Nucleus LNP Lysosome
Nucleus LNP Lysosome
Intensity (a.u.)
Distance (µm)
LNP
Lysosome

Supplementary Fig 5. Hemolysis assay. Whole blood was collected from ICR mice in the presence of 2 µL of heparin (100 mg/mL). Red blood cells (RBCs) were isolated by diluting 1 mL of whole blood with 9 mL of saline and centrifuging at 400 × g for 5 min at 4 °C. The supernatant was discarded, and the washing step was repeated five times to thoroughly remove serum proteins. Purified RBCs were then incubated with LNPs at pH 7.5, 6.5, or 4.5 for 30 min at 37 °C. Following incubation, samples were centrifuged again at 400 × g for 5 min at 4 °C, and hemolysis was assessed by measuring the absorbance of released hemoglobin at 545 nm. RBCs treated with 0.25 wt/vol% Triton X-100 served as a positive control for complete lysis. The effects of total lipid concentration (A) and serum presence (B) on hemolytic activity were evaluated. Each bar represents the mean ± standard deviation (SD) from three independent experiments. Statistical significance was determined using the Tukey-Kramer test: *p < 0.05, **p < 0.01, ***p < 0.001, ****p < 0.0001.

A

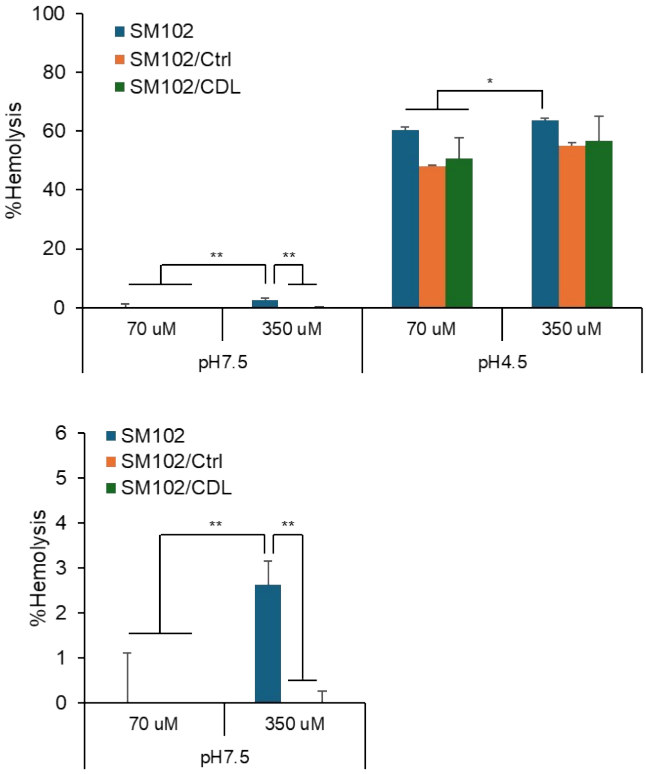

B

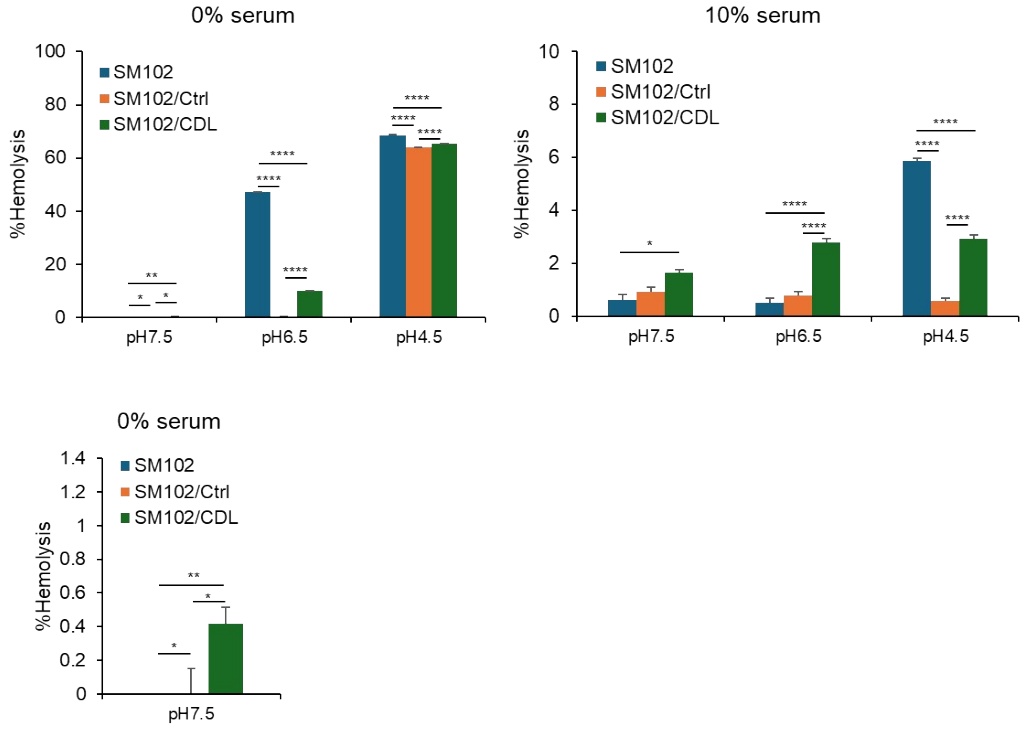
0% serum
100
80
60
40
20
0
%Hemolysis
SM102
SM102/Ctrl
SM102/CDL
pH7.5
pH6.5
pH4.5
10% serum
10
8
6
4
2
0
%Hemolysis
SM102
SM102/Ctrl
SM102/CDL
pH7.5
pH6.5
pH4.5
0% serum
1.4
1.2
1
0.8
0.6
0.4
0.2
0
%Hemolysis
SM102
SM102/Ctrl
SM102/CDL
pH7.5

Supplementary Fig 6. Tumor volume for each mouse in each group (5 mice/group).
The ratio of the number of mice in which no tumors were observed: PBS (0/5), Naked mRNA (2/5), SM102 (5/5), SM102/CDL9 (5/5).

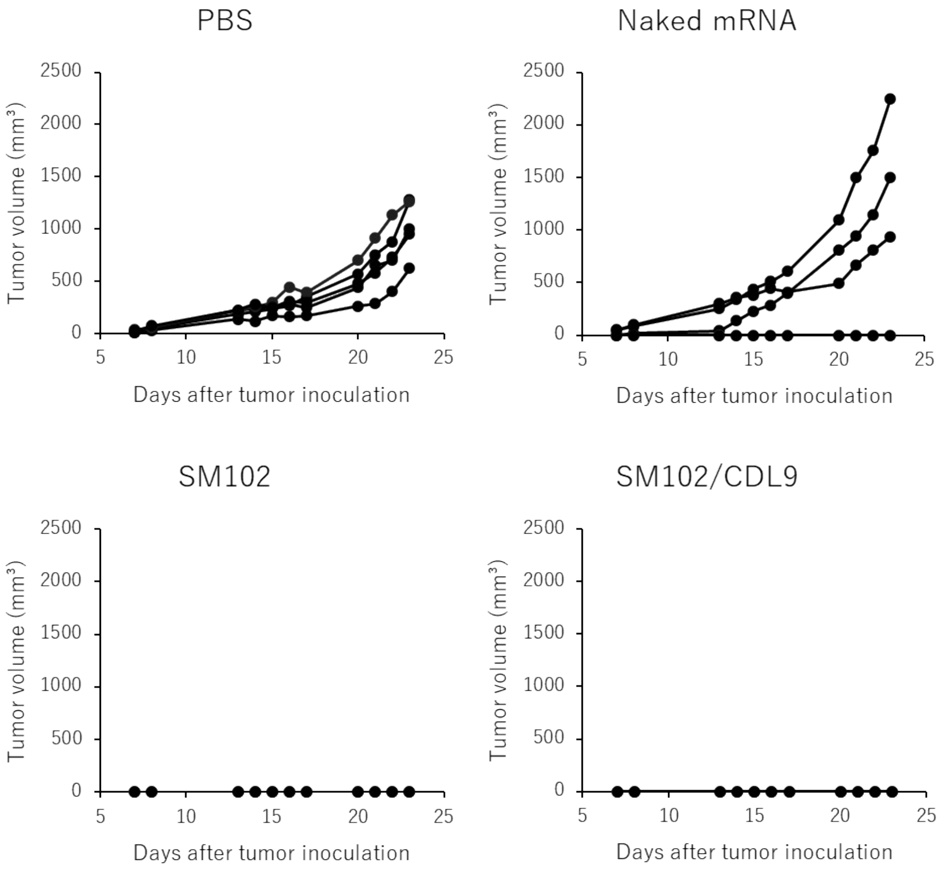

Supplementary Fig 7. Gating strategy for evaluating OVA-specific CD8+T cell populations in the spleen. Representative dot plots for a PBS sample.

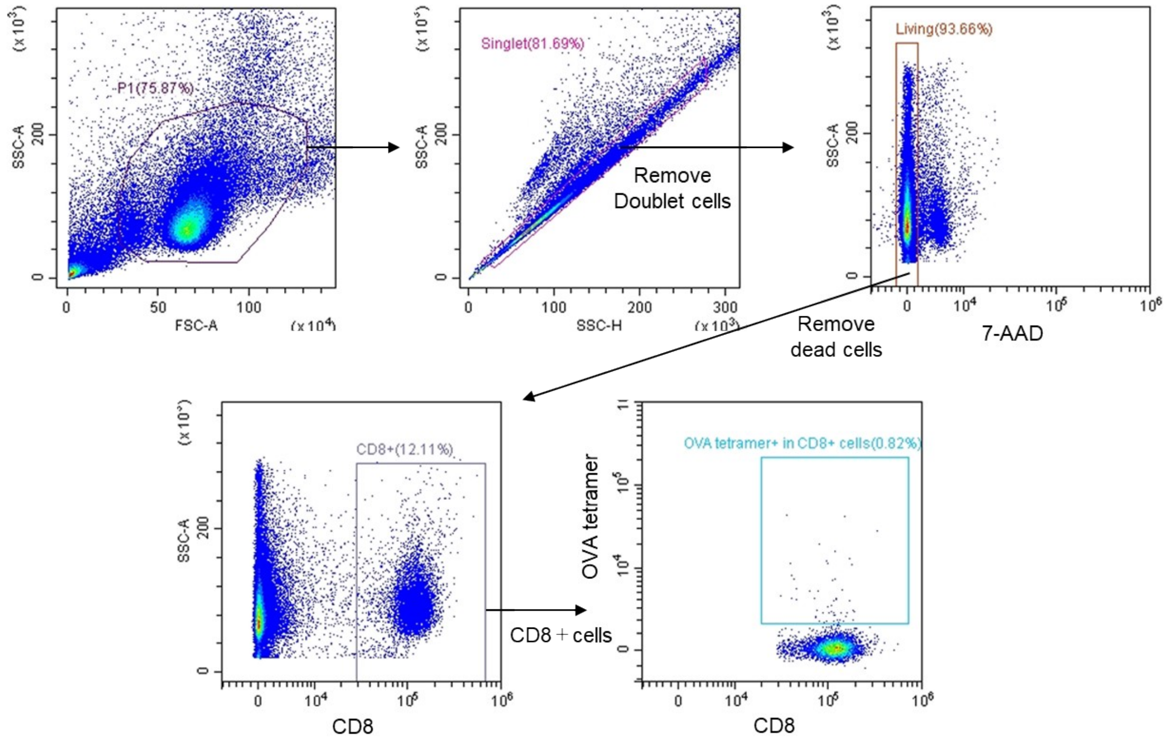

Supplementary Fig 8. Single-dose tolerability study. (A) Schematic procedure for measuring serum IL-6 level and monitoring mouse body weight change. (B) Body wight change after single-dose subcutaneous injection of LNP-mRNA. (C) The level of serum IL-6 at 4 hours after the LNP administration. Dots and bars express mean values + SD of N=3.

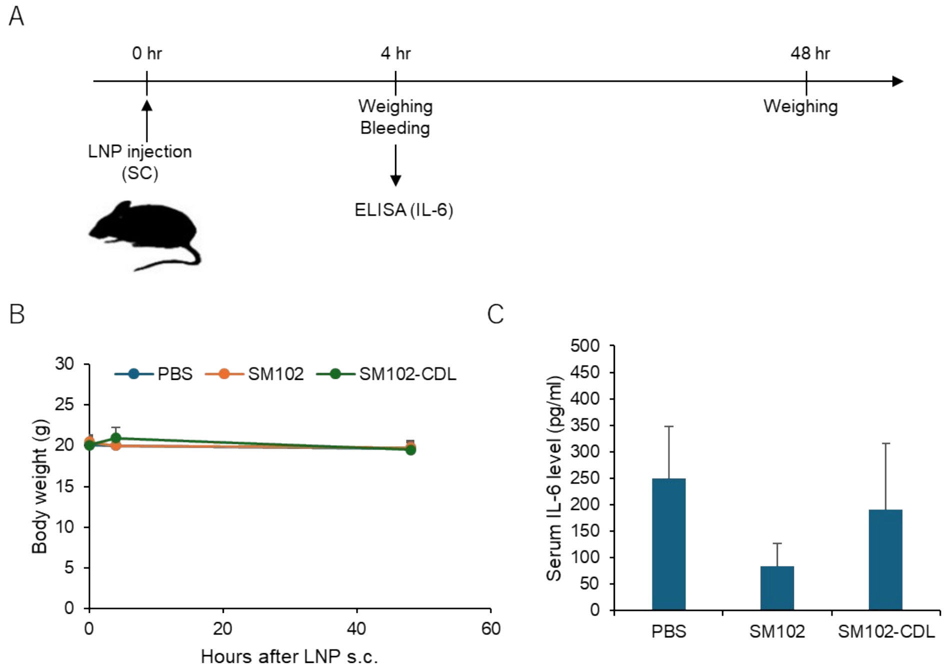

Supplement: MD-016-D5MD00084J-s001 [file MD-016-D5MD00084J-s001.pdf]
